# Cholyl 1,3,4-oxadiazole hybrid compounds: design, synthesis and antimicrobial assessment

**DOI:** 10.3762/bjoc.18.63

**Published:** 2022-05-31

**Authors:** Anas J Rasras, Mohamed El-Naggar, Nesreen A Safwat, Raed A Al-Qawasmeh

**Affiliations:** 1 Faculty of Science, Department of Chemistry, Al-Balqa Applied University, PO Box 19117, Al-Salt, Jordan; 2 College of Sciences, Department of Chemistry, University of Sharjah, Pure and Applied, Chemistry Research Group, PO Box 27272, Sharjah, United Arab Emirates; 3 The Regional Center for Mycology and Biotechnology, Al-Azhar University, Nasr City, Cairo, 11371, Egypt; 4 Department of Chemistry, The University of Jordan, Amman 11942, Jordan

**Keywords:** antibacterial, cholic acid, heterocyclic, Mannich reaction, oxadiazole

## Abstract

A new chemical library based on the hybridization of cholic acid with the heterocyclic moiety 1,3,4-oxadizole was synthesized, and tested for antimicrobial activity against Gram-positive, Gram-negative bacteria, and fungi. Among the synthesized compounds, the most potent derivatives against *S. aureus* were **4t**, **4i**, **4p**, and **4c** with MIC values between 31 and 70 µg/mL, while compound **4p** was the most active one against *Bacillus subtilis* with a MIC value of 70 µg/mL. Interestingly, compounds **4a** and **4u** exerted selective activity against Gram-positive bacteria. The synthesized compounds showed good activity against *A. fumigatus* and *C. albicans* and compound **4v** exhibited selective activity against fungi only.

## Introduction

**Figure 1:**
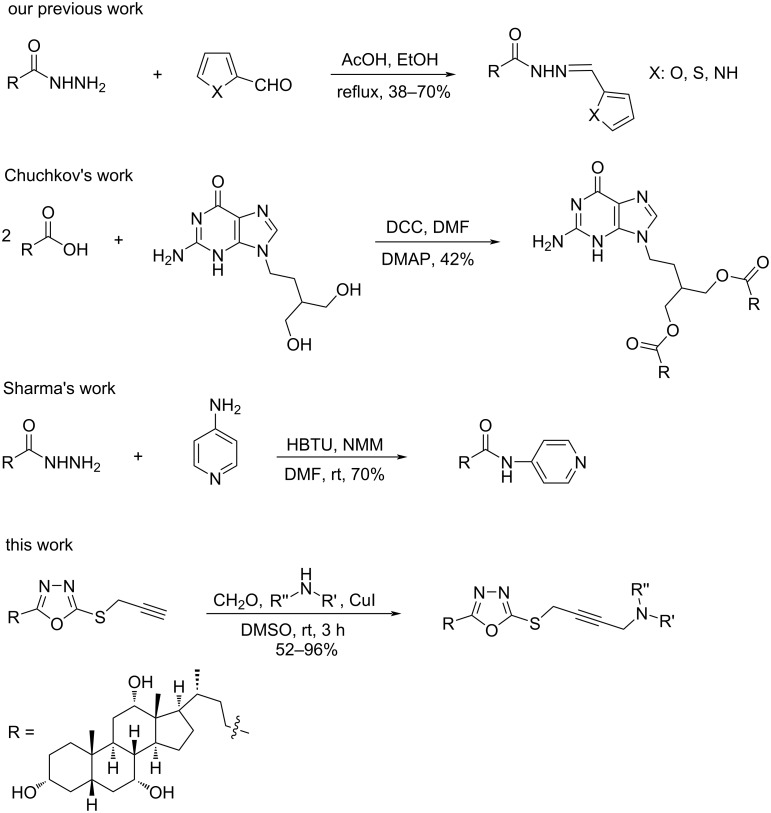
Biologically active cholic acid hybridized with different heterocyclic scaffolds.

Microbial infections caused by Gram-negative and Gram-positive bacteria embarrass the health care system worldwide [1]. Pathogens such as *Escherichia coli*, *Staphylococcus aureus*, *Klebsiella pneumoniae*, and *Staphylococcus pneumoniae* were responsible for most of bacteremia deaths related to antimicrobial resistance in 2019 [2]. Current antibacterial drugs are facing various challenges, due to the inability to accumulate inside human cells made them inactive [3] and the development of multidrug resistant bacteria due to excessive use of antibiotics [2,4]. Heterocyclic compounds are the key components for drug design and synthesis. Among them, 1,3,4-oxadiazole derivatives are attractive and have been investigated for decades. This is due to their promising biological activities such as anti-COVID-19 [5], anticancer [6–8], antibacterial activity against *Staphylococcus aureus* and *Bacillus subtilis* [9–10], antifungal agents against *Candida albicans* and *phytopathogenic fungi* [11–12], and antiproliferative against different cell lines (e.g., PC3, HCT-116, and MCF7) [13]. In 2008, Muhi-eldeen et al, synthesized a hybrid compound with 1,3,4-oxadiazole moiety and pyrrolidine connected with propargylic moiety showed antibacterial activity against *Staphylococcus aureus* and *E. coli* [14]. On the other hand, the coupling of piperazine with heterocyclic compounds enhanced the biological activities like anticancer [15–16], antibacterial [17], antimalarial [18], anti-inflammatory [19], and lead to a promising scaffold for the treatment of Alzheimer’s disease [20]. Our previous work showed that a combination between cholic acid and heterocyclic scaffolds improved the antibacterial property (Figure 1) [21]. In 2018, Sharma et al. presented a new pyridinyl-substituted cholic acid analogue that was effective against an epidemic strain of *Clostridium difficile* (Figure 1) [22]. Recently, Chuchkov et al. prepared a hybrid structure between heterocycle penciclovir and cholic acid, and the product showed antiviral activity (Figure 1) [23]. In continuation of our ongoing research on designing compounds with potential biological activities, we herein report the design, synthesis, and antimicrobial assessment of novel cholyl 1,3,4-oxadiazole moieties (Figure 1).

For developing new drugs, cholic acid with its unique shape has attracted scientists’ attention by virtue of its non-toxic, natural human product, biodegradable, and amphiphilic properties. Cholic acid derivatives have been reported to have a wide range of activities such as antibacterial [21,24–26] and anticancer [27–29], and were used for ischemic stroke treatment [30], to decrease the cytotoxicity of anticancer drugs [31], and as amphiphilic copolymers as artificial ionophores [32].

## Result and Discussion

**Scheme 1:**
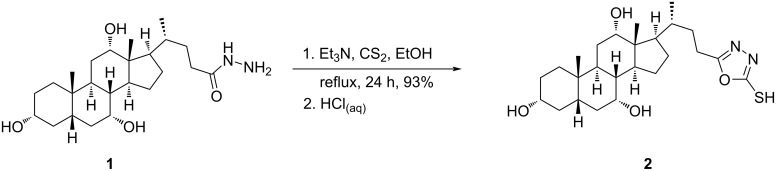
Synthesis of cholyl 1,3,4-oxadiazole-2-thiol **2**.

The synthetic strategy for the synthesis of the desired compounds **4a**–**v** commenced from commercially available cholic acid, which was converted to its cholyl hydrazide (**1**) as previously reported by us [21]. The produced cholyl hydrazide **1** was heterocyclized to 1,3,4-oxadiazole-2-thiol **2** in excellent yield (93%), via the treatment with carbon disulfide and trimethylamine in refluxing ethanol (Scheme 1) [33].

**Scheme 2:**
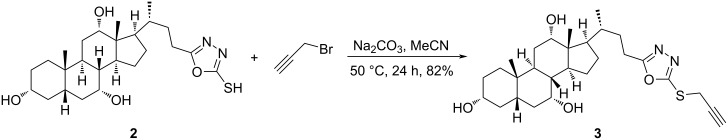
Synthesis of cholyl 2-(propargylthio)-1,3,4-oxadiazole **3**.

Having oxadiazole-2-thiol **2** at hands, the reactive thiol was subjected to the reaction with propargyl bromide and sodium carbonate as a base to afford the thiopropargylated derivative **3** in 82% yield after 24 h (Scheme 2) [33].

**Scheme 3:**
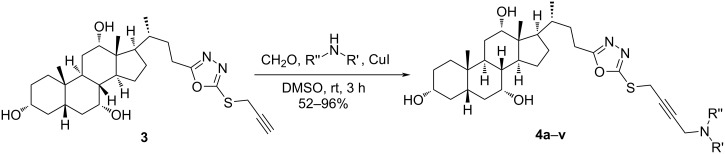
Synthesis of target compounds **4a**–**v**.

Compound **3** was the starting point for a Mannich reaction to generate a library of 22 diverse compounds. Briefly, the alkyne **3** was treated with formaldehyde, a secondary amine, and CuI as catalyst in DMSO (Scheme 3). The three components were stirred at room temperature for 3 h to furnish the desired compounds **4a**–**v** in moderate to excellent yields [14,34].

**Figure 2:**
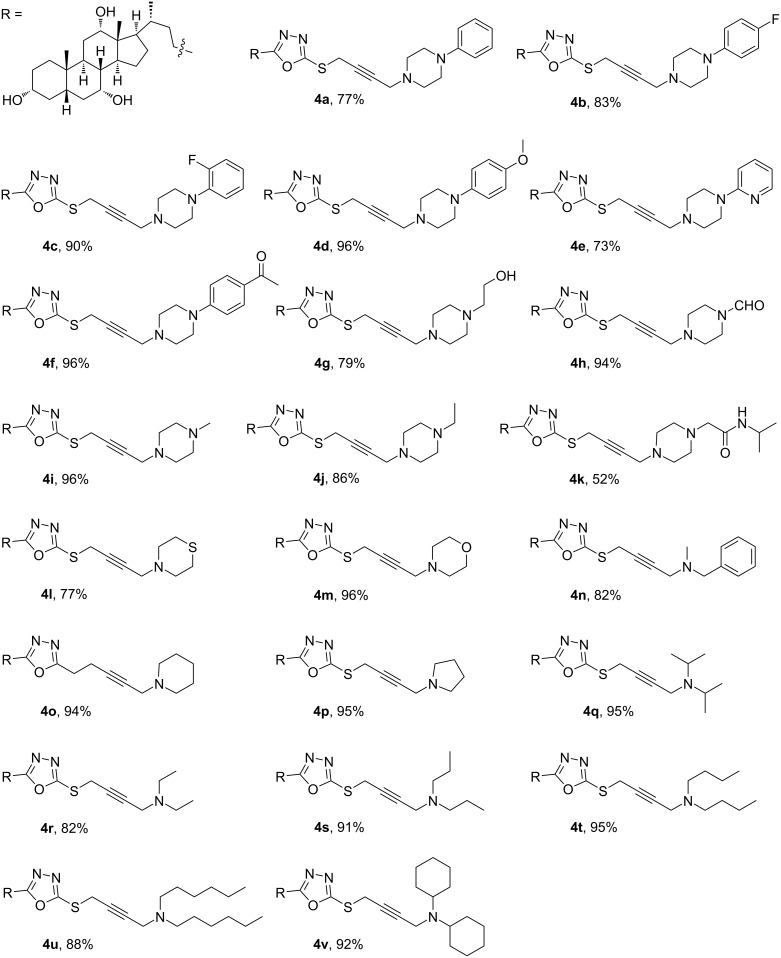
Structures of target compounds **4a**–**v**.

By this route, diverse products derived from piperazine derivatives with aromatic electron-donating (**4d**), electron-withdrawing (**4b**, **4c**, and **4f**), and aliphatic groups (**4g**, **4i**, and **4j**) were obtained. Moreover, the reaction with secondary aliphatic amines with various alkyl chains afforded products **4r**–**u**, whereas products **4o** and **4p** were obtained from piperidine and pyrrolidine, respectively, as secondary cyclic amine component (Figure 2).

The structures of the newly synthesized compounds were confirmed on the basis of their spectral data in particular nuclear magnetic resonance (NMR) and mass spectrometry (MS) techniques. The ^1^H NMR spectra (CDCl_3_) for the synthesized compounds showed complex protons in the aliphatic region which correspond to the cholyl moiety in the range of 1.3–2.0 ppm and aliphatic amine protons. The S–CH_2_ protons appeared as a singlet in all compounds at about δ = 4.00 ppm, the hydroxy protons were not observed in most of the compounds except for derivatives **4b**, **4d**, **4p**, and **4u**. All aromatic compounds showed resonances at δ = 6.50–8.00 ppm. Compounds **4b** and **4c** indicated the fluorine coupling effect on the aromatic protons. The carbaldehyde proton in compound **4h** resonates at δ = 8.00 ppm, while compound **4k** showed an amide doublet resonance at 7.09 ppm. On the other hand, the ^13^C NMR spectra showed all characteristic signals for all of the synthesized compounds, with multiple aliphatic peaks for the cholyl and aliphatic amine moieties. The fingerprint signals for the cholyl moiety (C–OH) were evident in all spectra of the synthesized compounds resonating at around δ = 68.0, 72.0, and 73.0 ppm. All aromatic compounds showed clear and correct carbon signals in the aromatic region. The two alkyne carbon atoms can be recognized for most of the compounds, while the other compounds had week signals. The two quaternary oxadiazole peaks appeared at around δ = 162.0 and 169.0 ppm. Compound **4f** showed a carbonyl peak at δ = 196.7 ppm and for the carbaldehyde carbon in compound **4h** a peak at δ = 160.8 ppm was observed. To further characterize the structures, 2D NMR experiments were done for compound **4p** as example. The HMQC experiment revealed a correlation between the CH–O protons at 3.37, 3.79, and 3.91 ppm, and the carbon atoms at 72.0, 68.6, and 73.2 ppm, respectively. Moreover, the CH_2_S proton at 3.99 ppm correlated with carbon at 21.7 ppm. The methylene protons in -CH_2_-N-pyrrolidine at 3.51 ppm correlated with carbon at 42.7 ppm (see Supporting Information File 1). A COSY experiment for compound **4p** showed long correlation between the two singlet methylene CH_2_S at 4.00 ppm and -CH_2_-N-pyrrolidine at 3.51 ppm (Supporting Information File 1).

### Antimicrobial activity

The newly synthesized compounds were evaluated for their in vitro antibacterial potential against *Staphylococcus aureus* and *Bacillus subtilis* as examples of Gram-positive bacteria as well as against *Escherichia coli* and *Proteus vulgaris* as examples of Gram-negative bacteria [35]. They were also evaluated for their in vitro antifungal activity against the pathogenic fungal strains *Aspergillus fumigatus* and *Candida albicans*. The sensitivity of the organisms was assayed against the activity of tested compounds solutions (at 10 mg/mL concentration) using a modified agar well diffusion method with determination of the inhibition zone diameter in mm as criterion for antimicrobial activity. As shown by the results of antimicrobial activity testing (Table 1), the newly synthesized compounds revealed good in vitro antibacterial and antifungal activities. However, compounds **4t**, **4i**, **4p** and **4c** showed the highest activity against Gram-positive bacteria *Staphylococcus aureus* in the range of 33–36 mm. Similarly, it can be seen that compound **4p** showed the highest activity (26.7 mm) against Gram-positive bacteria *Bacillus subtilis* followed by compounds **4i**, **4o**, **4j**, **4q**, **4r**, **4g**, **4m**, **4c**, **4t**, **4h**, **4d**, **4l**, **4b**, **4e**, **4s**, **4k**, **4u**, and **4a**, respectively (Table 1). Furthermore, compound **4d** showed the highest activity against Gram-negative bacteria *Proteus vulgaris* followed by compounds **4c**, **4t**, **4b**, **4n**, **4s**, **4l**, **4p**, **4q**, **4i**, **4o**, **4g** and **4j**, respectively. All tested compounds exhibited lower activities compared to the tested reference drugs.

On the other hand, the order of antibacterial activity against *Escherichia coli* was **4c**, **4p**, **4d**, **4i**, **4g**, **4t**, **4q**, **4b**, **4j**, **4o**, **4n**, **4s**, **4r**, and **4l**, respectively (Table 1). Moreover, compound **4i** exhibited the highest activity against the pathogenic filamentous fungus *Aspergillus fumigatus* followed by compounds **4s**, **4p**, **4j**, **4q**, **4o**, **4g**, **4k**, **4t**, **4m**, **4p** and **4c**, respectively. Besides, the order of antifungal activity against the pathogenic yeast *Candida albicans* was **4g**, **4i**, **4q**, **4j**, **4o**, **4p**, **4m**, **4k**, **4s**, **4t**, **4n**, **4v**, **4d**, **4e**, **4h**, **4l**, **4c** and **4r**, respectively (Table 1). Likewise, no antimicrobial activities could be detected for compound **4f** under these screening conditions (Table 1). Interestingly, compounds **4c, 4d, 4g**, **4i**, **4**j, **4l**, **4n**, **4o**, **4p**, **4q**, **4r**, **4s**, and **4t** exhibited broad spectrum antibacterial and antifungal activities, showing their variable inhibitory activities against multiple microorganisms.

**Table 1:** In vitro antimicrobial activities of the synthesized compounds tested at 10 mg/mL by modified well diffusion agar method and expressed as mean inhibition zone diameter (mm).

compound	tested microorganisms^a^					

	fungi		Gram-positive bacteria		Gram-negative bacteria	

	*C. albicans* ATCC 10231	*A. fumigatus* ATCC MYA-4609	*S. aureus* ATCC 6538	*B. subtilis* NRRL-B-543	*E. coli* ATCC 25955	*P. vulgaris* ATCC 13315

**4a**	n.a	n.a	12.3 ± 0.9	8.9 ± 0.7	n.a	n.a
**4b**	n.a	n.a	25.6 ± 1.8	16.2 ± 1.4	12.4 ± 1.2	17.3 ± 1.5
**4c**	9.1 ± 0.7	10.2 ± 0.8	33.4 ± 1.2	19.1 ± 1.3	17.8 ± 0.9	21.2 ± 1.6
**4d**	11.9 ± 1.1	10.8 ± 0.6	25.1 ± 0.8	17.5 ± 1.4	15.2 ± 0.9	22.3 ± 1.7
**4e**	11.2 ± 0.9	8.9 ± 0.7	30.3 ± 1.6	16.1 ± 1.5	n.a	n.a
**4f**	n.a	n.a	n.a	n.a	n.a	n.a
**4g**	18.9 ± 1.5	15.1 ± 1.2	13.3 ± 0.9	20.9 ± 1.3	14.2 ± 1.1	8.9 ± 1.3
**4h**	11.2 ± 0.8	n.a	11.4 ± 0.8	18.3 ± 1.1	n.a	n.a
**4i**	18.9 ± 1.2	18.3 ± 1.5	35.3 ± 1.9	24.3 ± 1.7	15.1 ± 0.5	9.4 ± 1.2
**4j**	17.8 ± 1.4	15.6 ± 1.3	30.1 ± 1.3	23.2 ± 1.6	12.4 ± 0.8	8.3 ± 0.9
**4k**	16.1 ± 1.3	13.2 ± 1.4	12.4 ± 1.6	15.3 ± 1.1	n.a	n.a
**4l**	10.1 ± 0.9	9.2 ± 0.7	17.8 ± 1.4	17.2 ± 1.5	11.2 ± 1.3	14.5 ± 1.7
**4m**	16.4 ± 0.8	13.1 ± 1.2	14.3 ± 1.5	19.4 ± 1.4	n.a	n.a
**4n**	13.3 ± 1.1	9.8 ± 0.4	28.2 ± 1.6	17.0 ± 1.2	12.3 ± 0.9	16.4 ± 1.4
**4o**	17.6 ± 1.4	15.3 ± 1.1	22.1 ± 1.7	24.2 ± 1.6	12.3 ± 1.1	9.3 ± 0.9
**4p**	16.7 ± 1.3	16.2 ± 1.4	33.5 ± 1.9	26.7 ± 1.8	15.4 ± 1.2	11.7 ± 0.8
**4q**	18.2 ± 1.4	15.4 ± 1.1	29.8 ± 1.4	22.7 ± 1.5	13.5 ± 0.7	10.1 ± 0.9
**4r**	9.1 ± 0.7	7.8 ± 1.2	28.2 ± 1.4	21.2 ± 1.6	11.3 ± 0.9	7.4 ± 0.8
**4s**	15.6 ± 1.2	16.7 ± 1.5	31.4 ± 1.5	15.6 ± 1.3	12.1 ± 0.7	16.2 ± 0.8
**4t**	13.4 ± 1.5	13.1 ± 1.3	36.2 ± 1.9	19.1 ± 0.7	14.2 ± 0.9	20.9 ± 1.1
**4u**	n.a	n.a	14.5 ± 1.1	10.2 ± 0.6	n.a	n.a

**4v**	12.3 ± 1.4	10.2 ± 0.6	n.a	n.a	n.a	n.a

ketoconazole^b^	25.7 ± 1.5	26.2 ± 1.6	–	–	–	–

gentamycin^b^	–	–	31.9 ± 1.7	33.1 ± 1.9	29.5 ± 1.3	28.8 ± 1.6

^a^The data are expressed as inhibition zone diameter (mm) in the form of mean ± standard error (where well diameter 6 mm); n.a.: not active. ^b^Ketoconazole and gentamycin were used (at 1 mg/mL conc.) as standard drugs against the tested fungi and bacteria, respectively.

The antimicrobial efficiency of the tested compounds was confirmed by the MIC values measured by the broth microdilution method by recording the lowest concentration that showed inhibition of microbial growth (Table 2). The results of the determined MIC values showed the same trend of the antimicrobial activities explored by determination of the inhibition zone diameter using the agar well diffusion method.

**Table 2:** Minimum inhibitory concentrations (MIC, µg/mL) of the synthesized compounds determined by microdilution method.

compound	^a^tested microorganisms					

	fungi		Gram-positive bacteria		Gram-negative bacteria	

	*C. albicans* ATCC 10231	*A. fumigatus* ATCC MYA-4609	*S. aureus* ATCC 6538	*B. subtilis* NRRL-B-543	*E. coli* ATCC 25955	*P. vulgaris* ATCC 13315
	
**4a**	n.a	n.a	1500 ± 559	6000 ± 2236	n.a	n.a
**4b**	n.a	n.a	141 ± 35	563 ± 140	1250 ± 294	500 ± 171
**4c**	4500 ± 1118	4000 ± 1369	70 ± 17	281 ± 70	438 ± 171	313 ± 65
**4d**	2250 ± 559	3000 ± 1118	250 ± 86	563 ± 140	750 ± 280	281 ± 70
**4e**	3000 ± 726	6000 ± 2236	125 ± 42	750 ± 135	n.a	n.a
**4g**	375 ± 140	1000 ± 342	3500 ± 1369	281 ± 70	2250 ± 559	7000 ± 2739
**4h**	3000 ± 726	n.a	2250 ± 559	375 ± 140	n.a	n.a
**4i**	281 ± 70	750 ± 280	55 ± 21	125 ± 43	1000 ± 342	4000 ± 1369
**4j**	1125 ± 280	1500 ± 559	109.37 ± 43	438 ± 171	2250 ± 559	6000 ± 2236
**4k**	750 ± 280	3500 ± 1369	2250 ± 559	1250 ± 294	n.a	n.a
**4l**	2250 ± 559	6000 ± 2236	375 ± 140	438 ± 171	3500 ± 1369	2250 ± 559
**4m**	1500 ± 559	3500 ± 1369	2250 ± 559	281 ± 70	n,a	n.a
**4n**	1250 ± 294	4000 ± 1369	141 ± 35	563 ± 139	1125 ± 80	563 ± 140
**4o**	1000 ± 342	1250 ± 294	281 ± 70	141 ± 35	2250 ± 559	4500 ± 1118
**4p**	1125 ± 280	1000 ± 342	63 ± 21	70 ± 17	1250 ± 294	3000 ± 1118
**4q**	438 ± 171	1250 ± 294	125 ± 43	375 ± 140	2250 ± 559	4500 ± 1118
**4r**	4500 ± 1118	7000 ± 2739	125 ± 43	281 ± 70	1500 ± 559	8000 ± 2739
**4s**	3500 ± 1369	1125 ± 280	125 ± 43	1250 ± 294	1125 ± 280	1000 ± 342
**4t**	875 ± 342	2250 ± 559	31 ± 11	281 ± 70	3500 ± 1369	281 ± 70
**4u**	n.a	n.a	750 ± 280	1500 ± 559	n.a	n.a

**4v**	3500 ± 137	4500 ± 112	n.a	n.a	n.a	n.a

ketoconazole	10 ± 2	39 ± 9	–	–	–	–

gentamycin	–	–	5 ± 1	2 ± 1	3 ± 1	5 ± 1

^a^The data are expressed as mean MIC values ± standard error; n.a: not active.

The structure–activity relationship (SAR) elaborated that piperazines with aliphatic groups on the nitrogen atom are more active than those with aromatic substituents against the fungus *C. albicans*. On the other hand, compounds comprising piperazines with fluorinated aromatic (**4b** and **4c**), a pyridinyl moiety (**4e**), and an alkylated piperazine (**4i** and **4j**) were more active against *S. aureus* as well as derivatives with dialkylamino substituents with alkyl groups containing <5 carbon atoms (**4q**, **4r**, **4s**, and **4t**) and pyrrolidine (**4p**). According to the MIC values piperazines with a methyl group (**4i**) and compounds with cyclic amines (**4o** and **4p**) were the most active against *B. subtilis*. Compounds **4a**, **4e, 4h, 4k, 4m**, **4u,** and **4v** showed no activities against the tested Gram-negative bacteria under these screening conditions (Table 2).

## Conclusion

A new chemical library based on the hybridization of cholic acid with the heterocyclic moiety 1,3,4-oxadizole was synthesized. All new compounds were unambiguously characterized by various spectroscopic techniques. The newly synthesized compounds were assessed in vitro for their antimicrobial activities. Compounds **4g** and **4i** showed good antifungal activity against *C. albicans*. Compounds **4t**, **4i**, **4p**, and **4c** were the most active derivatives against *S. aureus* with MIC values between 31 and 70 µg/mL, while compound **4p** showed good activity against *Bacillus subtilis* with a MIC value of 70 µg/mL. Further development of this library will be reported in due course.

## Supporting Information

File 1 Experimental procedures, characterization of products, and copies of NMR spectra.
