# Supplementary material for: Cholyl 1,3,4-oxadiazole hybrid compounds: design, synthesis and antimicrobial assessment
Source: Beilstein J Org Chem. 2022 May 31;18:631–8. doi: 10.3762/bjoc.18.63 (PMC9174839; doi:10.3762/bjoc.18.63)
Supplement: File 1 — Experimental procedures, characterization of products, and copies of NMR spectra. [file Beilstein_J_Org_Chem-18-631-s001.pdf]

BEILSTEIN JOURNAL OF ORGANIC CHEMISTRY

Supporting Information

for

Cholyl 1,3,4-oxadiazole hybrid compounds: design, synthesis and antimicrobial assessment

Anas J. Rasras, Mohamed El-Naggar, Nesreen A. Safwat and Raed A. Al-Qawasmeh

Beilstein J. Org. Chem. **2022,** *18,* 631–638. doi:10.3762/bjoc.18.63

Experimental procedures, characterization of products, and copies of NMR spectra

License and Terms: This is a supporting information file under the terms of the Creative Commons Attribution License (https://creativecommons.org/licenses/by/4.0). Please note that the reuse, redistribution and reproduction in particular requires that the author(s) and source are credited and that individual graphics may be subject to special legal provisions.
The license is subject to the *Beilstein Journal of Organic Chemistry* terms and conditions: (https://www.beilstein-journals.org/bjoc/terms)

Table of contents

1. Experimental section S2

1.1 General information S2

1.2 Procedure for preparation of 1,3,4-oxadiazole-2-thiol (**2**) S2

1.3 Procedure for preparation of 2-(propargylthio)-1,3,4-oxadiazole (**3**) S3

1.4 General procedure for preparation of compounds **4a**–**v** S3

2. Antimicrobial activity assay S14

3. Copies of NMR spectra S16

4. References S65

1. Experimental section

1.1. General information

NMR spectra were measured with tetramethylsilane (TMS) as the internal standard. NMR spectra were recorded on a Bruker Avance III-500 MHz spectrometer type (^{1}H NMR, 500 MHz; ^{13}C NMR, 125 MHz). Chemical shifts (δ) are reported in ppm. High resolution mass spectra (HRMS) were recorded in either positive or negative ion mode by electrospray ionization using a Bruker Daltonics Apex IV, 7.0 T Ultra Shield Plus (Bremen, Germany). Melting points (mp) were determined on an electrothermal melting point apparatus and are uncorrected. Unless otherwise noted, all chemicals and solvents used were obtained from Fluka and Aldrich and used without further purification. THF was distilled over sodium metal and benzophenone. Reactions were monitored by thin layer chromatography (TLC) using Merck aluminum plates precoated with silica gel PF254 (20 × 20 × 0.2 mm) and detected by visualization of the plate under a UV lamp (λ = 254 nm).

1.2. Procedure for preparation of (3*R*,5*S*,7*R*,8*R*,9*S*,10*S*,12*S*,13*R*,14*S*,17*R*)-17-((*R*)-4-(5-mercapto-1,3,4-oxadiazol-2-yl)butan-2-yl)-10,13-dimethylhexadecahydro-1*H*-cyclopenta[*a*]phenanthrene-3,7,12-triol (2)

To a solution of cholylhydrazide **1** (4.10 g, 9.70 mmol, 1.0 equiv) in 50 mL EtOH was added carbon disulfide (1.76 mL, 29.10 mmol, 3.0 equiv) and Et_3N (1.62 mL, 11.64 mmol, 1.2 equiv). The solution was refluxed for 24 h, then cooled to room temperature and acidified with HCl (0.1 M in H_2O) and let stand for 3 h at room temperature. The formed precipitate was filtered and washed with H_2O (3 × 10 mL) and petroleum ether (2 × 10 mL) to give oxadiazole-2-thiol **2** (4.19 g, 8.80 mmol, 93%) as a yellow solid. mp 237-239 °C. ^{1}H NMR (500 MHz, DMSO-d_6): δ 0.59 (s, 3H, CH_3); 0.80 (s, 3H, CH_3); 0.97 (d, 3H, J= 5.2, CH_3); 1.09–2.08 (m, 20H, CH_2 and CH); 2.08-2.33 (m, 2H, CH_2); 2.58-2.64 (m, 1H, CH); 2.66-2.82 (m, 1H, CH); 3.11-3.26 (m, 1H, CH); 3.61 (m, 1H, CH); 3.78 (m, 1H, CH); 3.87-4.37 (br, 3H, OH); 14.27 (s,1H, SH).^{13}C NMR (125 MHz, DMSO-d_6): δ 12.3, 16.8, 22.0, 22.6, 22.8, 26.2, 27.2, 28.5, 30.4, 31.3, 34.4, 34.9, 35.3, 39.6, 39.8, 39.9, 41.4, 41.5, 45.8, 45.9, 66.2, 70.4, 70.9, 164.7, 177.7. HRMS (ESI-MS): m/z calcd for $C_{25}H_{39}N_2O_4S$: 463.2636 $[M-H]^-$; found 463.2637.

1.3. Procedure for preparation of (3*R*,5*S*,7*R*,8*R*,9*S*,10*S*,12*S*,13*R*,14*S*,17*R*)-10,13-dimethyl-17-((*R*)-4-(5-(prop-2-yn-1-ylthio)-1,3,4-oxadiazol-2-yl)butan-2-yl)hexadecahydro-1*H*-cyclopenta[*a*]phenanthrene-3,7,12-triol (3):

To a solution of oxadiazole-2-thiol **2** (6.94 g, 14.93 mmol, 1.0 equiv) in acetonitrile (50 mL) was added Na_2CO_3 (4.75 g, 44.80 mmol, 3 equiv) and the mixture was stirred for 20 min at room temperature followed by heating to 50 °C. Then, 3-bromopropyne solution (80% in toluene, 2.40 mL, 22.40 mmol, 1.5 equiv) was added and the mixture was stirred for 24 h at 50 °C. Na_2CO_3 was removed by filtration and the solvent was evaporated. To the residue 20 mL of water were added and extracted with EtOAc (2 × 20 mL). The organic layer was washed with water (2 × 20 mL), dried over $MgSO_4$, and evaporated in vacuum yielding compound **3** (6.15 g, 12.23 mmol, 82%) as a pale brown solid. mp 155-157 °C. ^{1}H NMR (500 MHz, $CDCl_3$): δ 0.67 (s, 3H, CH_3); 0.88 (s, 3H, CH_3); 1.04 (d, 3H, J= 6.4, CH_3); 1.32–2.04 (m, 20H, CH_2 and CH); 2.11-2.27 (m, 2H, CH); 2.31 (t, 1H, J= 2.5, alkyne); 2.71-2.80 (m, 1H, CH); 2.84-2.94 (m, 1H, CH); 3.38-3.52 (m, 1H, CH); 3.85 (m, 1H, CH); 3.97 (m, 3H, CH_2 and CH).^{13}C NMR (125 MHz, $CDCl_3$): δ 12.5, 17.3, 21.0, 22.4, 22.4, 23.2, 26.4, 27.5, 28.2, 29.7, 30.2, 32.4, 34.5, 34.7, 35.2, 39.4, 39.4, 41.4, 41.7, 46.5, 46.7, 68.5, 72.1, 72.9, 73.0, 77.4, 162.3, 169.1. HRMS (ESI-MS): m/z calcd for $C_{28}H_{42}N_2NaO_4S$: 525.2758 $[M+Na]^+$; found 525.2756.

1.4. General procedure for preparation of compounds 4a–v:

Alkyne **3** (0.30 g, 0.597 mmol, 1 equiv), the corresponding amine (0.716 mmol, 1.2 equiv), aqueous formaldehyde (35%, 1 mL), and CuI (20 mg) in 2 mL DMSO were stirred at room temperature for 3 h. Then, 10 mL of water were added, the mixture extracted with EtOAc (2 × 20 mL), dried and evaporated. The residue was purified by column chromatography (EtOAc/MeOH 10:1).

(3*R*,5*S*,7*R*,8*R*,9*S*,10*S*,12*S*,13*R*,14*S*,17*R*)-10,13-Dimethyl-17-((*R*)-4-(5-((4-(4-phenyl piperazin-1-yl)but-2-yn-1-yl)thio)-1,3,4-oxadiazol-2-yl)butan-2-yl)hexadecahydro-1*H*-cyclopenta[*a*]phenanthrene-3,7,12-triol (4a)

Colorless oil (0.313 g, 77% yield). ^{1}H NMR (500 MHz, $CDCl_3$): δ 0.63 (s, 3H, CH_3); 0.84 (s, 3H, CH_3); 0.99 (d, 3H, J= 6.0, CH_3); 1.30–1.95 (m, 20H, CH_2 and CH); 2.09-2.29 (m, 2H, CH); 2.71 (m, 4H, CH_2); 2.84 (m, 2H, CH_2); 3.21 (m, 4H, CH_2); 3.38 (m, 3H, CH_2 and CH); 3.80 (m, 1H, CH); 3.91 (m, 1H, CH); 3.99 (s, 2H, CH_2); 6.78-6.85 (m, 1H, CH-aromatic); 6.89-6.98 (m, 2H, CH-aromatic); 7.18-7.24 (m, 2H, CH-aromatic).^{13}C NMR (125 MHz, $CDCl_3$): δ 12.4, 17.2, 21.5, 22.3, 22.4, 23.2, 26.4, 27.5, 28.2, 30.4, 32.3, 34.6, 34.7, 35.2, 35.3, 39.4, 39.6, 41.4, 41.7, 46.4, 46.6, 47.1, 48.8, 51.8, 68.4, 71.8, 72.9, 116.2, 120.0, 129.1, 151.0, 162.3, 169.0. HRMS (ESI-MS): m/z calcd for $C_{39}H_{57}N_4O_4S$: 677.4095 $[M+H]^+$; found 677.4096.

(3*R*,5*S*,7*R*,8*R*,9*S*,10*S*,12*S*,13*R*,14*S*,17*R*)-17-((*R*)-4-(5-((4-(4-(4-Fluorophenyl) piperazin-1-yl)but-2-yn-1-yl)thio)-1,3,4-oxadiazol-2-yl)butan-2-yl)-10,13-dimethylhexadecahydro-1*H*-cyclopenta[*a*]phenanthrene-3,7,12-triol (4b)

Yellow oil (0.342 g, 83% yield). ^{1}H NMR (500 MHz, $CDCl_3$): δ 0.65 (s, 3H, CH_3); 0.87 (s, 3H, CH_3); 1.01 (d, 3H, J= 5.8, CH_3); 1.31–1.97 (m, 20H, CH_2 and CH); 2.10-2.29 (m, 2H, CH); 2.57-2.66 (m, 1H, CH); 2.67-2.82 (m, 4H, CH_2 and CH); 2.82-2.92 (m, 1H, CH); 3.08-3.25 (m, 4H, CH_2); 3.36-3.53 (m, 3H, CH_2 and CH); 3.83 (m, 1H, CH); 3.94 (m, 1H, CH); 4.02 (s, 2H, CH_2); 4.13-4.39 (br,3H, OH); 6.82-6.88 (m, 2H, CH-aromatic); 6.93-7.04 (m, 2H, CH-aromatic).^{13}C NMR (125 MHz, $CDCl_3$): δ 12.4, 17.2, 21.5, 22.3, 22.4, 23.2, 26.4, 27.5, 28.2, 30.4, 32.3, 34.6, 34.7, 35.2, 35.3, 39.4, 539.5, 41.4, 41.6, 46.4, 46.6, 47.1, 49.7, 51.6, 68.4, 71.8, 73.0, 115.5 (d, J C–F = 22.2 Hz), 118.1 (d, J C–F = 7.7 Hz), 147.6, 156.3, 164.3, 170.2. HRMS (ESI-MS): m/z calcd for $C_{39}H_{56}FN_4O_4S$: 695.4001 $[M+H]^+$; found 695.3976.

(3*R*,5*S*,7*R*,8*R*,9*S*,10*S*,12*S*,13*R*,14*S*,17*R*)-17-((*R*)-4-(5-((4-(4-(2-Fluorophenyl) piperazin-1-yl)but-2-yn-1-yl)thio)-1,3,4-oxadiazol-2-yl)butan-2-yl)-10,13-dimethyl hexadecahydro-1*H*-cyclopenta[*a*]phenanthrene-3,7,12-triol (4c)

Colorless oil (0.373 g, 90% yield). ^{1}H NMR (500 MHz, $CDCl_3$): δ 0.66 (s, 3H, CH_3); 0.87 (s, 3H, CH_3); 1.02 (d, 3H, J= 6.2, CH_3); 1.19–1.98 (m, 20H, CH_2 and CH); 2.15-2.24 (m, 2H, CH); 2.64-3.01 (m, 6H, CH_2 and CH); 3.09-3.28 (m, 4H, CH_2); 3.37-3.54 (m, 3H, CH_2 and CH); 3.83 (m, 1H, CH); 3.95 (m, 1H, CH); 4.04 (s, 2H, CH_2); 4.25-4.48 (br,3H, OH); 6.90-7.16 (m, 4H, CH-aromatic).^{13}C NMR (125 MHz, $CDCl_3$): δ 12.4, 17.2, 21.5, 22.3, 22.4, 23.2, 26.3, 27.5, 28.1, 30.3, 32.3, 34.6, 34.7, 35.2, 35.3, 39.4, 39.5, 41.4, 41.6, 46.4, 46.6, 47.1, 49.8, 51.6, 68.4, 71.9, 73.0, 116.1 (d, J C–F = 20.6 Hz), 119.0 (d, J C–F = 2.6 Hz), 122.7 (d, J C–F = 7.9 Hz); 124.5 (d, J C–F = 3.5 Hz); 139.6 (d, J C–F = 8.2 Hz); 155.6 (d, J C–F = 245.9 Hz), 162.4, 169.4. HRMS (ESI-MS): m/z calcd for $C_{39}H_{56}FN_4O_4S$: 695.4001 $[M+H]^+$; found 695.4020.

(3*R*,5*S*,7*R*,8*R*,9*S*,10*S*,12*S*,13*R*,14*S*,17*R*)-17-((*R*)-4-(5-((4-(4-(4-Methoxyphenyl) piperazin-1-yl)but-2-yn-1-yl)thio)-1,3,4-oxadiazol-2-yl)butan-2-yl)-10,13-dimethyl hexadecahydro-1*H*-cyclopenta[*a*]phenanthrene-3,7,12-triol (4d)

Yellow oil (0.407 g, 96% yield). ^{1}H NMR (500 MHz, $CDCl_3$): δ 0.65 (s, 3H, CH_3); 0.86 (s, 3H, CH_3); 1.00 (d, 3H, J= 5.6, CH_3); 1.31–1.99 (m, 20H, CH_2 and CH); 2.16-2.23 (m, 2H, CH); 2.68-2.96 (m, 6H, CH_2 and CH); 3.04-3.24 (m, 4H, CH_2); 3.36-3.53 (m, 3H, CH_2 and CH); 3.75 (s, 3H, OCH_3); 3.83 (m, 1H, CH); 3.94 (m, 1H, CH); 4.04 (s, 2H, CH_2); 4.40-4.59 (bs, 3H, OH); 6.79-6.86 (m, 2H, CH-aromatic); 6.86-6.94 (m, 2H, CH-aromatic).^{13}C NMR (125 MHz, $CDCl_3$): δ 12.4, 17.2, 21.5, 22.3, 22.4, 23.2, 26.3, 27.5, 28.1, 30.2, 32.3, 34.5, 34.7, 35.2, 35.2, 39.4, 39.4, 41.4, 41.6, 46.4, 46.6, 50.1, 50.1, 51.5, 55.5, 68.4, 71.8, 73.0, 114.4, 118.5, 154.1, 163.2, 172.6. HRMS (ESI-MS): m/z calcd for $C_{40}H_{59}N_4O_5S$: 707.4201 $[M+H]^+$; found 707.4180.

(3*R*,5*S*,7*R*,8*R*,9*S*,10*S*,12*S*,13*R*,14*S*,17*R*)-10,13-Dimethyl-17-((*R*)-4-(5-((4-(4-(pyridin-2-yl)piperazin-1-yl)but-2-yn-1-yl)thio)-1,3,4-oxadiazol-2-yl)butan-2-yl)hexadecahydro-1*H*-cyclopenta[*a*]phenanthrene-3,7,12-triol (4e)

Brown oil (0.296 g, 73% yield). ^{1}H NMR (500 MHz, $CDCl_3$): δ 0.65 (s, 3H, CH_3); 0.87 (s, 3H, CH_3); 1.01 (d, 3H, J= 6.2, CH_3); 1.32–2.05 (m, 20H, CH_2 and CH); 2.10-2.28 (m, 2H, CH); 2.65-2.70 (m, 4H, CH_2 and CH); 2.83-2.94 (m, 2H, CH_2); 3.31-3.42 (m, 3H, CH_2 and CH); 3.51-3.64 (m, 4H, CH_2); 3.82 (m, 1H, CH); 3.94 (m, 1H, CH); 4.00 (s, 2H, CH_2); 6.55-6.76 (dd, 2H, J= 17.0, 7.4, CH-aromatic); 7.48 (t, 1H, , J= 7.4, CH-aromatic); 8.17 (d, 1H, , J= 3.2, CH-aromatic).^{13}C NMR (125 MHz, $CDCl_3$): δ 12.4, 17.2, 21.5, 22.3, 22.4, 23.2, 26.4, 27.5, 28.2, 30.4, 32.4, 34.6, 34.7, 35.2, 35.3, 39.4, 39.6, 41.4, 41.7, 45.0, 46.4, 46.6, 47.2, 51.7, 68.4, 71.8, 72.9, 107.3, 113.5, 137.7, 147.7, 159.2, 162.5, 169.0. HRMS (ESI-MS): m/z calcd for $C_{38}H_{56}N_5O_4S$: 678.4048 $[M+H]^+$; found 678.4037.

1-(4-(4-(4-((5-((*R*)-3-((3*R*,5*S*,7*R*,8*R*,9*S*,10*S*,12*S*,13*R*,14*S*,17*R*)-3,7,12-Trihydroxy-10,13-dimethylhexadecahydro-1*H*-cyclopenta[*a*]phenanthren-17-yl)butyl)-1,3,4-oxadiazol-2-yl)thio)but-2-yn-1-yl)piperazin-1-yl)phenyl)ethan-1-one (4f)

Yellow oil (0.411 g, 96% yield). ^{1}H NMR (500 MHz, $CDCl_3$): δ 0.64 (s, 3H, CH_3); 0.85 (m, 3H, CH_3); 1.00 (d, 3H, J= 5.9, CH_3); 1.31–1.90 (m, 20H, CH_2 and CH); 2.10-2.24 (m, 2H, CH_2); 2.50 (s, 3H, CH_3); 2.69-2.85 (m, 6H, CH_2 and CH); 3.31-3.42 (m, 7H, CH_2 and CH); 3.81 (m, 1H, CH); 3.92 (m, 1H, CH); 3.99 (s, 2H, CH_2); 6.86 (d, 2H, J= 8.3, CH-aromatic); 7.86 (d, 2H, J= 8.3, CH-aromatic).^{13}C NMR (125 MHz, $CDCl_3$): δ 12.4, 17.2, 21.4, 22.2, 22.4, 23.1, 26.1, 26.3, 27.5, 28.1, 30.3, 32.3, 34.6, 34.7, 35.2, 35.2, 39.4, 39.5, 40.8, 41.4, 41.6, 46.4, 46.5, 46.9, 51.2, 68.3, 71.8, 72.9, 113.6, 127.8, 130.4, 153.9, 163.3, 169.2, 196.7. HRMS (ESI-MS): m/z calcd for $C_{41}H_{59}N_4O_4S$: 719.4201 $[M+H]^+$; found 719.4241.

(3*R*,5*S*,7*R*,8*R*,9*S*,10*S*,12*S*,13*R*,14*S*,17*R*)-17-((*R*)-4-(5-((4-(4-(2-Hydroxyethyl) piperazin-1-yl)but-2-yn-1-yl)thio)-1,3,4-oxadiazol-2-yl)butan-2-yl)-10,13-dimethyl hexadecahydro-1*H*-cyclopenta[*a*]phenanthrene-3,7,12-triol (4g)

Yellow oil (0.307 g, 79% yield). ^{1}H NMR (500 MHz, $CDCl_3$): δ 0.66 (s, 3H, CH_3); 0.87 (s, 3H, CH_3); 1.03 (d, 3H, J= 6.4, CH_3); 1.32–1.97 (m, 20H, CH_2 and CH); 2.11-2.27 (m,

2H, CH_2); 2.44-2.73 (m, 10H, CH_2 and CH); 2.73–2.79 (m, 1H, CH); 2.85–2.93 (m, 1H, CH); 3.28 (m, 2H, CH_2); 3.36-3.45 (m, 1H, CH); 3.64-3.72 (m, 2H, CH); 3.82 (m, 1H, CH); 3.94 (m, 1H, CH); 3.97 (s, 2H, CH_2). ^{13}C NMR (125 MHz, $CDCl_3$): δ 12.4, 17.2, 21.6, 22.2, 22.4, 23.2, 26.4, 27.6, 28.2, 30.3, 32.4, 34.6, 34.7, 35.3, 39.4, 39.6, 41.5, 41.7, 46.4, 46.5, 46.9, 51.3, 52.7, 57.7, 58.3, 59.3, 68.3, 71.8, 72.9, 79.0, 79.6, 162.4, 169.2. HRMS (ESI-MS): m/z calcd for $C_{35}H_{57}N_4O_5S$: 645.4044 $[M+H]^+$; found 645.4053.

4-(4-((5-((*R*)-3-((3*R*,5*S*,7*R*,8*R*,9*S*,10*S*,12*S*,13*R*,14*S*,17*R*)-3,7,12-Trihydroxy-10,13-dimethylhexadecahydro-1*H*-cyclopenta[*a*]phenanthren-17-yl)butyl)-1,3,4-oxadiazol-2-yl)thio)but-2-yn-1-yl)piperazine-1-carbaldehyde (4h)

Yellow oil (0.352 g, 94% yield). ^1H NMR (500 MHz, $CDCl_3$): δ 0.66 (s, 3H, CH_3); 0.86 (m, 3H, CH_3); 1.03 (d, 3H, J= 5.8, CH_3); 1.31–1.96 (m, 20H, CH_2 and CH); 2.07-2.26 (m, 2H, CH_2); 2.38-2.60 (m, 4H, CH_2 and CH); 2.68-2.78 (m, 1H, CH); 2.85-2.90 (m, 1H, CH); 3.29-3.47 (m, 5H, CH_2 and CH); 3.58 (s, 2H, CH_2); 3.82 (m, 1H, CH); 3.94 (m, 1H, CH); 3.98 (s, 2H, CH_2); 8.01 (s, 1H, CHO). ^{13}C NMR (125 MHz, $CDCl_3$): δ 12.4, 17.2, 21.4, 22.2, 22.4, 23.2, 26.3, 27.5, 28.1, 30.3, 32.4, 34.6, 34.7, 35.3, 35.3, 39.4, 39.5, 41.4, 41.6, 45.2, 46.4, 46.5, 47.1, 51.0, 68.3, 71.8, 72.9, 160.8, 162.4, 169.1. HRMS (ESI-MS): m/z calcd for $C_{34}H_{52}N_4NaO_5S$: 651.3551 $[M+Na]^+$; found 651.3562.

(3*R*,5*S*,7*R*,8*R*,9*S*,10*S*,12*S*,13*R*,14*S*,17*R*)-17-((*R*)-4-(5-((4-(4-Methylpiperazin-1-yl)but-2-yn-1-yl)thio)-1,3,4-oxadiazol-2-yl)butan-2-yl)-10,13-dimethylhexa decahydro-1*H*-cyclopenta[*a*]phenanthrene-3,7,12-triol (4i)

White solid (0.353 g, 96% yield), m.p: 82-83 °C. ^1H NMR (500 MHz, $CDCl_3$): δ 0.66 (s, 3H, CH_3); 0.87 (m, 3H, CH_3); 1.03 (d, 3H, J= 6.4, CH_3); 1.33–1.98 (m, 20H, CH_2 and CH); 2.08-2.23 (m, 2H, CH_2); 2.34 (s, 3H, NCH_3); 2.60 (m, 4H, CH_2 and CH); 2.72-2.79 (m, 1H, CH); 2.85-2.92 (m, 1H, CH); 2.93-3.18 (m, 4H, CH_2); 3.33 (s, 2H, CH_2); 3.35-3.47 (m, 1H, CH); 3.82 (m, 1H, CH); 3.95 (m, 1H, CH); 3.99 (s, 2H, CH_2). ^{13}C NMR (125 MHz, $CDCl_3$): δ 12.4, 17.3, 21.5, 22.3, 22.4, 23.2, 26.4, 27.5, 28.2, 30.4, 32.3, 34.7, 34.7, 35.2, 35.3, 39.4, 39.6, 41.5, 41.7, 45.6, 46.4, 46.6, 47.0, 51.4, 54.7, 68.3, 71.8, 72.9, 78.7, 79.8, 162.5, 169.0. HRMS (ESI-MS): m/z calcd for $C_{34}H_{55}N_4O_4S$: 615.3939 $[M+H]^+$; found 615.3957.

(3*R*,5*S*,7*R*,8*R*,9*S*,10*S*,12*S*,13*R*,14*S*,17*R*)-17-((*R*)-4-(5-((4-(4-Ethylpiperazin-1-yl)but-2-yn-1-yl)thio)-1,3,4-oxadiazol-2-yl)butan-2-yl)-10,13-dimethylhexadecahydro-1*H*-cyclopenta[*a*]phenanthrene-3,7,12-triol (4j)

Yellow oil (0.324 g, 86% yield). ^{1}H NMR (500 MHz, $CDCl_3$): δ 0.66 (s, 3H, CH_3); 0.87 (m, 3H, CH_3); 1.03 (d, 3H, J= 6.4, CH_3); 1.10 (t, 3H, J= 7.2, CH_3); 1.32–1.97 (m, 20H, CH_2 and CH); 2.13-2.25 (m, 2H, CH_2); 2.47 (q, 2H, J= 7.2, NCH_2); 2.60 (m, 4H, CH_2 and CH); 2.69–2.78 (m, 1H, CH); 2.82–2.91 (m, 1H, CH); 2.91–3.14 (m, 4H, CH_2); 3.28 (s, 2H, CH_2); 3.36-3.44 (m, 1H, CH); 3.82 (m, 1H, CH); 3.94 (m, 1H, CH); 3.99 (s, 2H, CH_2). ^{13}C NMR (125 MHz, $CDCl_3$): δ 11.6, 12.4, 17.2, 21.5, 22.3, 22.4, 23.2, 26.3, 27.5, 28.2, 30.4, 32.4, 34.7, 34.7, 35.2, 35.3, 39.4, 39.6, 41.5, 41.6, 46.4, 46.6, 47.0, 51.6, 52.1, 52.3, 68.3, 71.8, 72.9, 78.6, 79.9, 162.6, 169.0. HRMS (ESI-MS): m/z calcd for $C_{35}H_{57}N_4O_4S$: 629.4095 $[M+H]^+$; found 629.4121.

***N*-Isopropyl-2-(4-(4-((5-((*R*)-3-((3*R*,5*S*,7*R*,8*R*,9*S*,10*S*,12*S*,13*R*,14*S*,17*R*)-3,7,12-trihydroxy-10,13-dimethylhexadecahydro-1*H*-cyclopenta[*a*]phenanthren-17-yl) butyl)-1,3,4-oxadiazol-2-yl)thio)but-2-yn-1-yl)piperazin-1-yl)acetamide (4k)**

Yellow oil (0.217 g, 52% yield). ^{1}H NMR (500 MHz, $CDCl_3$): δ 0.64 (s, 3H, CH_3); 0.84 (s, 3H, CH_3); 1.01 (d, 3H, J= 6.4, CH_3); 1.15 (d, 6H, J= 6.6, CH_3); 1.28–1.90 (m, 20H, CH_2 and CH); 2.07-2.25 (m, 2H, CH_2); 2.46-2.55 (m, 6H, CH_2 and CH); 2.66–2.76 (m, 2H, CH_2); 2.77–2.90 (m, 2H, CH_2); 2.99 (s, 2H, CH_2); 3.28 (s, 2H, CH_2); 3.35-3.41 (m, 1H, CH); 3.74-3.83 (m, 1H, CH); 3.86-3.94 (m, 1H, CH); 3.98 (s, 2H, CH_2); 4.05 (sep, 1H, CH); 7.08 (d, 1H, J= 7.7, NH). ^{13}C NMR (125 MHz, $CDCl_3$): δ 12.4, 17.2, 21.5, 22.2, 22.3, 22.6, 22.7, 23.1, 26.2, 27.5, 28.1, 30.2, 32.4, 34.6, 34.7, 35.2, 35.3, 39.4, 39.5, 41.4, 41.5, 46.3, 46.6, 46.8, 51.6, 53.0, 61.2, 68.2, 71.7, 72.9, 79.0, 79.3, 162.3, 168.9, 169.1. HRMS (ESI-MS): m/z calcd for $C_{38}H_{62}N_5O_5S$: 700.4466 $[M+H]^+$; found 700.4487.

(3*R*,5*S*,7*R*,8*R*,9*S*,10*S*,12*S*,13*R*,14*S*,17*R*)-10,13-Dimethyl-17-((*R*)-4-(5-((4-thiomor pholinobut-2-yn-1-yl)thio)-1,3,4-oxadiazol-2-yl)butan-2-yl)hexadecahydro-1*H*-cyclopenta[*a*]phenanthrene-3,7,12-triol (4l)

Brown oil (0.284 g, 77% yield). ^{1}H NMR (500 MHz, $CDCl_3$): δ 0.66 (s, 3H, CH_3); 0.86 (m, 3H, CH_3); 1.02 (d, 3H, J= 6.3, CH_3); 1.31–1.92 (m, 20H, CH_2 and CH); 2.12-2.24 (m,

2H, CH_2); 2.70 (m, 4H, CH_2); 2.75 (m, 4H, CH_2); 3.24-3.31 (m, 3H, CH_2 and CH); 3.37-3.46 (m, 2H, CH_2); 3.75-3.82 (m, 1H, CH); 3.90-3.94 (m, 1H, CH); 4.00 (s, 2H, CH_2). ^{13}C NMR (125 MHz, $CDCl_3$): δ 12.4, 17.2, 21.5, 22.3, 22.4, 23.2, 26.3, 27.5, 27.9, 28.2, 30.3, 32.3, 34.6, 34.7, 35.2, 35.2, 39.4, 39.6, 41.4, 41.7, 46.4, 46.6, 48.3, 53.7, 68.3, 71.8, 72.9, 79.0, 79.5, 162.5, 169.0. HRMS (ESI-MS): m/z calcd for $C_{33}H_{52}N_3O_5S_2$: 618.3394 $[M+H]^+$; found 618.3386.

(3*R*,5*S*,7*R*,8*R*,9*S*,10*S*,12*S*,13*R*,14*S*,17*R*)-10,13-Dimethyl-17-((*R*)-4-(5-((4-morpholino but-2-yn-1-yl)thio)-1,3,4-oxadiazol-2-yl)butan-2-yl)hexadecahydro-1*H*-cyclopenta [*a*]phenanthrene-3,7,12-triol (4m)

Yellow oil (0.346 g, 96% yield). ^1H NMR (500 MHz, $CDCl_3$): δ 0.65 (s, 3H, CH_3); 0.86 (m, 3H, CH_3); 1.02 (d, 3H, J= 6.1, CH_3); 1.35–1.91 (m, 20H, CH_2 and CH); 2.15-2.22 (m, 2H, CH_2); 2.53-2.57 (m, 4H, CH_2); 2.71-2.78 (m, 1H, CH); 2.80-2.94 (m, 1H, CH); 3.31 (s, 2H, CH_2); 3.38-3.43 (m, 1H, CH); 3.72-3.74 (m, 4H, CH_2); 3.82 (m, 1H, CH); 3.94 (m, 1H, CH); 4.00 (s, 2H, CH_2). ^{13}C NMR (125 MHz, $CDCl_3$): δ 12.4, 17.2, 21.4, 22.3, 22.4, 23.2, 26.3, 27.5, 28.1, 30.2, 32.3, 34.6, 34.7, 35.2, 35.3, 39.4, 39.5, 41.4, 41.6, 46.4, 46.5, 47.2, 51.9, 66.4, 68.4, 71.8, 72.9, 78.7, 79.5, 162.4, 169.1. HRMS (ESI-MS): m/z calcd for $C_{33}H_{52}N_3O_5S$: 602.3622 $[M+H]^+$; found 602.3626.

(3*R*,5*S*,7*R*,8*R*,9*S*,10*S*,12*S*,13*R*,14*S*,17*R*)-17-((*R*)-4-(5-((4-(Benzyl(methyl)amino)but-2-yn-1-yl)thio)-1,3,4-oxadiazol-2-yl)butan-2-yl)-10,13-dimethylhexadecahydro-1*H*-cyclopenta[*a*]phenanthrene-3,7,12-triol (4n)

Colorless oil (0.311 g, 82% yield). ^1H NMR (500 MHz, $CDCl_3$): δ 0.63 (s, 3H, CH_3); 0.84 (s, 3H, CH_3); 1.00 (d, 3H, J= 5.2, CH_3); 1.30–1.95 (m, 20H, CH_2 and CH); 2.10-2.22 (m, 2H, CH_2); 2.22-2.30 (m, 3H, NCH_3); 2.72 (m, 1H, CH); 2.85 (m, 1H, CH); 3.19-3.40 (m, 3H, CH_2 and CH); 3.40-3.54 (s, 2H, CH_2); 3.70-3.87 (m, 1H, CH); 3.87-3.96 (m, 1H, CH); 4.02 (s, 2H, CH_2); 7.06-7.63 (m, 5H, CH-aromatic).^{13}C NMR (125 MHz, $CDCl_3$): δ 12.4, 17.2, 21.6, 22.3, 22.4, 23.2, 26.3, 27.5, 28.2, 30.4, 32.3, 34.7, 35.2, 35.3, 39.4, 39.6, 41.4, 41.6, 44.9, 46.4, 46.6, 47.2, 51.7, 59.8, 68.4, 71.8, 72.9, 127.4, 128.4, 129.3, 141.2, 162.6, 169.2. HRMS (ESI-MS): m/z calcd for $C_{37}H_{54}N_3O_4S$: 636.3830 $[M+H]^+$; found 636.3894.

(3*R*,5*S*,7*R*,8*R*,9*S*,10*S*,12*S*,13*R*,14*S*,17*R*)-10,13-Dimethyl-17-((*R*)-4-(5-((4-(piperidin-1-yl)but-2-yn-1-yl)thio)-1,3,4-oxadiazol-2-yl)butan-2-yl)hexadecahydro-1*H*-cyclo penta[*a*]phenanthrene-3,7,12-triol (4o)

Yellow oil (0.338 g, 94% yield). ^{1}H NMR (500 MHz, $CDCl_3$): δ 0.65 (s, 3H, CH_3); 0.86 (m, 3H, CH_3); 1.02 (d, 3H, J= 6.0, CH_3); 1.31–1.80 (m, 20H, CH_2 and CH); 1.80-1.89 (m, 4H, CH_2); 2.00-2.05 (m, 2H, CH_2); 2.12-2.28 (m, 2H, CH_2); 2.47-2.66 (m, 6H, CH_2 and CH); 2.70-2.78 (m, 1H, CH); 2.81-2.95 (m, 1H, CH); 3.28-3.52 (m, 3H, CH_2 and CH); 3.82-3.94 (m, 4H, CH_2 and CH); 4.00 (s, 2H, CH_2). ^{13}C NMR (125 MHz, $CDCl_3$): δ 12.4, 17.2, 21.5, 22.3, 22.4, 23.2, 23.2, 25.0, 26.3, 27.5, 28.1, 30.3, 32.3, 34.6, 34.7, 35.2, 35.3, 39.4, 39.5, 41.4, 41.6, 46.4, 46.6, 47.2, 52.6, 68.4, 71.8, 72.9, 78.8, 79.5, 162.5, 169.1. HRMS (ESI-MS): m/z calcd for $C_{34}H_{54}N_3O_5S$: 600.3830 $[M+H]^+$; found 600.3851.

(3*R*,5*S*,7*R*,8*R*,9*S*,10*S*,12*S*,13*R*,14*S*,17*R*)-10,13-Dimethyl-17-((*R*)-4-(5-((4-(pyrrolidin-1-yl)but-2-yn-1-yl)thio)-1,3,4-oxadiazol-2-yl)butan-2-yl)hexadecahydro-1*H*-cyclo penta[*a*]phenanthrene-3,7,12-triol (4p)

Yellow oil (0.331 g, 95% yield). ^{1}H NMR (500 MHz, $CDCl_3$): δ 0.65 (s, 3H, CH_3); 0.86 (m, 3H, CH_3); 1.02 (d, 3H, J= 5.9, CH_3); 1.35–1.98 (m, 24H, CH_2 and CH); 2.11-2.33 (m, 2H, CH_2); 2.65-2.97 (m, 6H, CH_2 and CH); 3.31-3.47 (m, 1H, CH); 3.51 (s, 2H, CH_2); 3.75-3.87 (m, 1H, CH); 3.87-3.94 (m, 1H, CH); 3.99 (s, 2H, CH_2); 4.61 (br, 3H, OH). ^{13}C NMR (125 MHz, $CDCl_3$): δ 12.4, 17.2, 21.4, 22.3, 22.4, 23.2, 23.8, 26.3, 27.5, 28.1, 30.3, 32.4, 34.6, 34.7, 35.3, 35.3, 39.4, 39.5, 41.4, 41.6, 42.4, 46.4, 46.6, 51.6, 68.3, 71.8, 72.9, 78.3, 79.6, 162.4, 169.1. HRMS (ESI-MS): m/z calcd for $C_{33}H_{52}N_3O_4S$: 586.3673 $[M+H]^+$; found 586.3670.

(3*R*,5*S*,7*R*,8*R*,9*S*,10*S*,12*S*,13*R*,14*S*,17*R*)-17-((*R*)-4-(5-((4-(Diisopropylamino)but-2-yn-1-yl)thio)-1,3,4-oxadiazol-2-yl)butan-2-yl)-10,13-dimethylhexadecahydro-1*H*-cyclopenta[*a*]phenanthrene-3,7,12-triol (4q)

Yellow oil (0.350 g, 95% yield). ^{1}H NMR (500 MHz, $CDCl_3$): δ 0.62 (s, 3H, CH_3); 0.83 (s, 3H, CH_3); 0.99 (d, 3H, J= 6.4, CH_3); 1.10 (d, 12H, J= 6.4, CH_3); 1.30–1.92 (m, 20H, CH_2 and CH); 2.08-2.22 (m, 2H, CH_2); 2.63–2.74 (m, 1H, CH); 2.74–2.89 (m, 1H, CH); 3.24 (sep, 2H, CH); 3.30-3.41 (m, 1H, CH); 3.50 (s, 2H, CH_2); 3.79 (m, 1H, CH); 3.91 (m, 1H,

CH); 3.94 (s, 2H, CH_2). ^{13}C NMR (125 MHz, $CDCl_3$): δ 12.4, 17.2, 19.8, 21.7, 22.3, 22.4, 23.2, 26.3, 27.5, 28.1, 30.3, 32.4, 34.2, 34.6, 34.7, 35.2, 35.3, 39.4, 39.5, 41.4, 41.6, 46.4, 46.6, 49.4, 68.4, 71.8, 72.9, 162.5, 169.0. HRMS (ESI-MS): m/z calcd for $C_{35}H_{58}N_3O_4S$: 616.4142 $[M+H]^+$; found 616.4146.

(3*R*,5*S*,7*R*,8*R*,9*S*,10*S*,12*S*,13*R*,14*S*,17*R*)-17-((*R*)-4-(5-((4-(Diethylamino)but-2-yn-1-yl)thio)-1,3,4-oxadiazol-2-yl)butan-2-yl)-10,13-dimethylhexadecahydro-1*H*-cyclo penta[*a*]phenanthrene-3,7,12-triol (4r)

Yellow oil (0.288 g, 82% yield). ^{1}H NMR (500 MHz, $CDCl_3$): δ 0.66 (s, 3H, CH_3); 0.87 (m, 3H, CH_3); 1.02-1.14 (m, 9H, CH_3); 1.30–1.98 (m, 20H, CH_2 and CH); 2.07-2.29 (m, 2H, CH_2); 2.47-2.56 (q, 4H, CH_2); 2.69-2.78 (m, 1H, CH); 2.81-t2.91 (m, 1H, CH); 3.35-3.54 (m, 3H, CH_2 and CH); 3.82 (m, 1H, CH); 3.94 (m, 1H, CH); 3.99 (s, 2H, CH_2). ^{13}C NMR (125 MHz, $CDCl_3$): δ 12.2, 12.4, 17.2, 21.6, 22.3, 22.4, 23.2, 26.3, 27.5, 28.1, 30.3, 32.4, 34.7, 34.7, 35.2, 35.3, 39.4, 39.5, 40.7, 41.4, 41.6, 46.4, 46.6, 47.2, 68.4, 71.8, 72.9, 78.6, 79.1, 162.5, 169.0. HRMS (ESI-MS): m/z calcd for $C_{33}H_{54}N_3O_4S$: 588.3830 $[M+H]^+$; found 588.3854.

(3*R*,5*S*,7*R*,8*R*,9*S*,10*S*,12*S*,13*R*,14*S*,17*R*)-17-((*R*)-4-(5-((4-(Dipropylamino)but-2-yn-1-yl)thio)-1,3,4-oxadiazol-2-yl)butan-2-yl)-10,13-dimethylhexadecahydro-1*H*-cyclo penta[*a*]phenanthrene-3,7,12-triol (4s)

Yellow oil (0.337 g, 91% yield). ^{1}H NMR (500 MHz, $CDCl_3$): δ 0.68 (s, 3H, CH_3); 0.84-.93 (m, 9H, CH_3); 1.06 (d, 3H, J= 6.4, CH_3); 1.39–1.94 (m, 24H, CH_2 and CH); 2.14-2.26 (m, 2H, CH_2); 2.33-2.51 (m, 4H, CH_2); 2.70–2.80 (m, 1H, CH); 2.83–2.96 (m, 1H, CH); 3.37-3.47 (m, 3H, CH_2 and CH); 3.85 (m, 1H, CH); 3.97 (m, 1H, CH); 4.03 (s, 2H, CH_2). ^{13}C NMR (125 MHz, $CDCl_3$): δ 11.8, 12.4, 17.2, 20.4, 21.6, 22.3, 22.4, 23.2, 26.3, 27.5, 28.2, 30.4, 32.4, 34.7, 34.7, 35.2, 35.3, 39.4, 39.5, 41.4, 41.6, 42.1, 46.4, 46.6, 55.6, 68.4, 71.8, 72.9, 78.4, 79.7, 162.6, 169.0. HRMS (ESI-MS): m/z calcd for $C_{35}H_{58}N_3O_4S$: 616.4143 $[M+H]^+$; found 616.4119.

(3*R*,5*S*,7*R*,8*R*,9*S*,10*S*,12*S*,13*R*,14*S*,17*R*)-17-((*R*)-4-(5-((4-(Dibutylamino)but-2-yn-1-yl)thio)-1,3,4-oxadiazol-2-yl)butan-2-yl)-10,13-dimethylhexadecahydro-1*H*-cyclo penta[*a*]phenanthrene-3,7,12-triol (4t)

Yellow oil (0.366 g, 95% yield). ^{1}H NMR (500 MHz, $CDCl_3$): δ 0.67 (s, 3H, CH_3); 0.84-.93 (m, 9H, CH_3); 1.04 (d, 3H, J= 6.4, CH_3); 1.22–1.89 (m, 28H, CH_2 and CH); 2.14-2.26 (m, 2H, CH_2); 2.39-2.52 (m, 4H, CH_2); 2.79-2.85 (m, 1H, CH); 2.85–2.90 (m, 1H, CH); 3.38-3.50 (m, 3H, CH_2 and CH); 3.84 (m, 1H, CH); 3.96 (m, 1H, CH); 4.01 (s, 2H, CH_2). ^{13}C NMR (125 MHz, $CDCl_3$): δ 12.5, 14.0, 17.3, 20.6, 21.6, 22.3, 22.4, 23.2, 26.4, 27.5, 28.2, 29.2, 30.4, 32.4, 34.6, 34.7, 35.2, 35.2, 39.5, 39.6, 41.4, 41.7, 42.0, 46.4, 46.7, 53.4, 68.4, 71.9, 72.9, 162.6, 168.9. HRMS (ESI-MS): m/z calcd for $C_{37}H_{62}N_3O_4S$: 644.4456 $[M+H]^+$; found 644.4476.

(3*R*,5*S*,7*R*,8*R*,9*S*,10*S*,12*S*,13*R*,14*S*,17*R*)-17-((*R*)-4-(5-((4-(Dihexylamino)but-2-yn-1-yl)thio)-1,3,4-oxadiazol-2-yl)butan-2-yl)-10,13-dimethylhexadecahydro-1*H*-cyclo penta[*a*]phenanthrene-3,7,12-triol (4u)

Yellow oil (0.365 g, 88% yield). ^{1}H NMR (500 MHz, $CDCl_3$): δ 0.66 (s, 3H, CH_3); 0.79-0.92 (m, 9H, CH_3); 1.02 (m, 3H, CH_3); 1.17–1.95 (m, 36H, CH_2 and CH); 2.13-2.21 (m, 2H, CH_2); 2.43-2.55 (m, 4H, CH_2); 2.67-2.76 (m, 1H, CH); 2.81-2.89 (m, 1H, CH); 3.32-3.58 (m, 3H, CH_2 and CH); 3.82 (m, 1H, CH); 3.94 (m, 1H, CH); 4.01 (s, 2H, CH_2); 4.07-4.02 (bs, 3H, OH).^{13}C NMR (125 MHz, $CDCl_3$): δ 12.4, 14.0, 17.2, 21.5, 22.3, 22.4, 22.5, 23.2, 26.3, 26.5, 27.0, 27.5, 28.1, 30.3, 31.6, 32.4, 34.6, 34.7, 35.2, 35.2, 39.4, 39.5, 41.4, 41.6, 41.7, 46.4, 46.6, 53.5, 68.4, 71.8, 73.0, 78.5, 79.2, 162.5, 169.0. HRMS (ESI-MS): m/z calcd for $C_{41}H_{70}N_3O_4S$: 700.5082 $[M+H]^+$; found 700.5097.

(3*R*,5*S*,7*R*,8*R*,9*S*,10*S*,12*S*,13*R*,14*S*,17*R*)-17-((*R*)-4-(5-((4-(Dicyclohexylamino)but-2-yn-1-yl)thio)-1,3,4-oxadiazol-2-yl)butan-2-yl)-10,13-dimethylhexadecahydro-1*H*-cyclopenta[*a*]phenanthrene-3,7,12-triol (4v)

Brown oil (0.382 g, 92% yield). ^{1}H NMR (500 MHz, $CDCl_3$): δ 0.64 (s, 3H, CH_3); 0.85 (s, 3H, CH_3); 1.01 (d, 3H, J= 6.4, CH_3); 1.15–1.94 (m, 40H, CH_2 and CH); 2.06-2.27 (m, 2H, CH_2); 2.72-2.77 (m, 2H, CH); 2.82-2.86 (m, 2H, CH); 3.31–3.43 (m, 1H, CH); 3.50 (s, 2H, CH_2); 3.80 (m, 1H, CH); 3.92 (m, 1H, CH); 3.96 (s, 2H, CH_2). ^{13}C NMR (125

MHz, $CDCl_3$): δ 12.4, 17.2, 21.7, 22.2, 22.3, 23.1, 25.9, 25.9, 26.2, 27.4, 28.0, 30.2, 30.6, 32.3, 34.6, 34.7, 35.1, 35.2, 35.3, 39.4, 39.5, 41.4, 41.5, 46.3, 46.5, 57.7, 68.3, 71.7, 72.9, 162.6, 168.9. HRMS (ESI-MS): m/z calcd for $C_{41}H_{66}N_3O_4S$: 696.4769 $[M+H]^+$; found 696.4781.

2. Antimicrobial activity assay

The antimicrobial activity of the newly synthesized compounds was investigated in order to determine the activity of these derivatives towards test microorganisms. All microbial strains were provided from culture collection of the Regional Center for Mycology and Biotechnology (RCMB), Al-Azhar University, Cairo, Egypt.

In a similar manner to a procedure from [1], the antimicrobial profile was tested against Gram-positive bacterial species (*Staphylococcus aureus* and *Bacillus subtilis*), Gram-negative bacterial species (*Escherichia coli, Proteus vulgaris*), as well as against fungi including one filamentous fungus (*Aspergillus fumigatus*) and one yeast species (*Candida albicans*) using a well diffusion method [2]. Briefly, 100 µL of the test bacteria/fungi were grown in 10 mL of fresh media until they reached a count of approximately 10^8 cells/mL for bacteria or 10^5 cells/mL for fungi. One hundred µL of the microbial suspension was spread onto agar plates corresponding to the broth in which they were maintained and tested for susceptibility by the well diffusion method. One hundred µL of each sample (at 10 mg/mL) was added to each well (6 mm diameter holes cut in the agar gel). The plates were incubated for 24 h at 37 °C (for bacteria and yeast) and for 48 h at 28 °C (for filamentous fungi). After incubation, the microorganisms' growth was evaluated. The resulting inhibition zone diameters were measured in millimeters and used as the criterion for the antimicrobial activity. If an organism was placed on the agar it would have not grown in the area around the well if it was susceptible to the chemical. This area of no growth around the well is known as a zone of inhibition and the size of this zone is proportional to the inhibitory action of the compound under investigation. Solvent controls (DMSO) were included in every experiment as negative control and showed no inhibition zones, confirming that it had no influence on growth of the tested microorganisms.

Positive controls were also performed using gentamycin as broad spectrum standard antibacterial drugs and ketoconazole as broad spectrum standard antifungal drug. For all biologically active samples MIC values were determined by the broth mirodilution method. Briefly, the microbes were grown on Mueller Hinton broth media on sterile microtiter plates and inoculated with the tested microorganism. Then, the plates were

treated with the tested compound (at 10 mg/mL) followed by a serial two-fold dilution to test a range of concentrations from 10000 to 1 µg/mL). After the end of incubation, the lowest concentration showing complete inhibition of growth was recorded as the MIC of the respective sample [2]. The results reported are means of at least three separate experiments each with three replicates.

3. NMR spectra

Compound 2

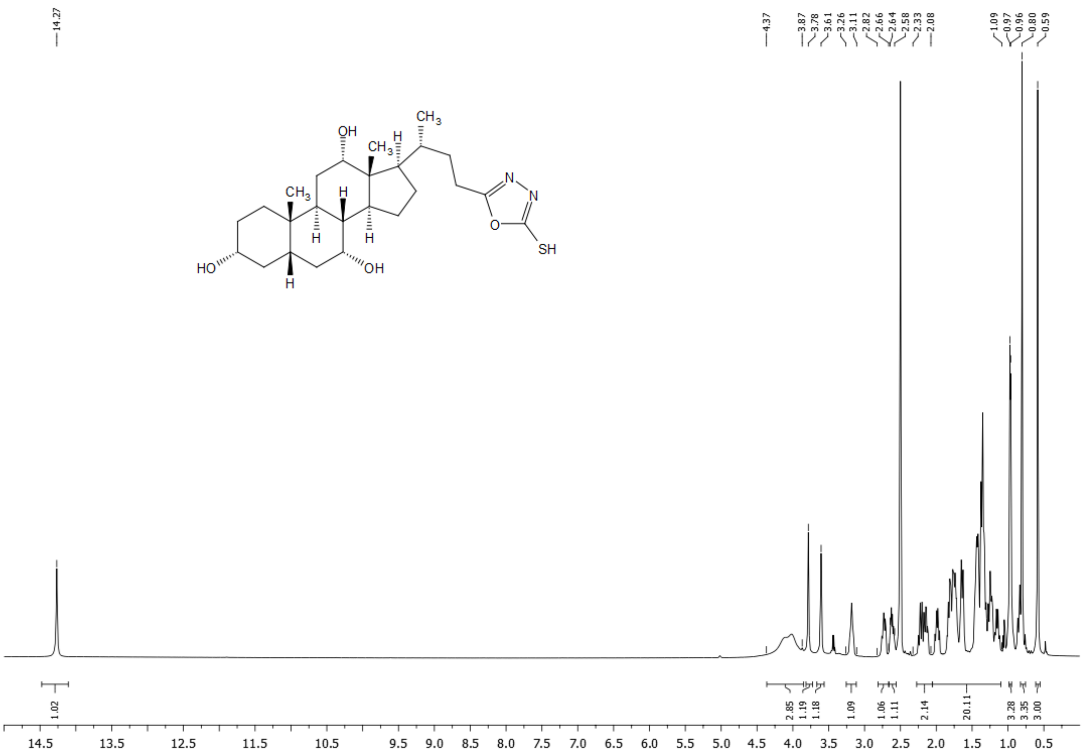

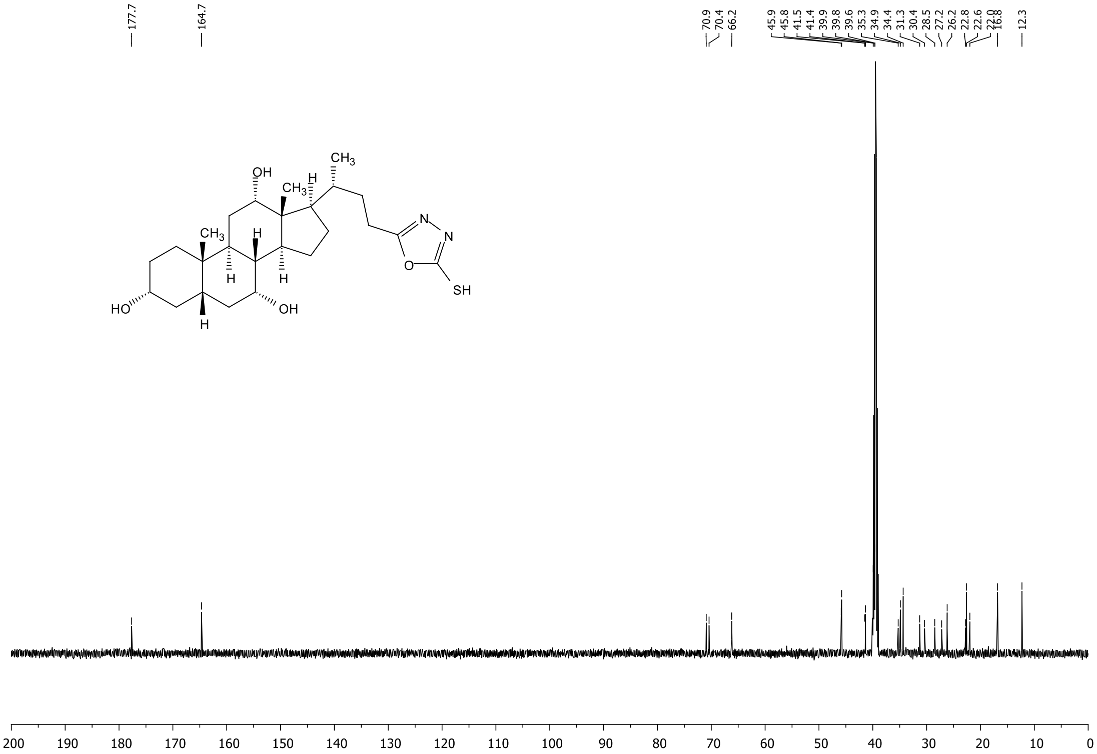

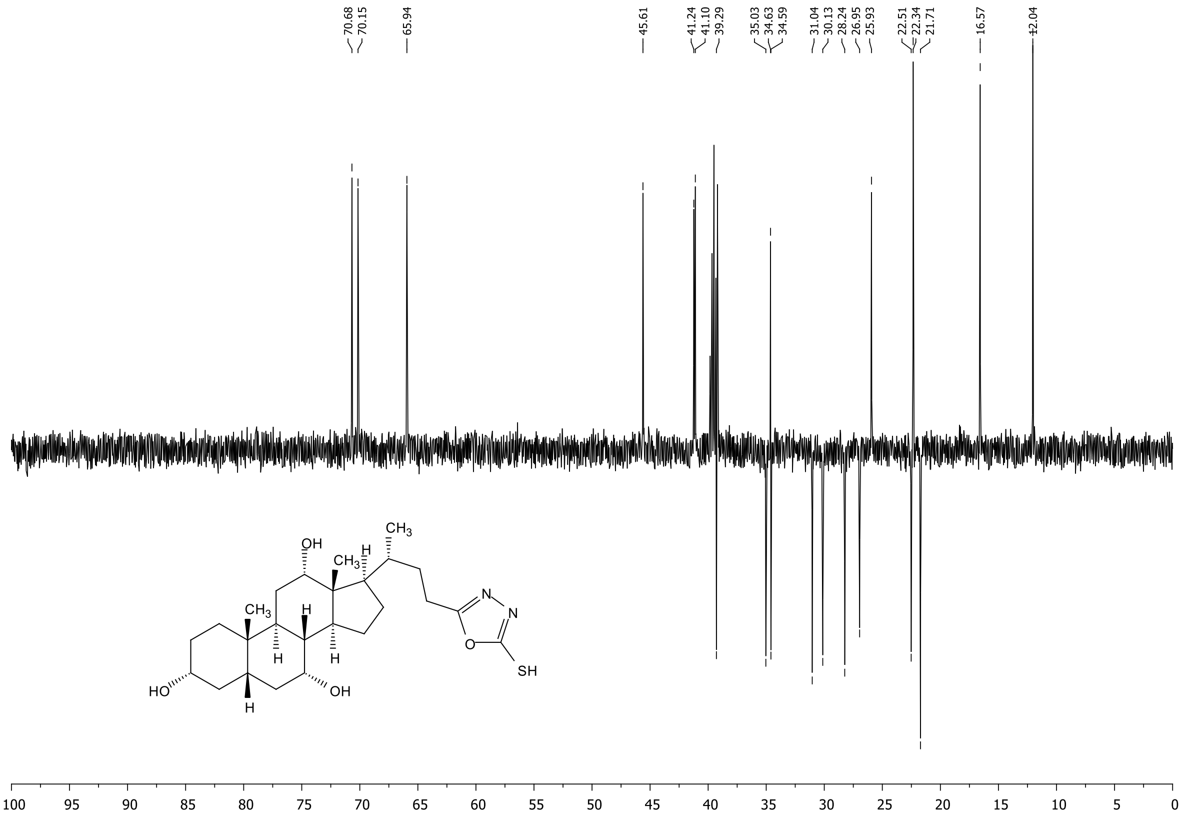
70.68
70.15
65.94
45.61
41.24
41.10
39.29
35.03
34.63
34.59
31.04
30.13
28.24
26.95
25.93
22.51
22.34
21.71
16.57
12.04
OH
CH3
H
CH3
HO
H
OH
N
N
O
SH
100
95
90
85
80
75
70
65
60
55
50
45
40
35
30
25
20
15
10
5
0

Compound 3

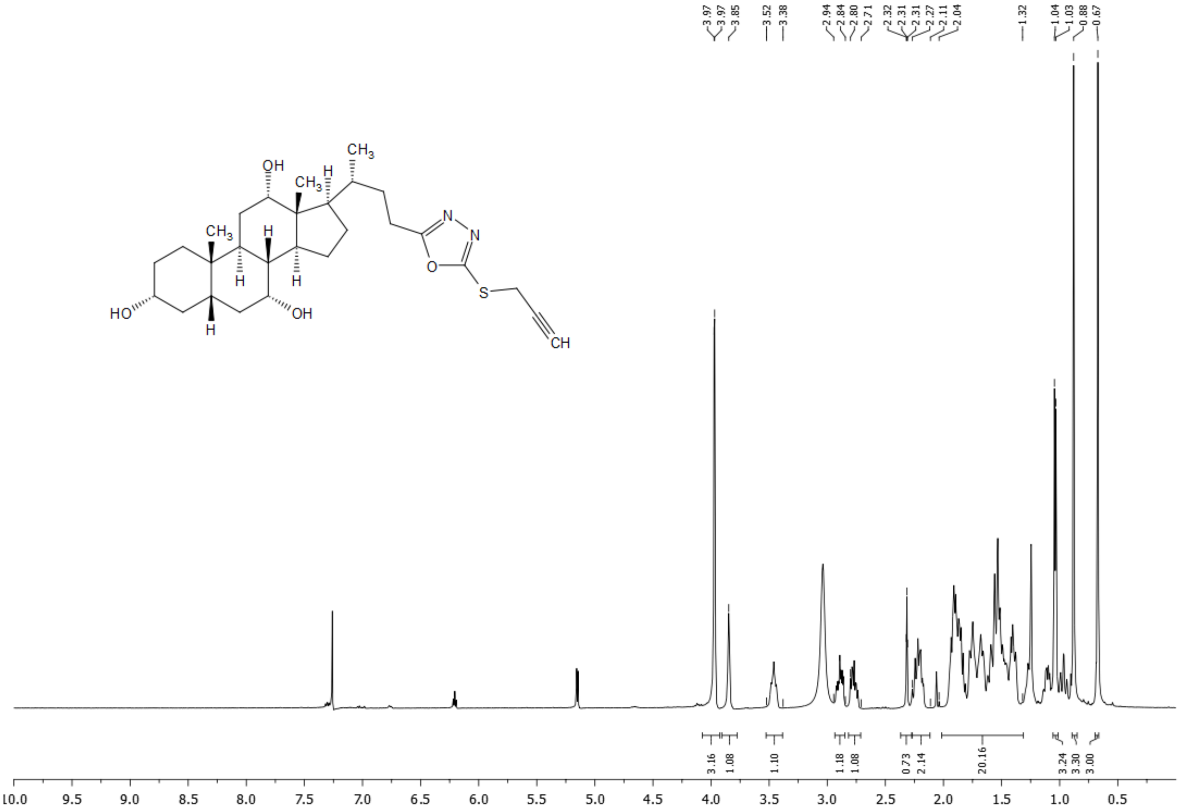

3.97
3.97
3.85
3.52
3.38
2.94
2.84
2.80
2.71
2.32
2.31
2.31
2.27
2.11
2.04
1.32
1.04
1.03
0.88
0.67
3.16
1.08
1.10
1.18
1.08
0.73
2.14
20.16
3.24
3.30
3.00

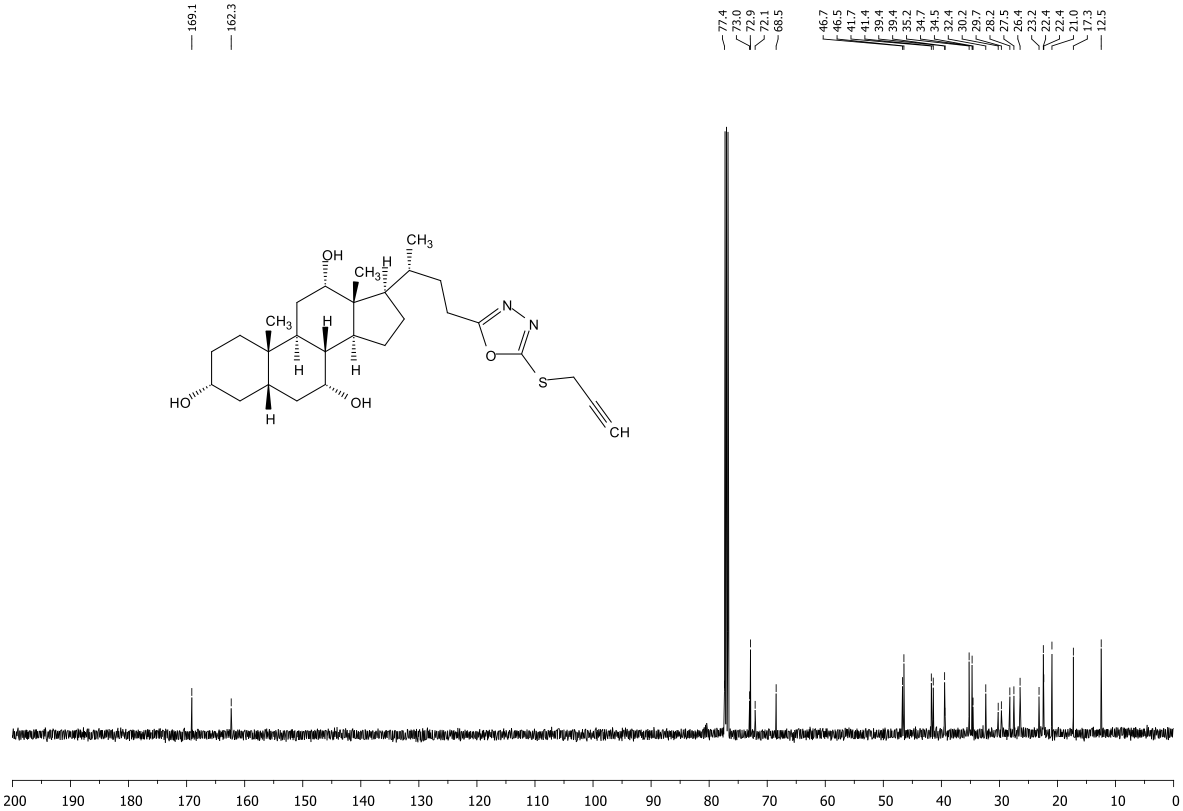

169.1
162.3
77.4
73.0
72.9
72.1
68.5
46.7
46.5
41.7
41.4
39.4
39.4
35.2
34.7
34.5
32.4
30.2
29.7
28.2
27.5
26.4
23.2
22.4
22.4
21.0
17.3
12.5

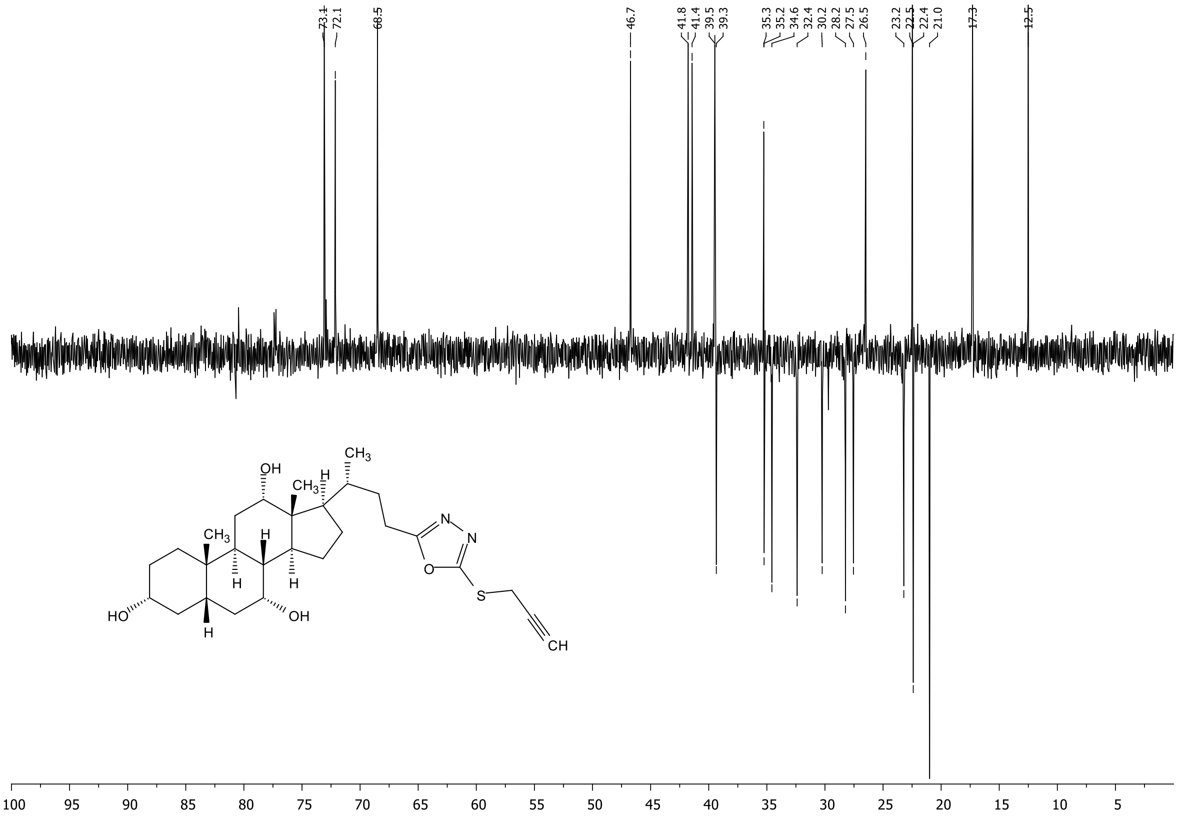

73.1
72.1
68.5
46.7
41.8
41.4
39.5
39.3
35.3
35.2
34.6
32.4
30.2
28.2
27.5
26.5
23.2
22.5
22.4
21.0
17.3
12.5
100 95 90 85 80 75 70 65 60 55 50 45 40 35 30 25 20 15 10 5

Compound 4a

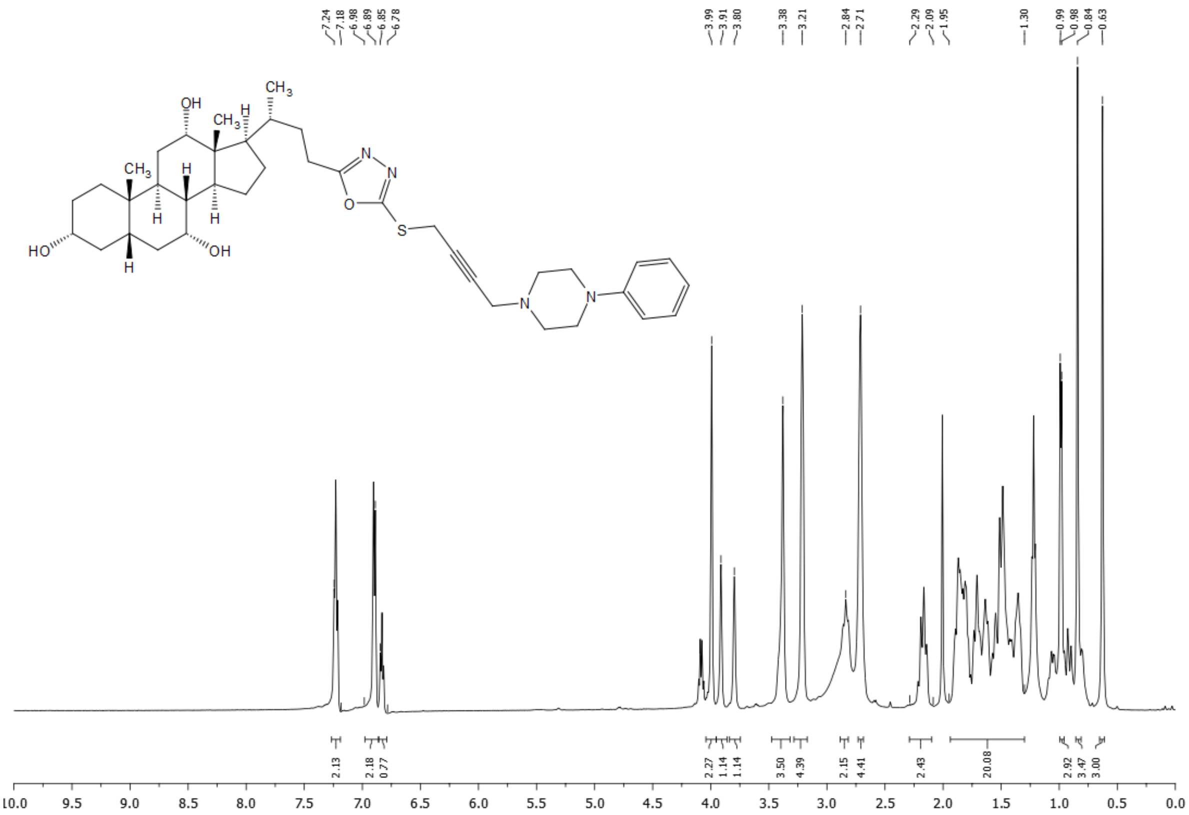
7.24
7.18
6.98
6.89
6.85
6.78
3.99
3.91
3.80
3.38
3.21
2.84
2.71
2.29
2.09
1.95
1.30
0.99
0.98
0.84
0.63
OH
CH3
H
CH3
CH3
H
H
H
HO
H
OH
N
N
O
S
N
N
2.13
2.18
0.77
2.27
1.14
1.14
3.50
4.39
2.15
4.41
2.43
20.08
2.92
3.47
3.00
10.0
9.5
9.0
8.5
8.0
7.5
7.0
6.5
6.0
5.5
5.0
4.5
4.0
3.5
3.0
2.5
2.0
1.5
1.0
0.5
0.0

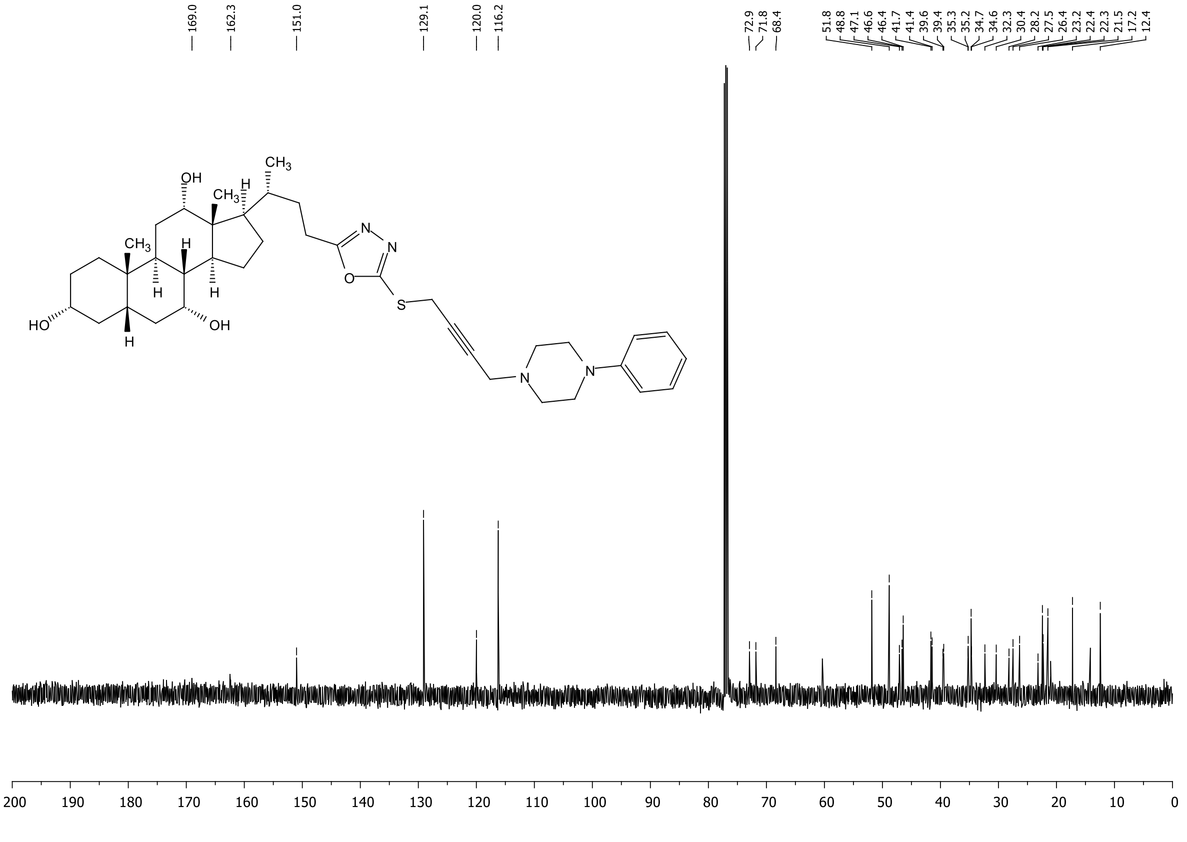
169.0
162.3
151.0
129.1
120.0
116.2
72.9
71.8
68.4
51.8
48.8
47.1
46.6
46.4
41.7
41.4
39.6
39.4
35.3
35.2
34.7
34.6
32.3
30.4
28.2
27.5
26.4
23.2
22.4
22.3
21.5
17.2
12.4
OH
CH3
H
CH3
CH3
H
H
H
HO
H
OH
N
N
O
S
N
N
200
190
180
170
160
150
140
130
120
110
100
90
80
70
60
50
40
30
20
10
0

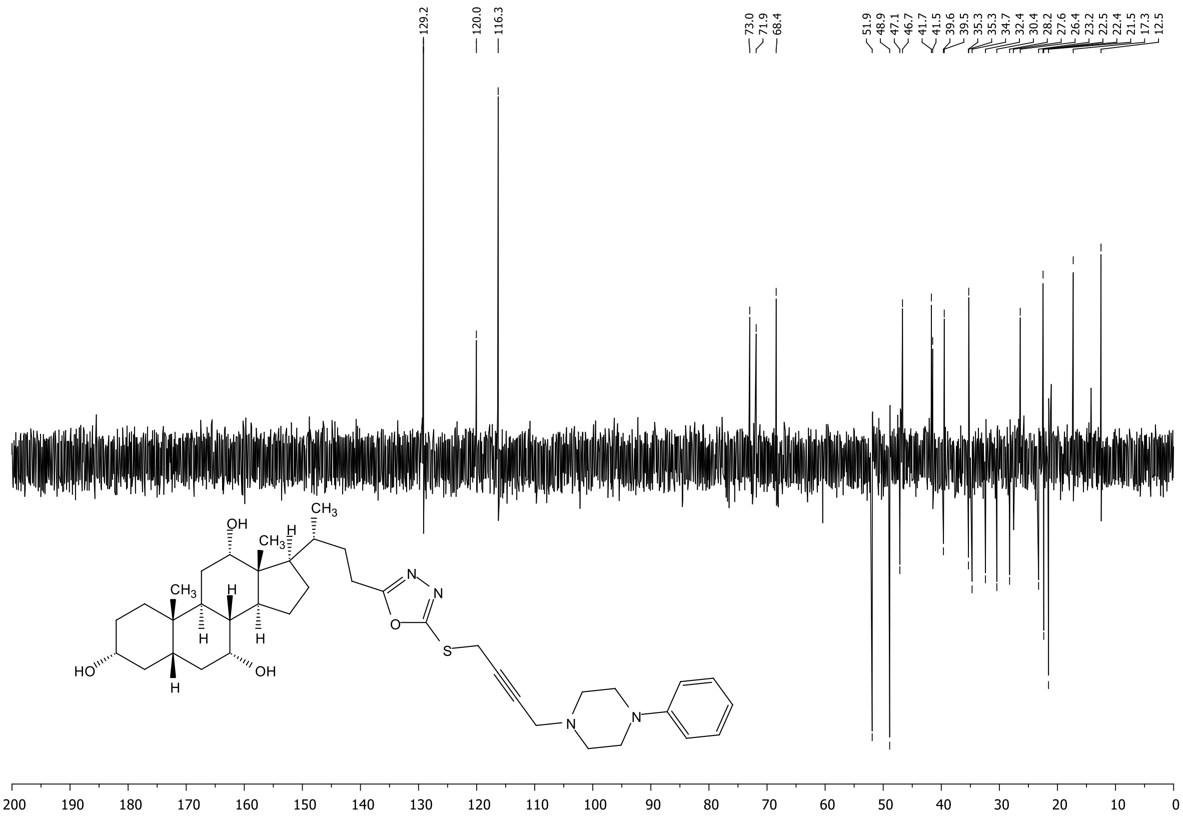

129.2
120.0
116.3
73.0
71.9
68.4
51.9
48.9
47.1
46.7
41.7
41.5
39.6
39.5
35.3
35.3
34.7
32.4
30.4
28.2
27.6
26.4
23.2
22.5
22.4
21.5
17.3
12.5
200 190 180 170 160 150 140 130 120 110 100 90 80 70 60 50 40 30 20 10 0

Compound 4b

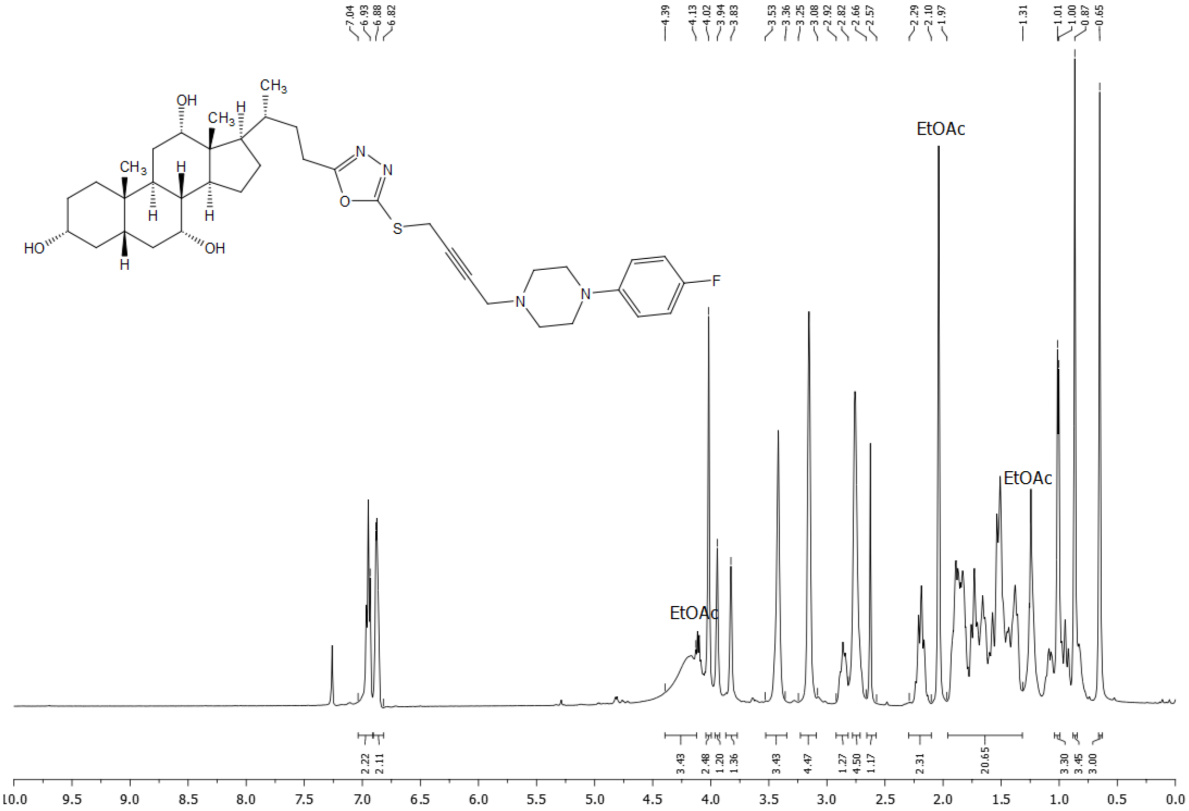

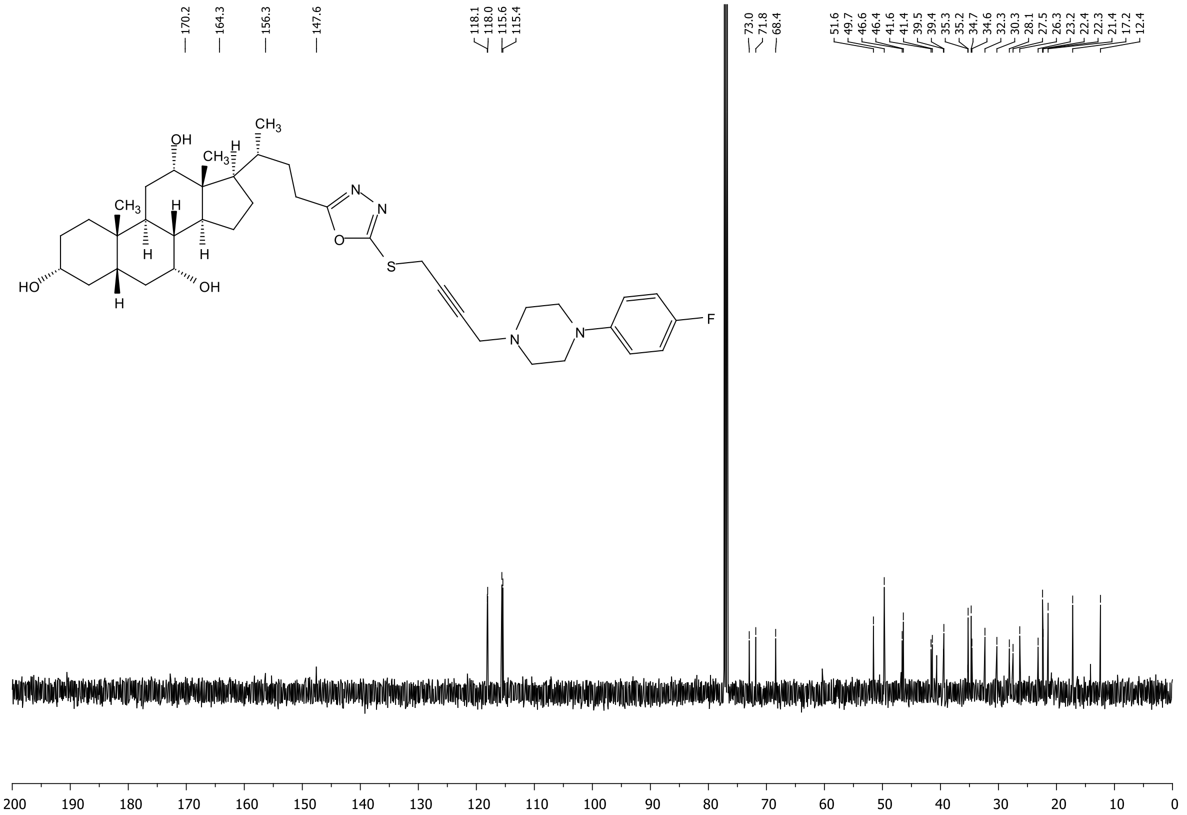

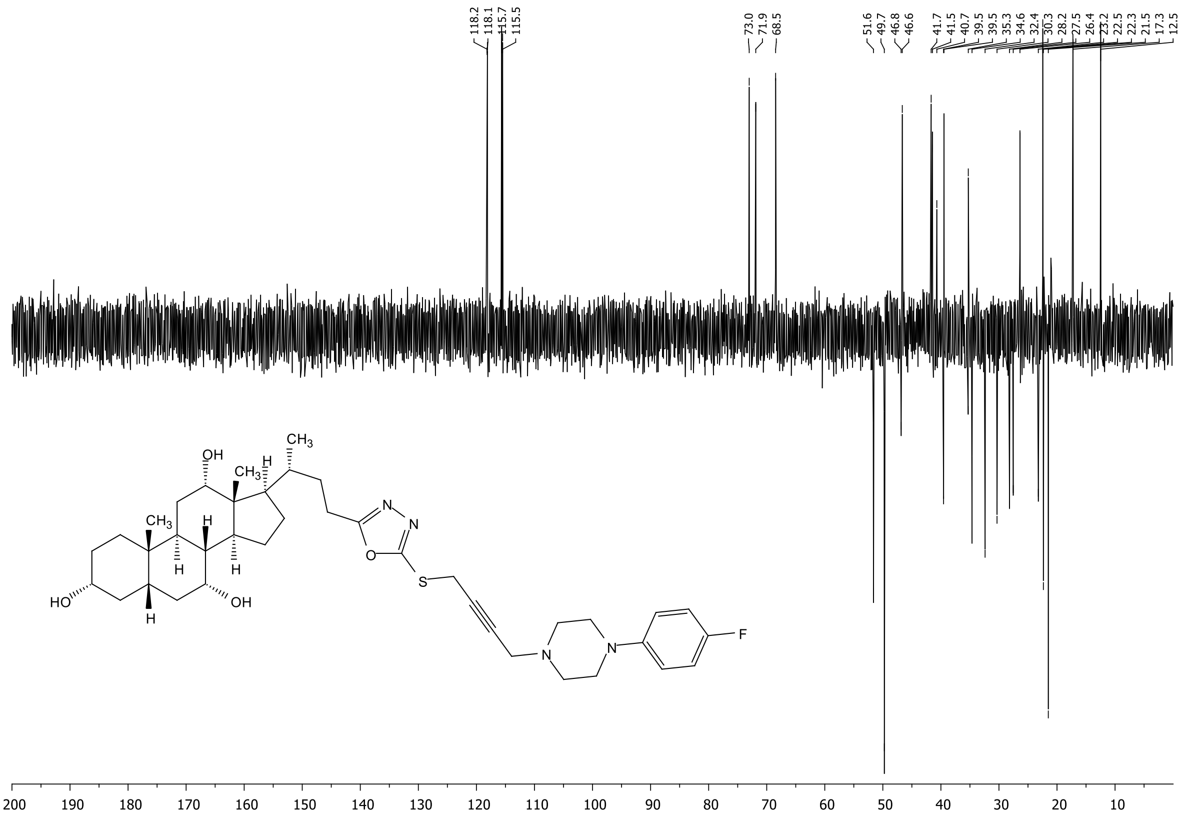

118.2
118.1
115.7
115.5
73.0
71.9
68.5
51.6
49.7
46.8
46.6
41.7
41.5
40.7
39.5
39.5
35.3
34.6
32.4
30.3
28.2
27.5
26.4
23.2
22.5
22.3
21.5
17.3
12.5
OH
CH3
H
CH3
CH3
H
H
HO
H
OH
H
N
N
O
S
N
N
F
200
190
180
170
160
150
140
130
120
110
100
90
80
70
60
50
40
30
20
10

Compound 4c

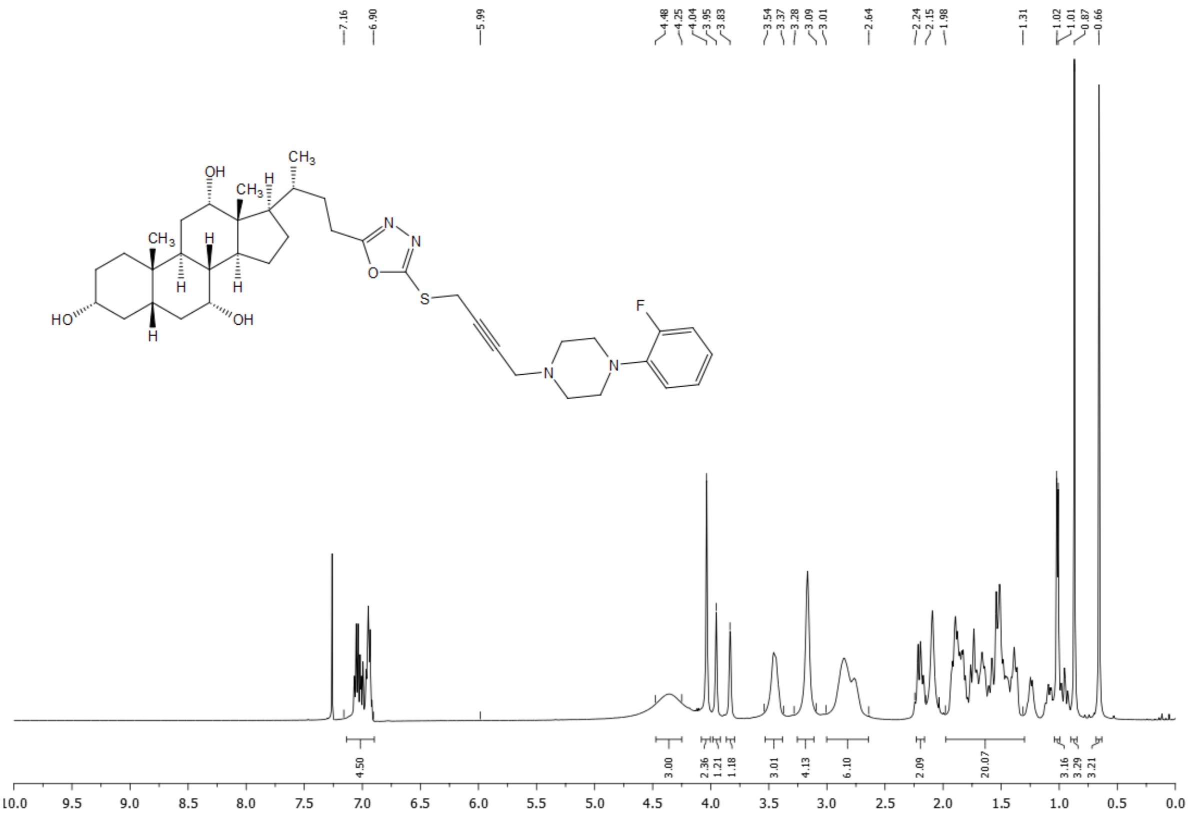
7.16
6.90
5.99
4.48
4.25
4.04
3.95
3.83
3.54
3.37
3.28
3.09
3.01
2.64
2.24
2.15
1.98
1.31
1.02
1.01
0.87
0.66
4.50
3.00
2.36
1.21
1.18
3.01
4.13
6.10
2.09
20.07
3.16
3.29
3.21
10.0 9.5 9.0 8.5 8.0 7.5 7.0 6.5 6.0 5.5 5.0 4.5 4.0 3.5 3.0 2.5 2.0 1.5 1.0 0.5 0.0

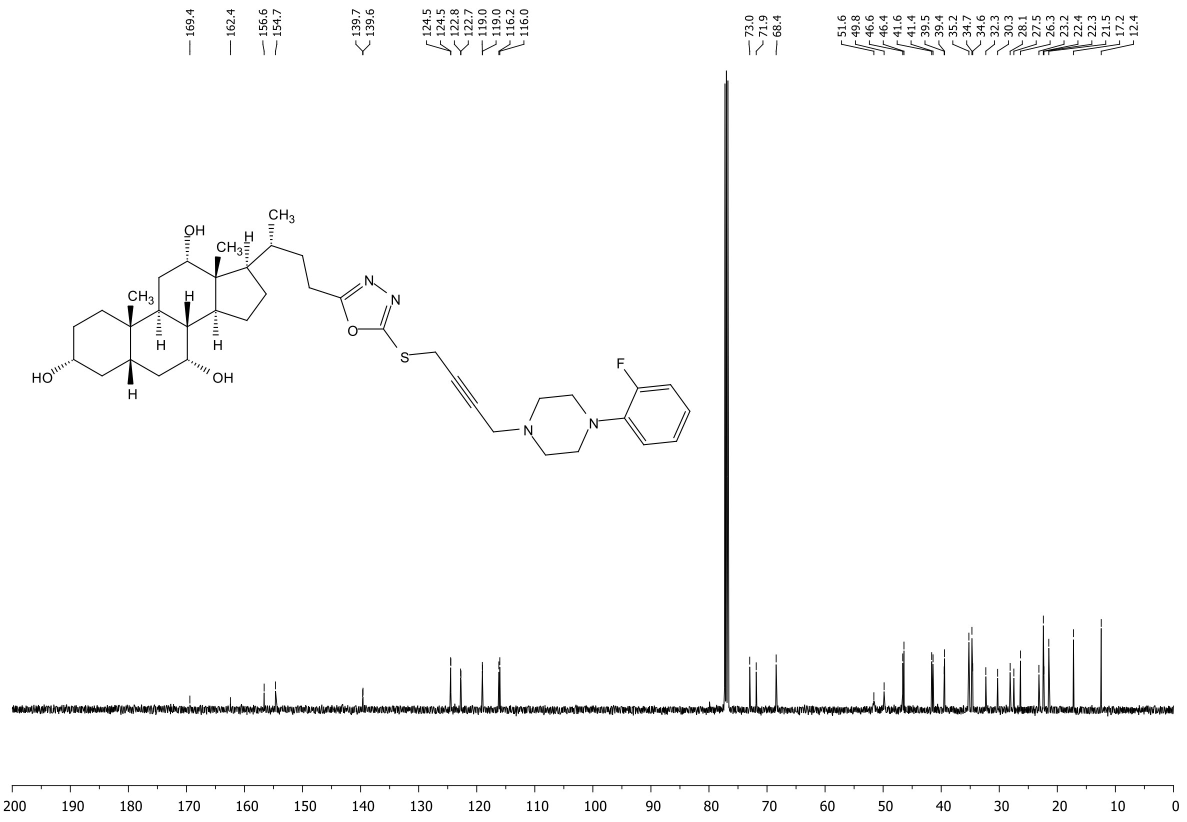
169.4
162.4
156.6
154.7
139.7
139.6
124.5
124.5
122.8
122.7
119.0
119.0
116.2
116.0
73.0
71.9
68.4
51.6
49.8
46.6
46.4
41.6
41.4
39.5
39.4
35.2
34.7
34.6
32.3
30.3
28.1
27.5
26.3
23.2
22.4
22.3
21.5
17.2
12.4
200 190 180 170 160 150 140 130 120 110 100 90 80 70 60 50 40 30 20 10 0

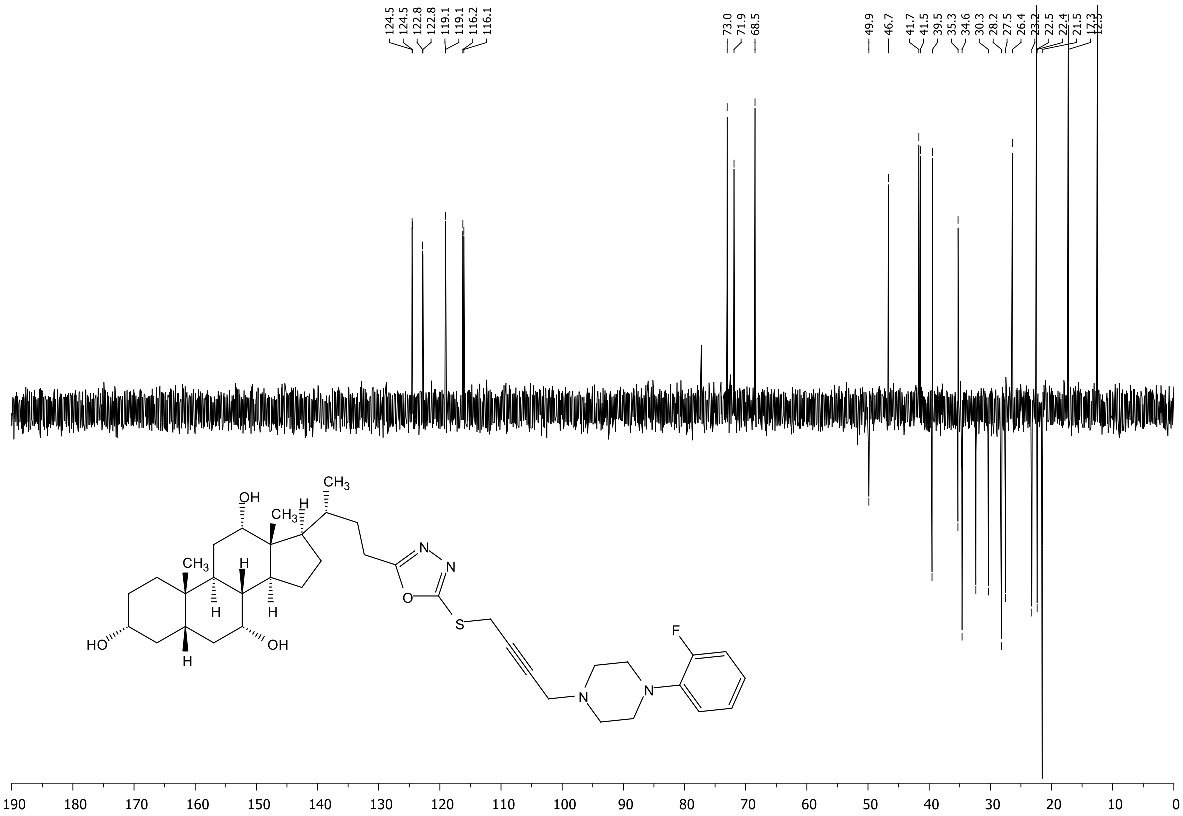
124.5
124.5
122.8
122.8
119.1
119.1
116.2
116.1
73.0
71.9
68.5
49.9
46.7
41.7
41.5
39.5
35.3
34.6
30.3
28.2
27.5
26.4
23.2
22.5
22.4
21.5
17.3
12.5
OH
CH3
H
N
N
O
S
F
N
N
HO
OH
190 180 170 160 150 140 130 120 110 100 90 80 70 60 50 40 30 20 10 0

Compound 4d

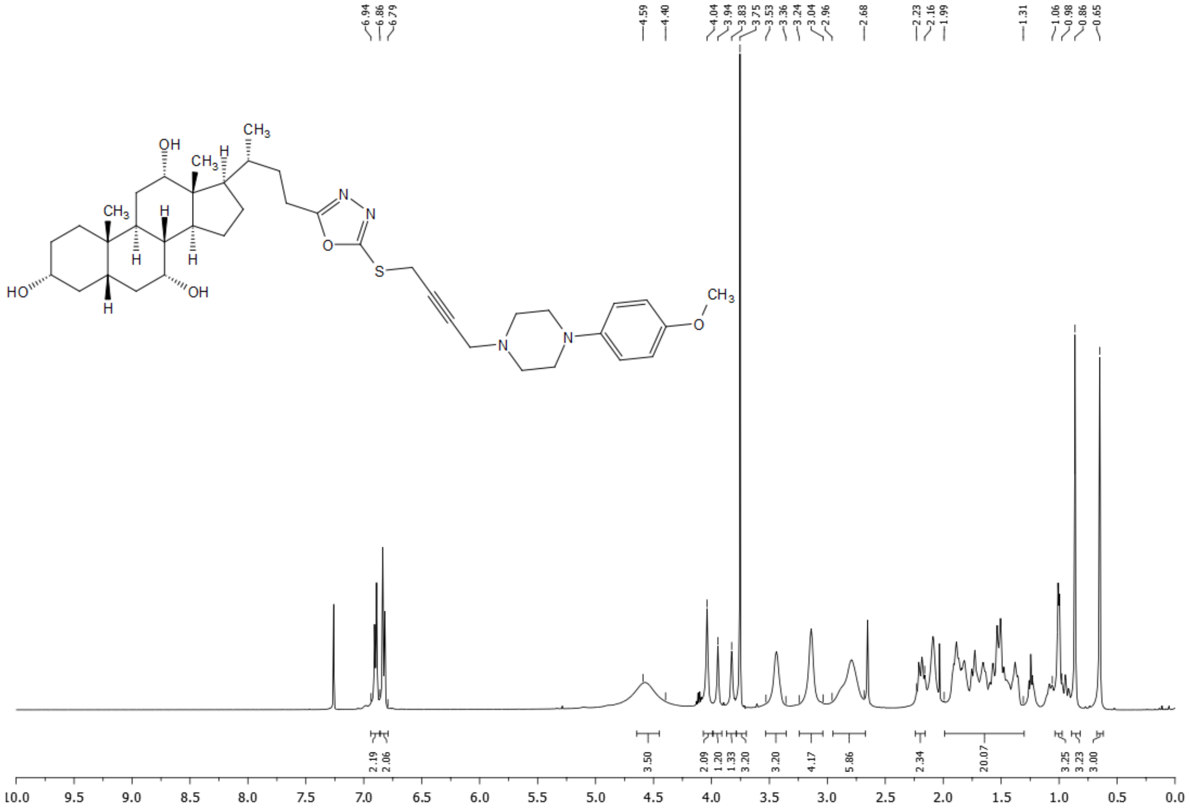

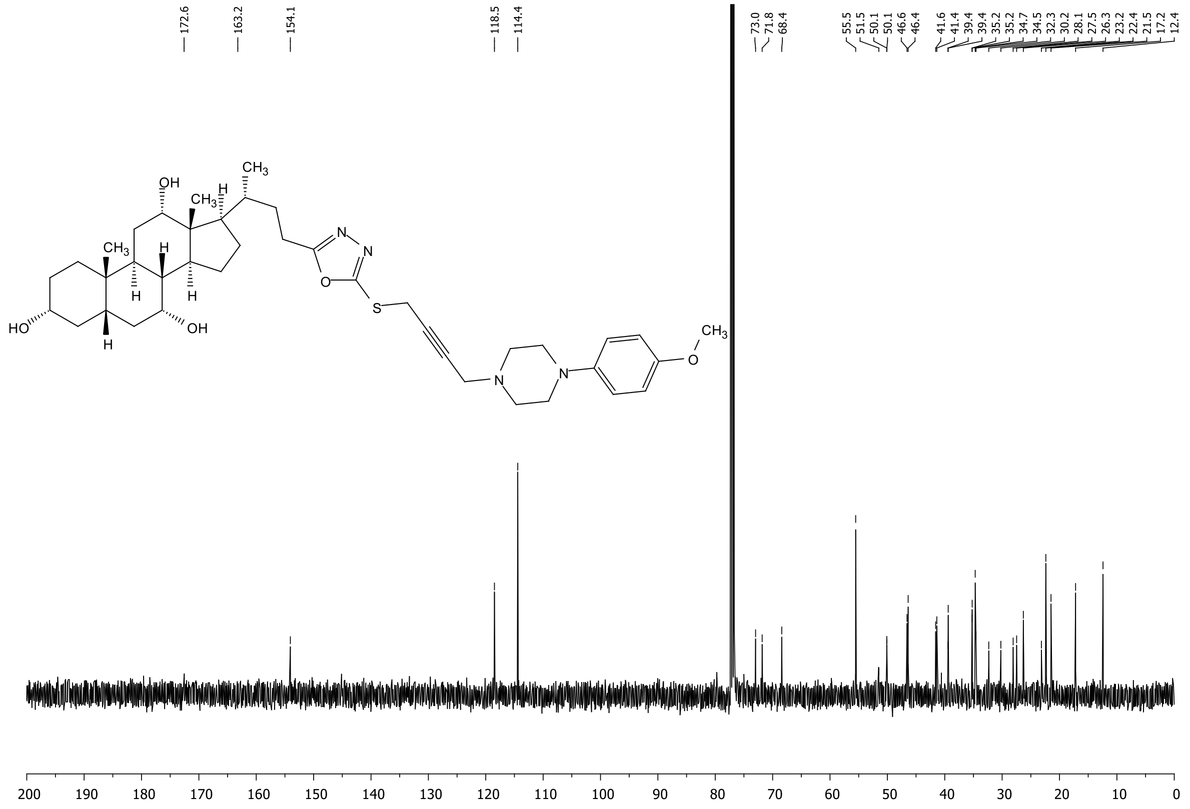

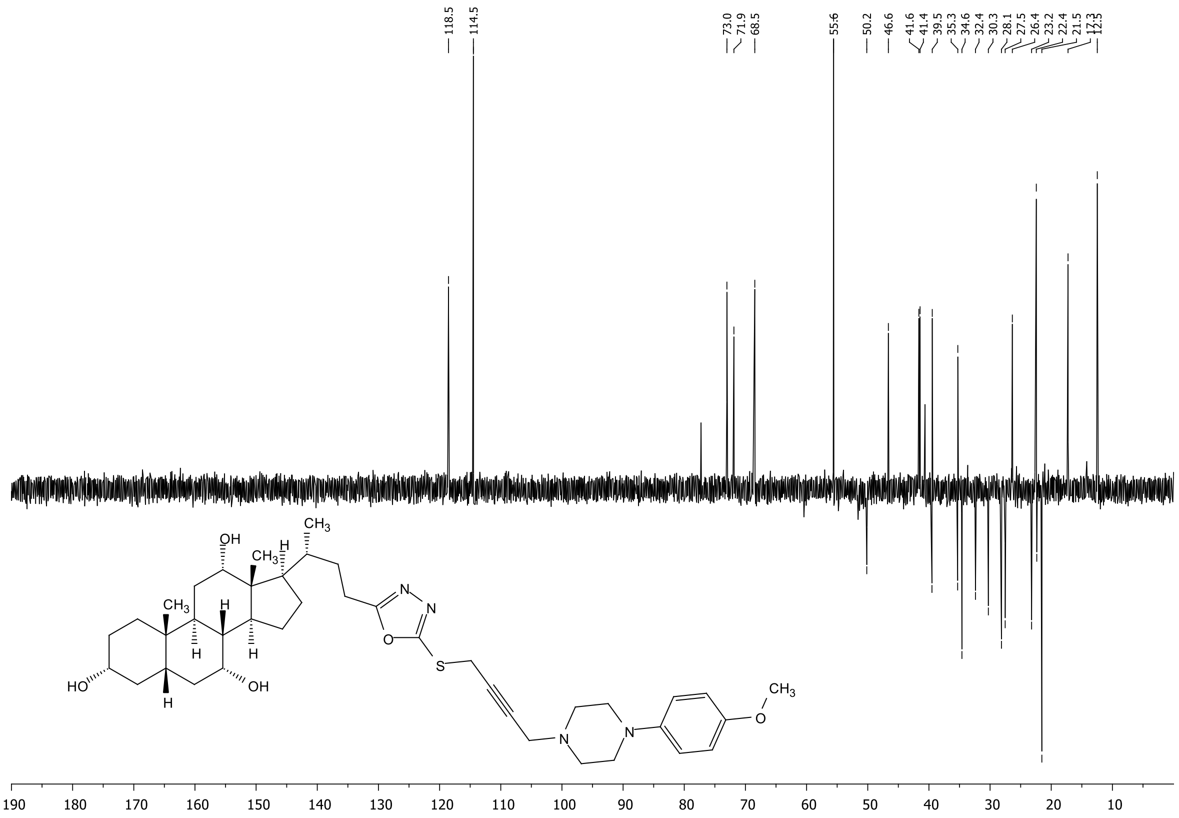

118.5
114.5
73.0
71.9
68.5
55.6
50.2
46.6
41.6
41.4
39.5
35.3
34.6
32.4
30.3
28.1
27.5
26.4
23.2
22.4
21.5
17.3
12.5
190 180 170 160 150 140 130 120 110 100 90 80 70 60 50 40 30 20 10

Compound 4e

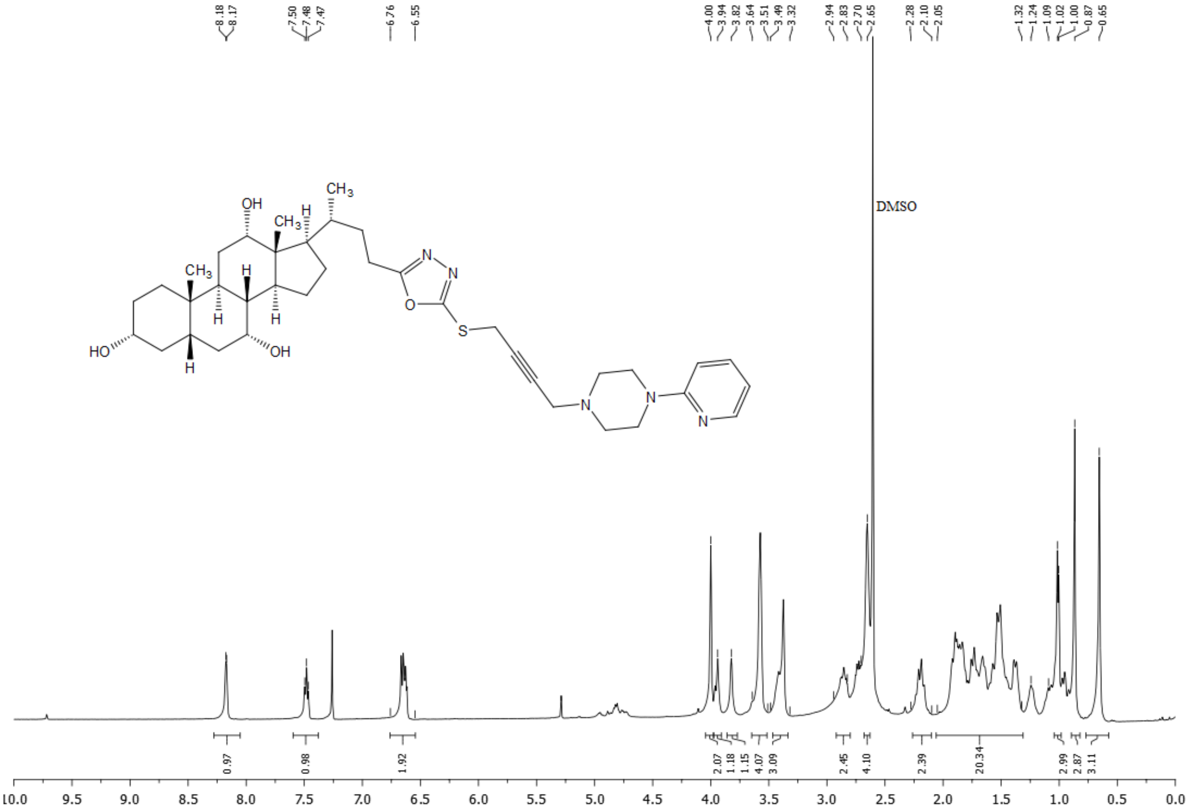
8.18
8.17
7.50
7.48
7.47
6.76
6.55
4.00
3.94
3.82
3.64
3.51
3.49
3.32
2.94
2.83
2.70
2.65
2.28
2.10
2.05
1.32
1.24
1.09
1.02
1.00
0.87
0.65
DMSO
0.97
0.98
1.92
2.07
1.18
1.15
4.07
3.09
2.45
4.10
2.39
20.34
2.99
2.87
3.11
10.0
9.5
9.0
8.5
8.0
7.5
7.0
6.5
6.0
5.5
5.0
4.5
4.0
3.5
3.0
2.5
2.0
1.5
1.0
0.5
0.0

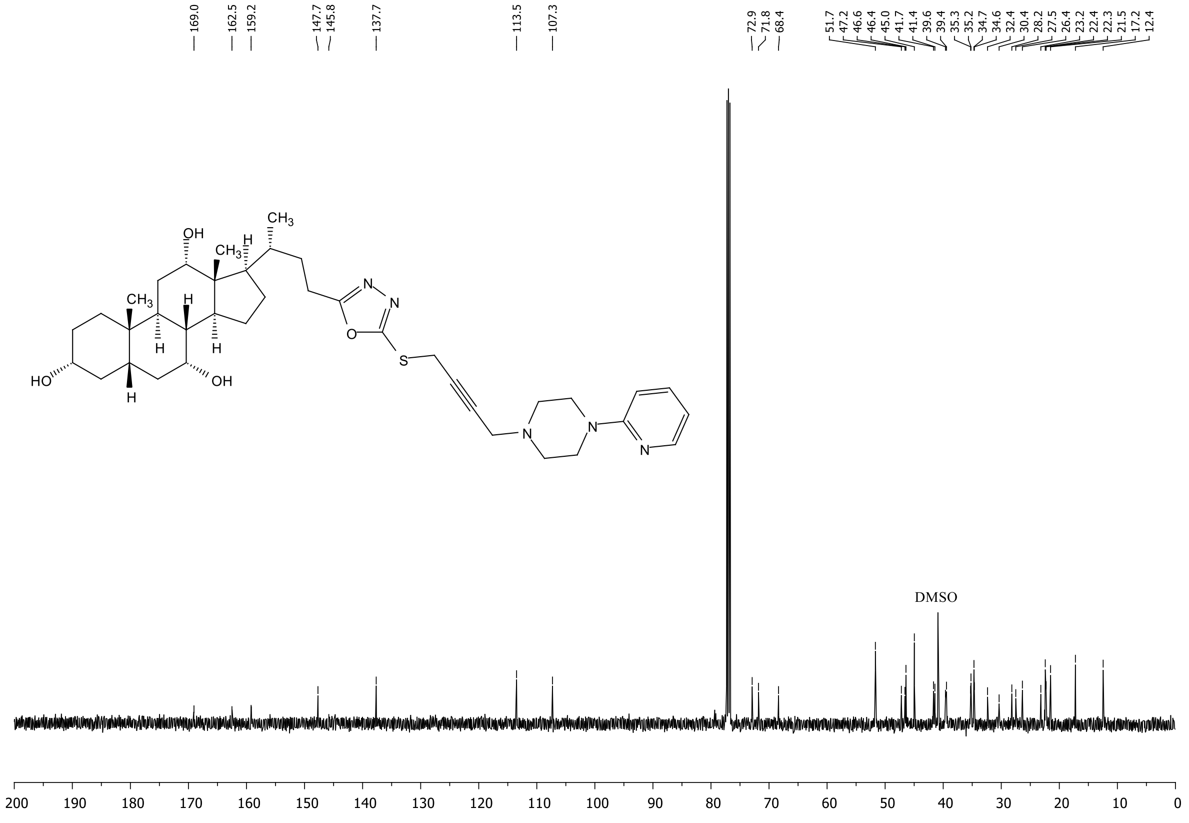
169.0
162.5
159.2
147.7
145.8
137.7
113.5
107.3
72.9
71.8
68.4
51.7
47.2
46.6
46.4
45.0
41.7
41.4
39.6
39.4
35.3
35.2
34.7
34.6
32.4
30.4
28.2
27.5
26.4
23.2
22.4
22.3
21.5
17.2
12.4
DMSO
200
190
180
170
160
150
140
130
120
110
100
90
80
70
60
50
40
30
20
10
0

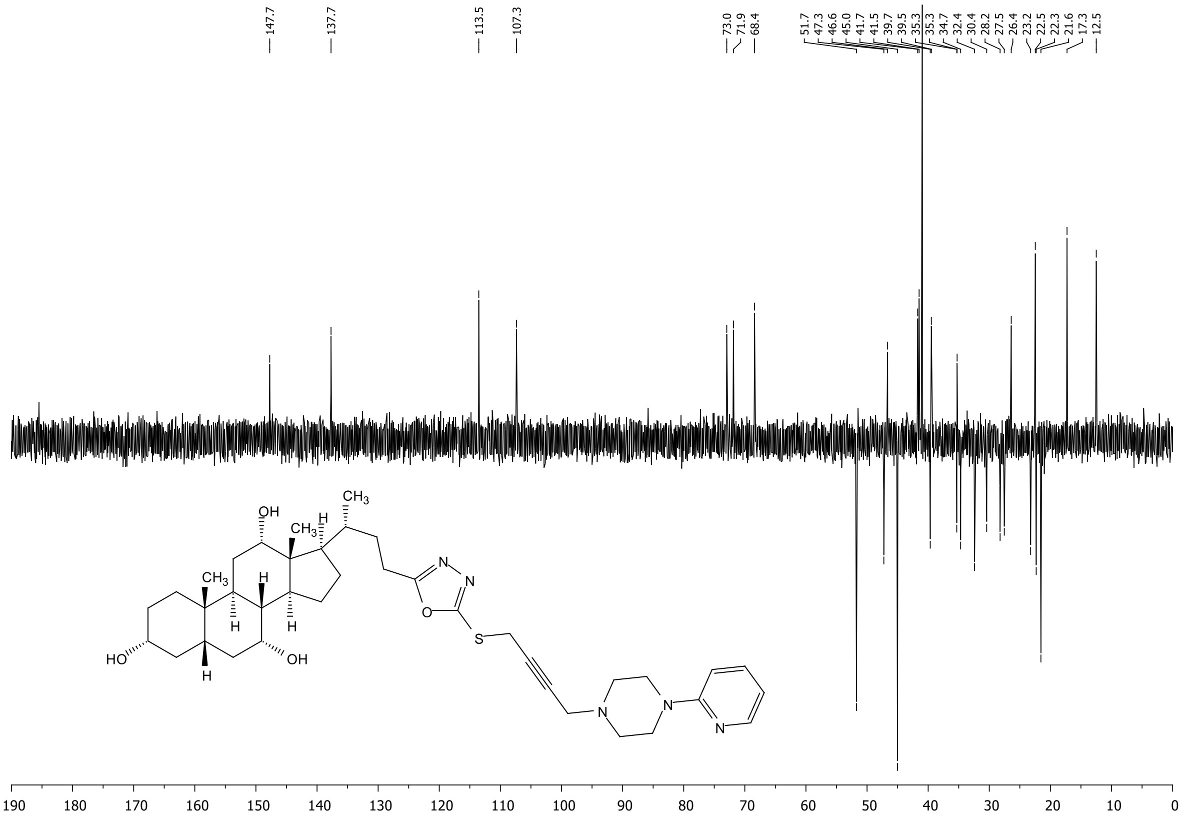
147.7
137.7
113.5
107.3
73.0
71.9
68.4
51.7
47.3
46.6
45.0
41.7
41.5
39.7
39.5
35.3
35.3
34.7
32.4
30.4
28.2
27.5
26.4
23.2
22.5
22.3
21.6
17.3
12.5
OH
CH3
H
CH3
H
H
H
HO
OH
H
N
N
O
S
N
N
N
190 180 170 160 150 140 130 120 110 100 90 80 70 60 50 40 30 20 10 0

Compound 4f

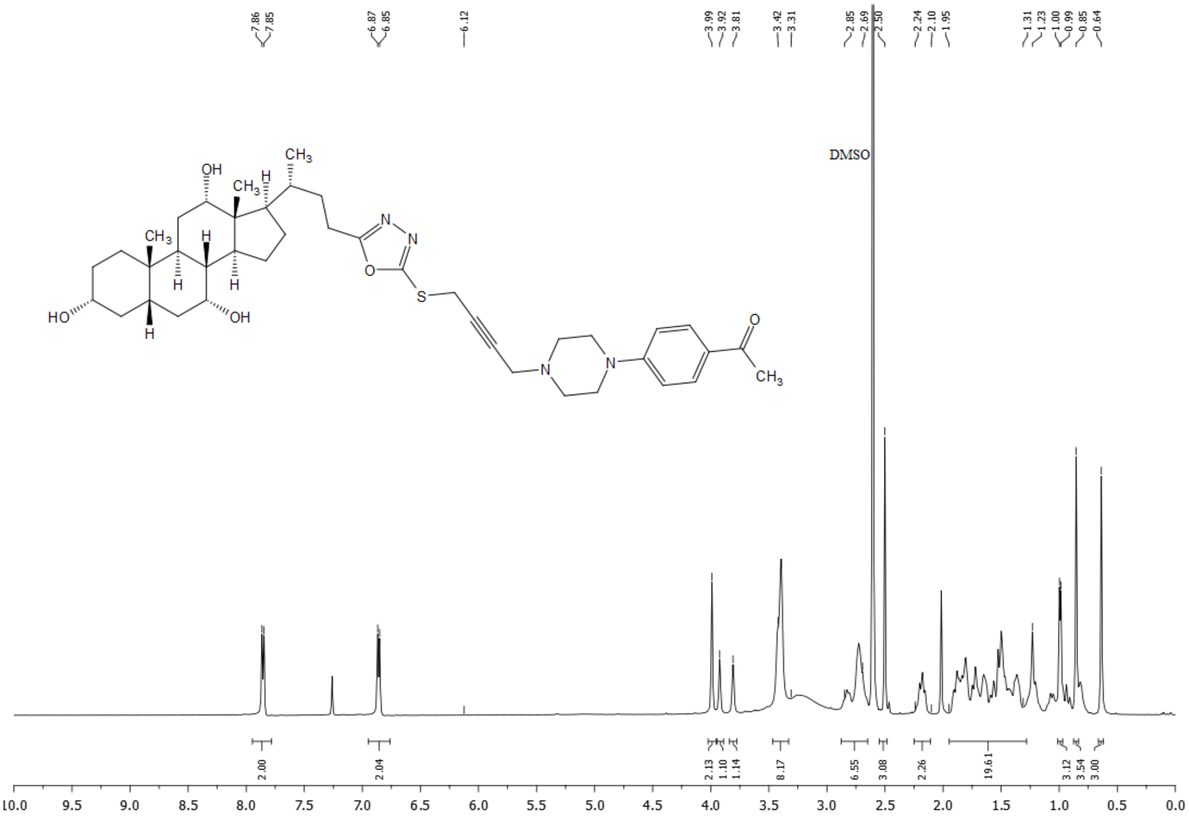
7.86
7.85
6.87
6.85
6.12
3.99
3.92
3.81
3.42
3.31
2.85
2.69
2.50
2.24
2.10
1.95
1.31
1.23
1.00
0.99
0.85
0.64
DMSO
OH
CH3
H
N
O
S
HO
CH3
2.00
2.04
2.13
1.10
1.14
8.17
6.55
3.08
2.26
19.61
3.12
3.54
3.00
10.0 9.5 9.0 8.5 8.0 7.5 7.0 6.5 6.0 5.5 5.0 4.5 4.0 3.5 3.0 2.5 2.0 1.5 1.0 0.5 0.0

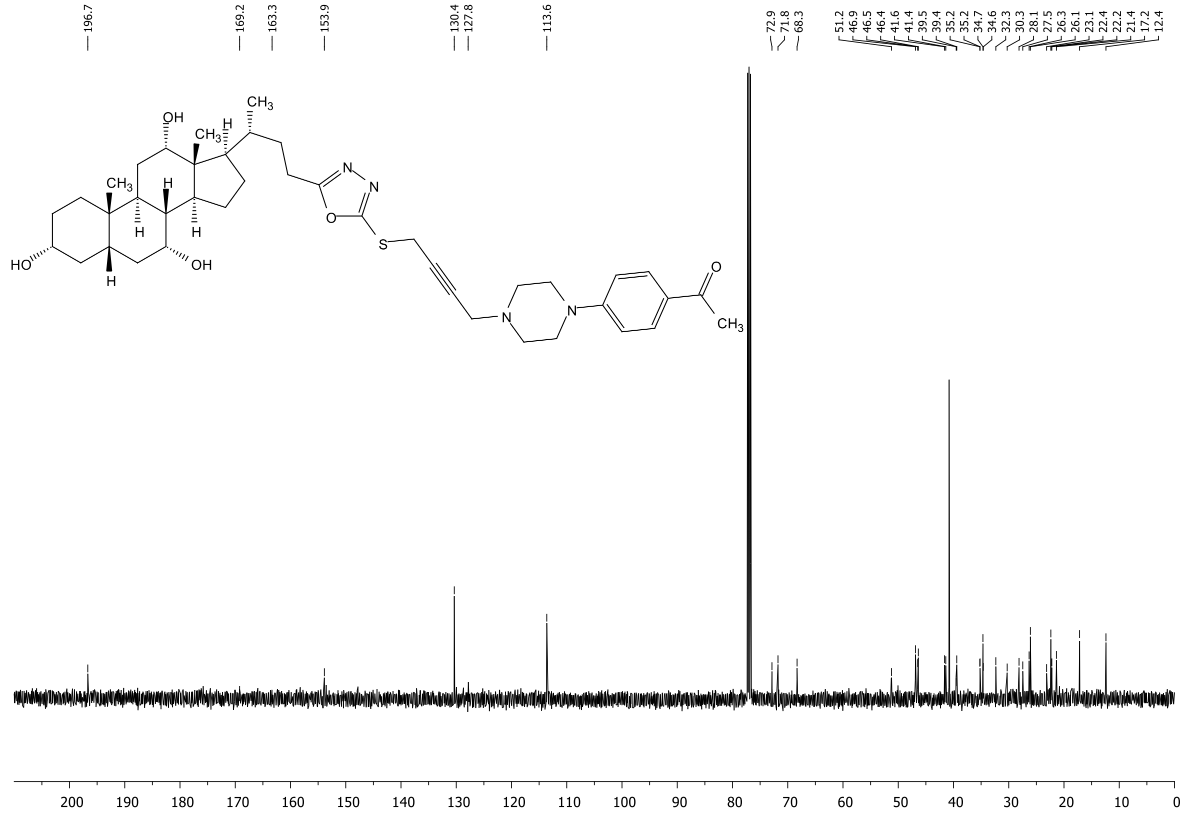
196.7
169.2
163.3
153.9
130.4
127.8
113.6
72.9
71.8
68.3
51.2
46.9
46.5
46.4
41.6
41.4
39.5
39.4
35.2
35.2
34.7
34.6
32.3
30.3
28.1
27.5
26.3
26.1
23.1
22.4
22.2
21.4
17.2
12.4
OH
CH3
H
N
O
S
HO
CH3
200 190 180 170 160 150 140 130 120 110 100 90 80 70 60 50 40 30 20 10 0

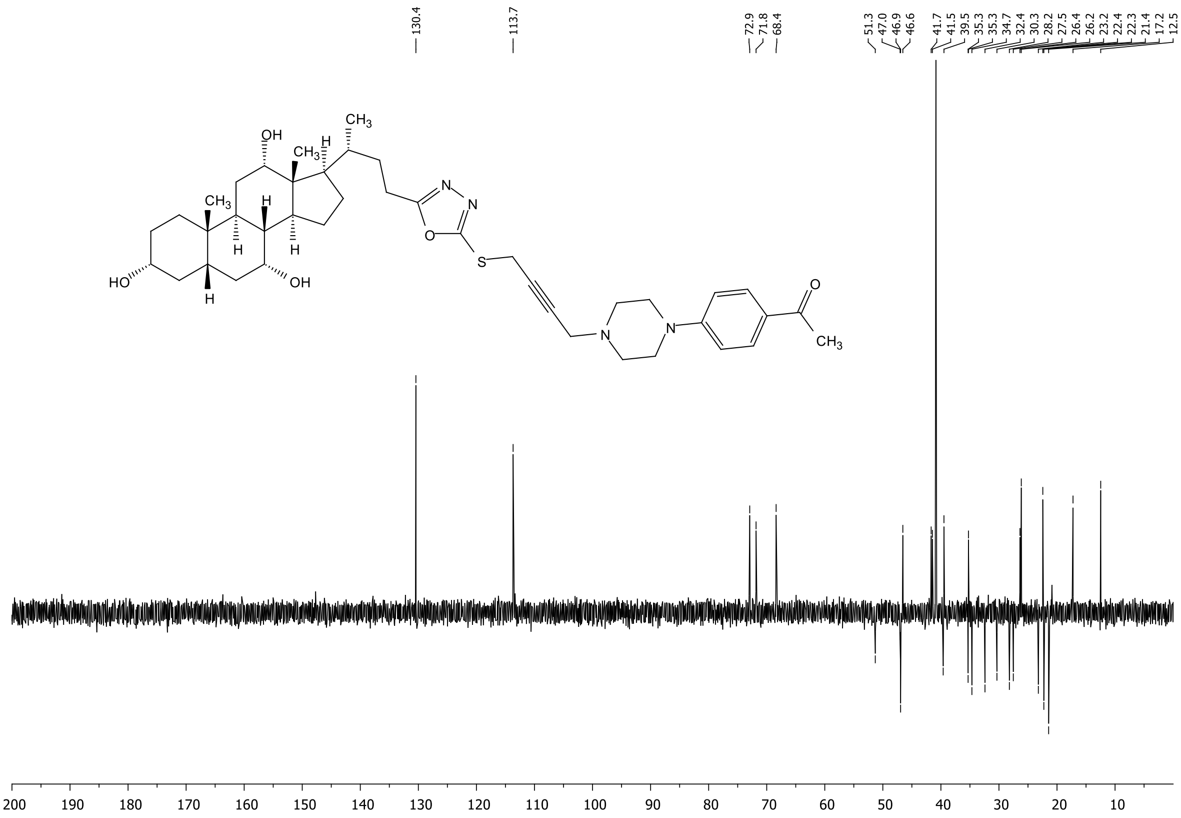
130.4
113.7
72.9
71.8
68.4
51.3
47.0
46.9
46.6
41.7
41.5
39.5
35.3
35.3
34.7
32.4
30.3
28.2
27.5
26.4
26.2
23.2
22.4
22.3
21.4
17.2
12.5
200 190 180 170 160 150 140 130 120 110 100 90 80 70 60 50 40 30 20 10

Compound 4g

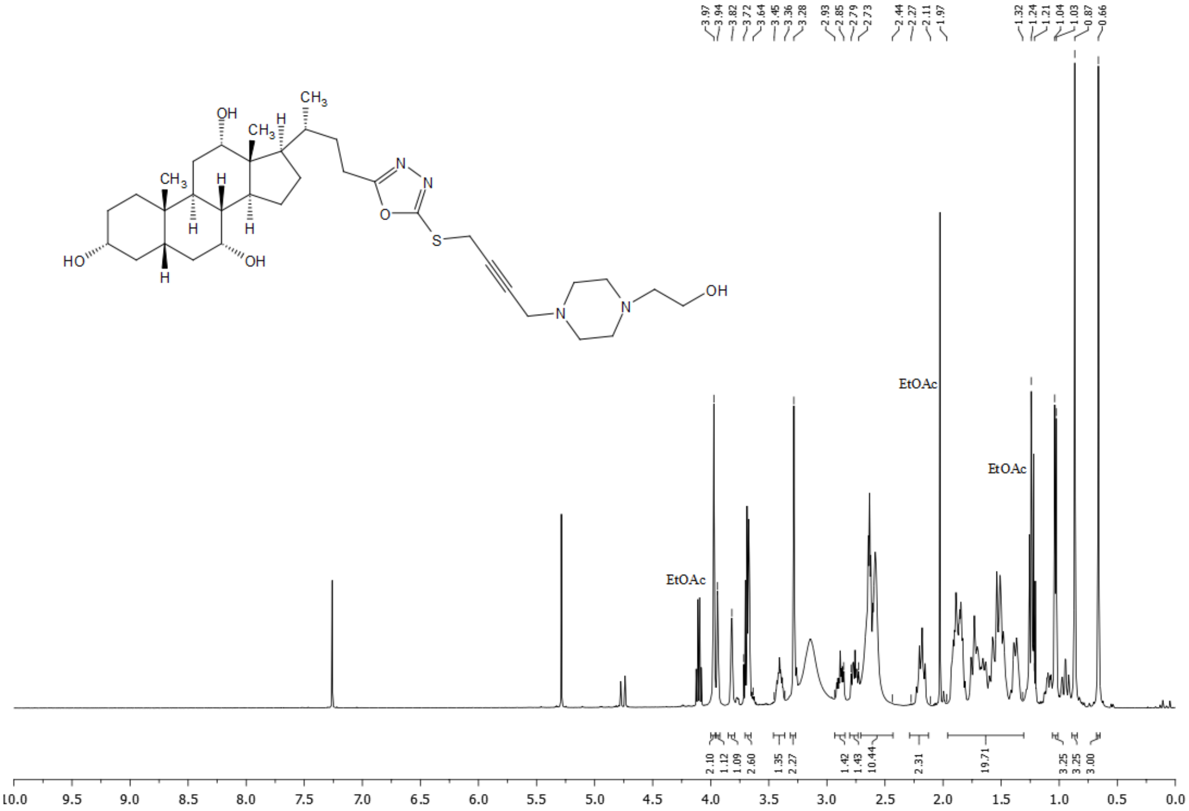
3.97 3.94 3.82 3.72 3.64 3.45 3.36 3.28 2.93 2.85 2.79 2.73 2.44 2.27 2.11 1.97 1.32 1.24 1.21 1.04 1.03 0.87 0.66
EtOAc
EtOAc
EtOAc
2.10 1.12 1.09 2.60 1.35 2.27 1.42 1.43 10.44 2.31 19.71 3.25 3.25 3.00
10.0 9.5 9.0 8.5 8.0 7.5 7.0 6.5 6.0 5.5 5.0 4.5 4.0 3.5 3.0 2.5 2.0 1.5 1.0 0.5 0.0
OH
CH3
CH3
H
H
H
H
H
HO
OH
N
N
O
S
N
N
OH

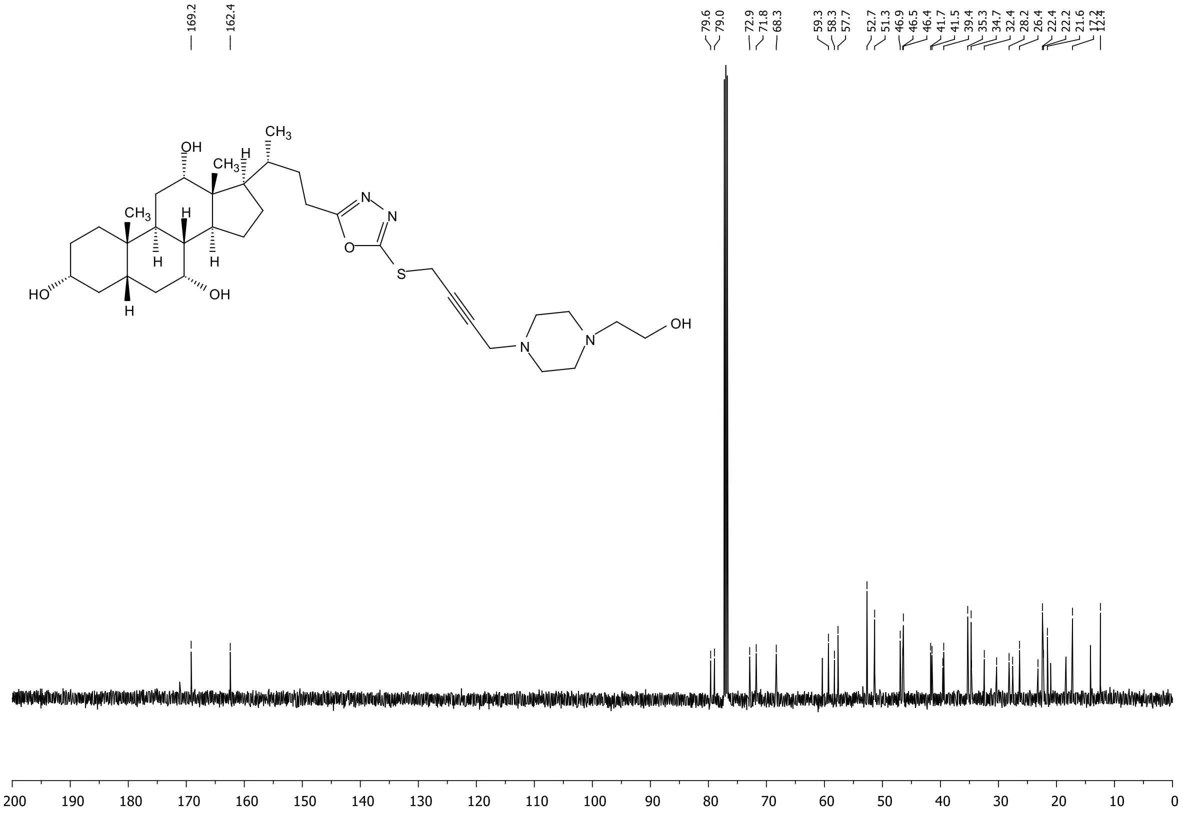
169.2 162.4 79.6 79.0 72.9 71.8 68.3 59.3 58.3 57.7 52.7 51.3 46.9 46.5 46.4 41.7 41.5 39.4 35.3 34.7 32.4 28.2 26.4 22.4 22.2 21.6 17.2 12.4
200 190 180 170 160 150 140 130 120 110 100 90 80 70 60 50 40 30 20 10 0
OH
CH3
CH3
H
H
H
H
H
HO
OH
N
N
O
S
N
N
OH

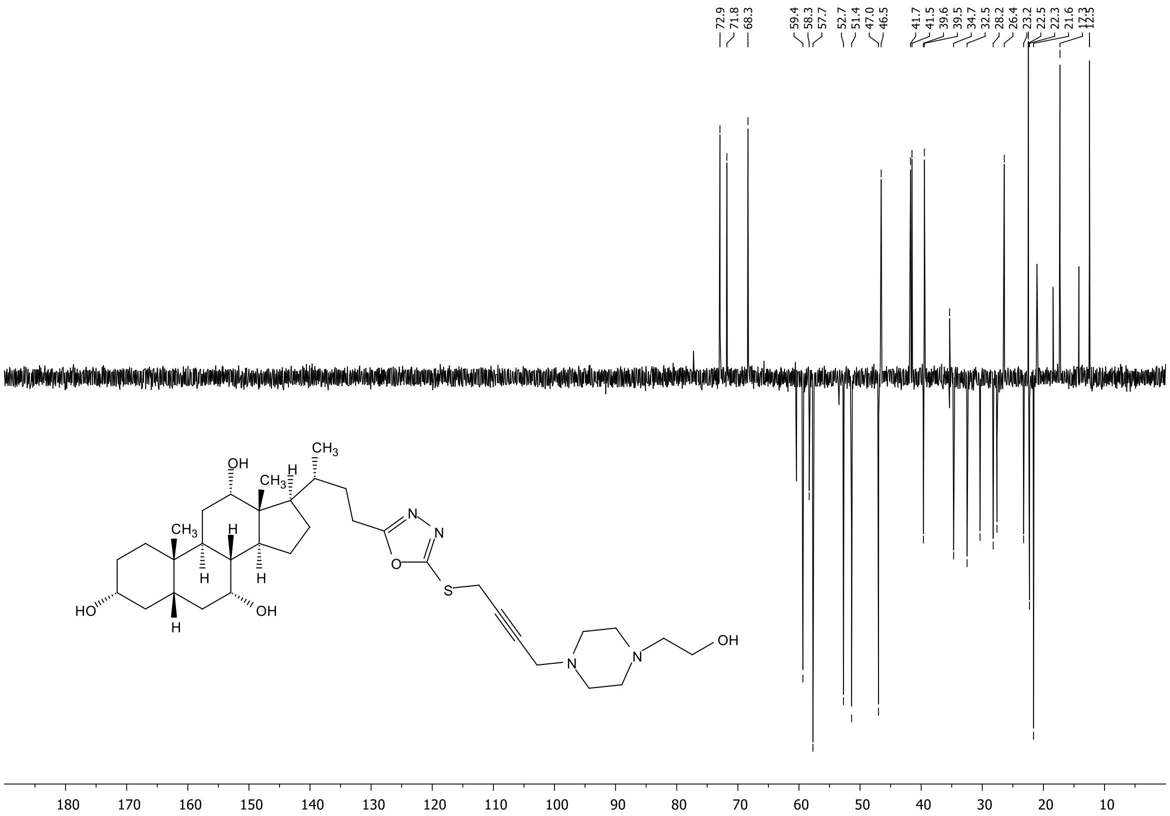
72.9
71.8
68.3
59.4
58.3
57.7
52.7
51.4
47.0
46.5
41.7
41.5
39.6
39.5
34.7
32.5
28.2
26.4
23.2
22.5
22.3
21.6
17.3
12.5
CH3
OH
CH3
H
CH3
H
N
N
O
S
HO
H
H
H
OH
N
N
OH
180
170
160
150
140
130
120
110
100
90
80
70
60
50
40
30
20
10

Compound 4h

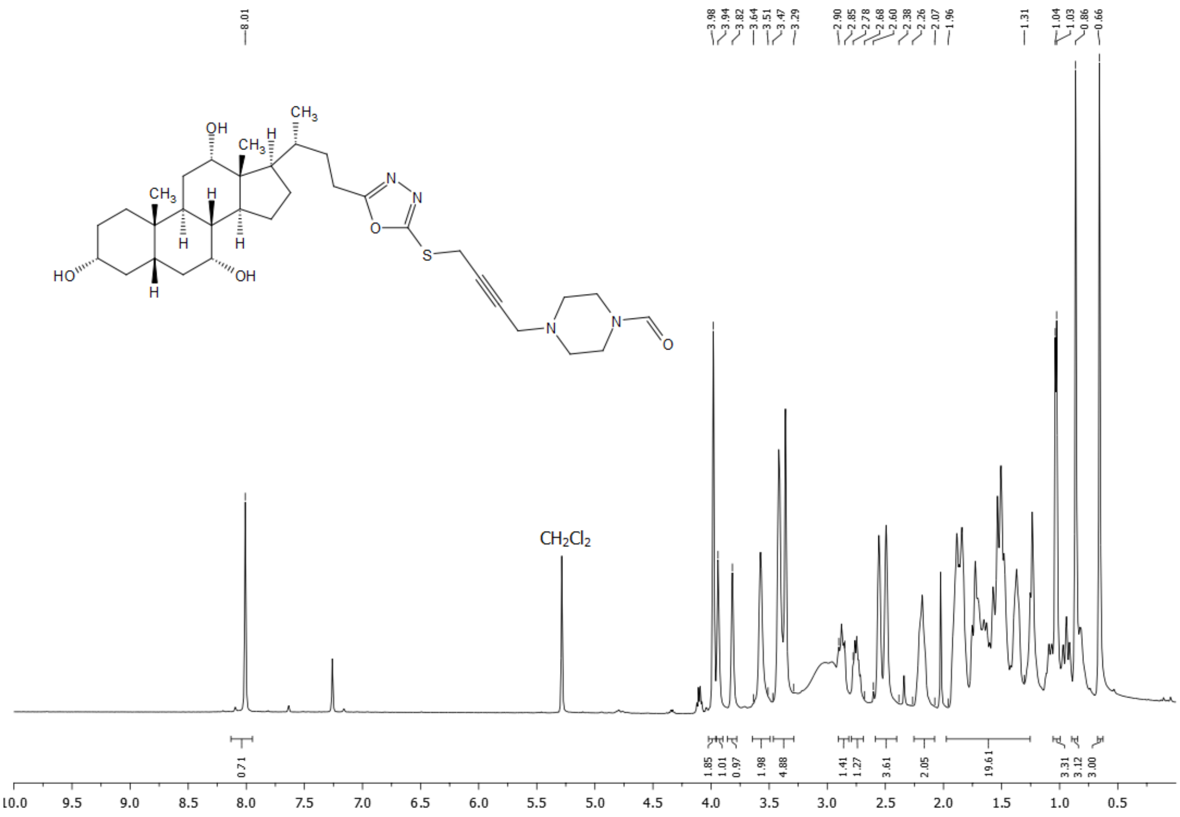

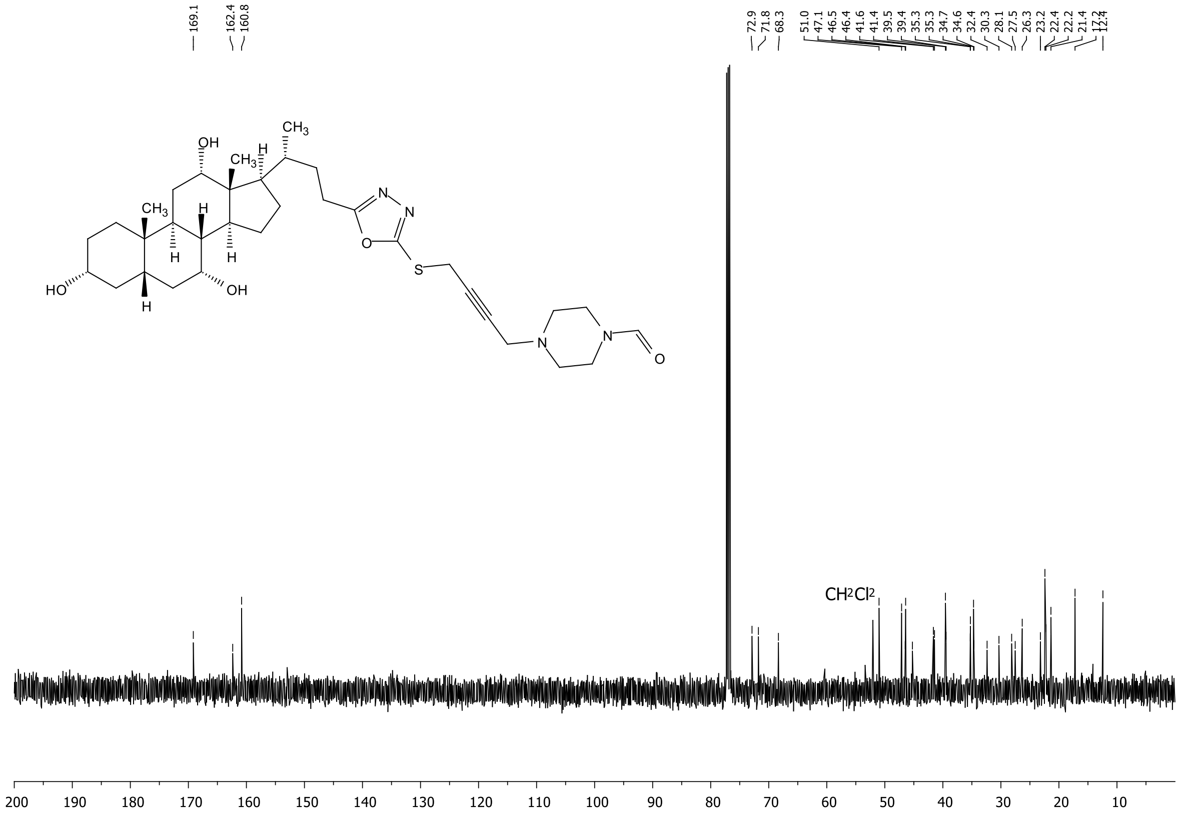

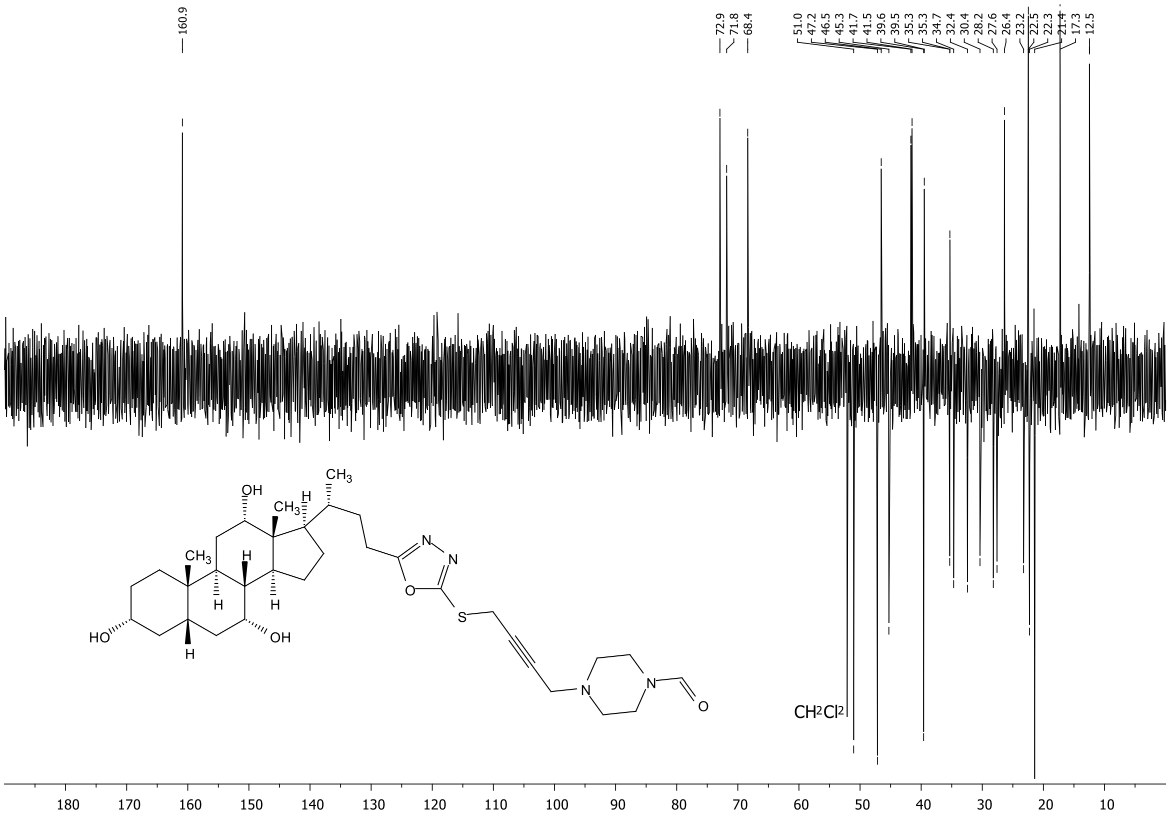
160.9
72.9
71.8
68.4
51.0
47.2
46.5
45.3
41.7
41.5
39.6
39.5
35.3
35.3
34.7
32.4
30.4
28.2
27.6
26.4
23.2
22.5
22.3
21.4
17.3
12.5
CH2Cl2
180 170 160 150 140 130 120 110 100 90 80 70 60 50 40 30 20 10

Compound 4i

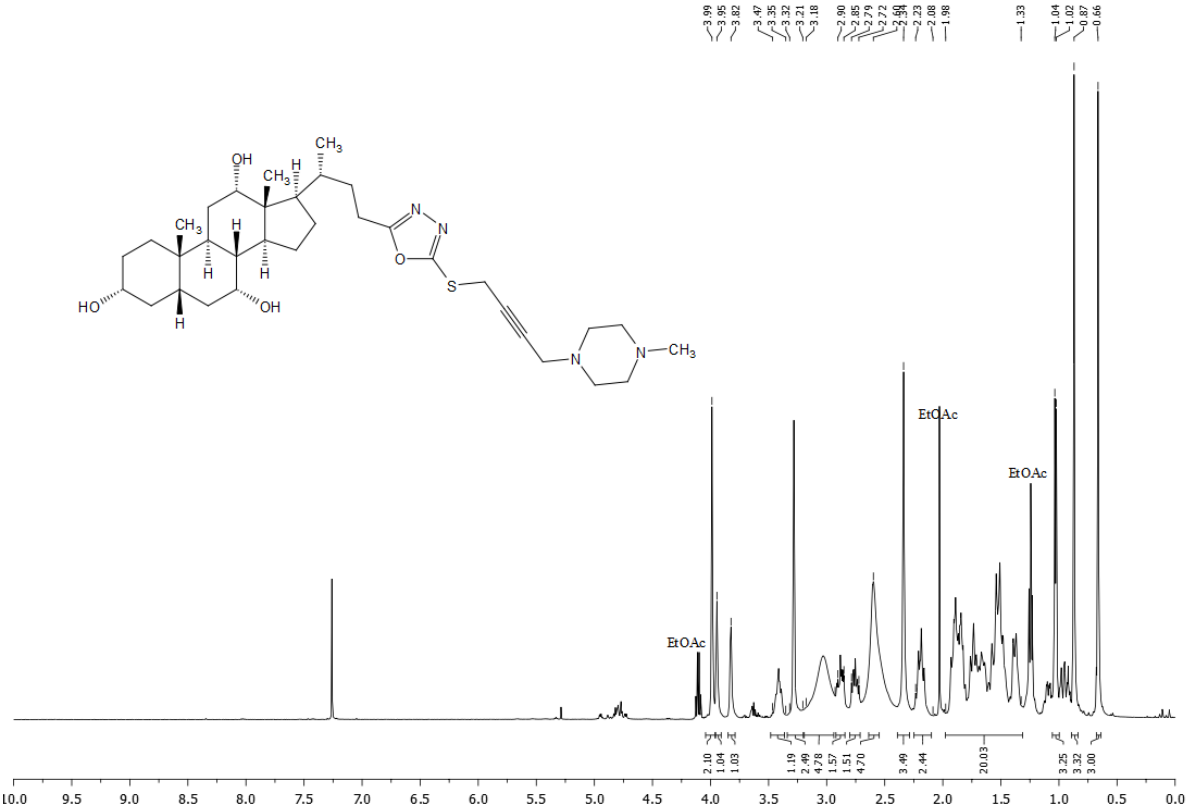
3.99
3.95
3.82
3.47
3.35
3.32
3.21
3.18
2.90
2.85
2.79
2.72
2.23
2.08
1.98
1.33
1.04
1.02
0.87
0.66
OH
CH3
H
CH3
H
H
H
H
HO
OH
N
N
O
S
N
N
CH3
EtOAc
EtOAc
EtOAc
2.10
1.04
1.03
1.19
2.49
4.78
1.57
1.51
4.70
3.49
2.44
20.03
3.25
3.32
3.00
10.0
9.5
9.0
8.5
8.0
7.5
7.0
6.5
6.0
5.5
5.0
4.5
4.0
3.5
3.0
2.5
2.0
1.5
1.0
0.5
0.0

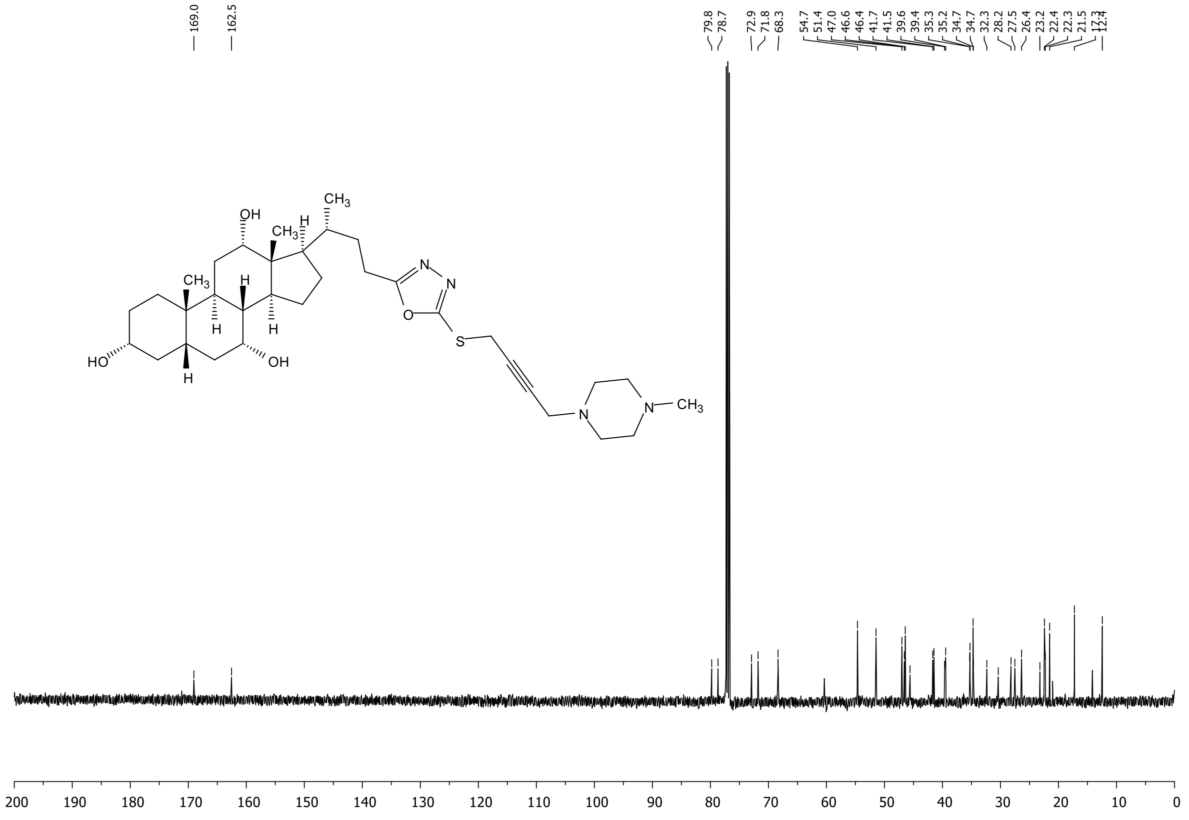
169.0
162.5
79.8
78.7
72.9
71.8
68.3
54.7
51.4
47.0
46.6
46.4
41.7
41.5
39.6
39.4
35.3
35.2
34.7
34.7
32.3
28.2
27.5
26.4
23.2
22.4
22.3
21.5
17.3
12.4
OH
CH3
H
CH3
H
H
H
H
HO
OH
N
N
O
S
N
N
CH3
200
190
180
170
160
150
140
130
120
110
100
90
80
70
60
50
40
30
20
10
0

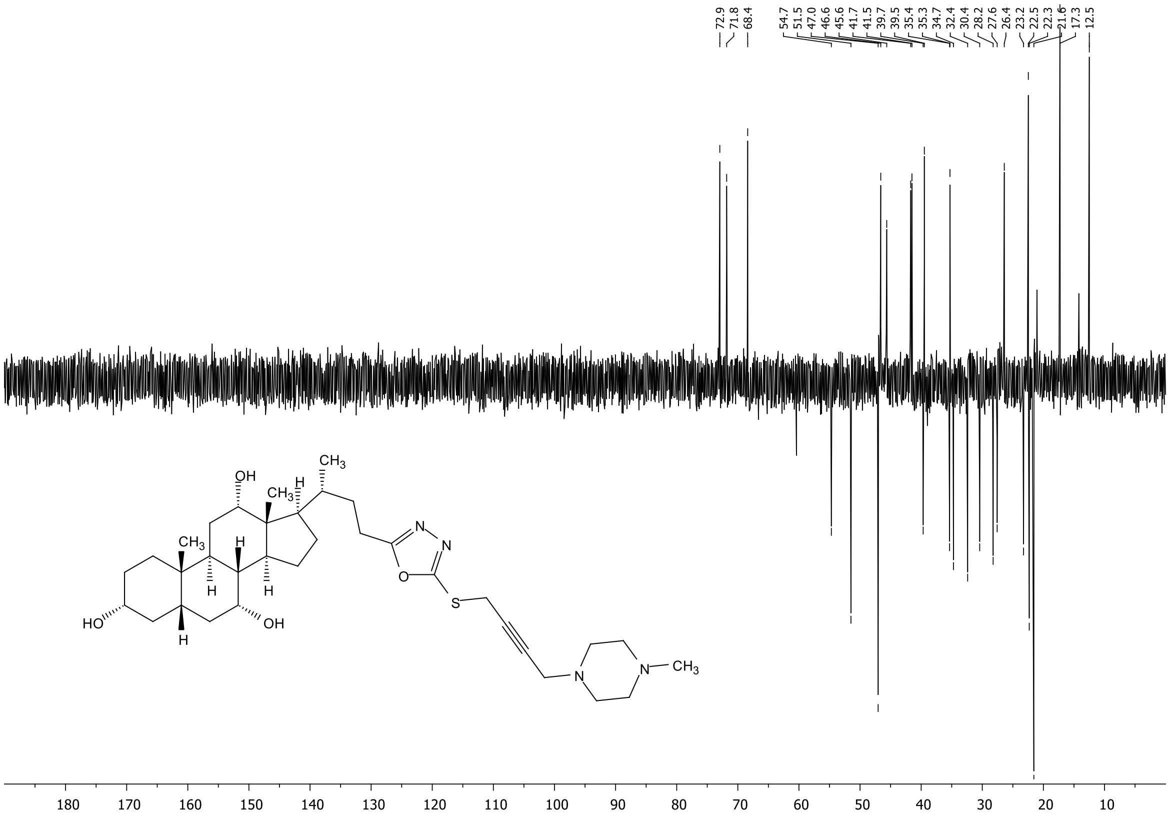
72.9
71.8
68.4
54.7
51.5
47.0
46.6
45.6
41.7
41.5
39.7
39.5
35.4
35.3
34.7
32.4
30.4
28.2
27.6
26.4
23.2
22.5
22.3
21.6
17.3
12.5
180
170
160
150
140
130
120
110
100
90
80
70
60
50
40
30
20
10

Compound 4j

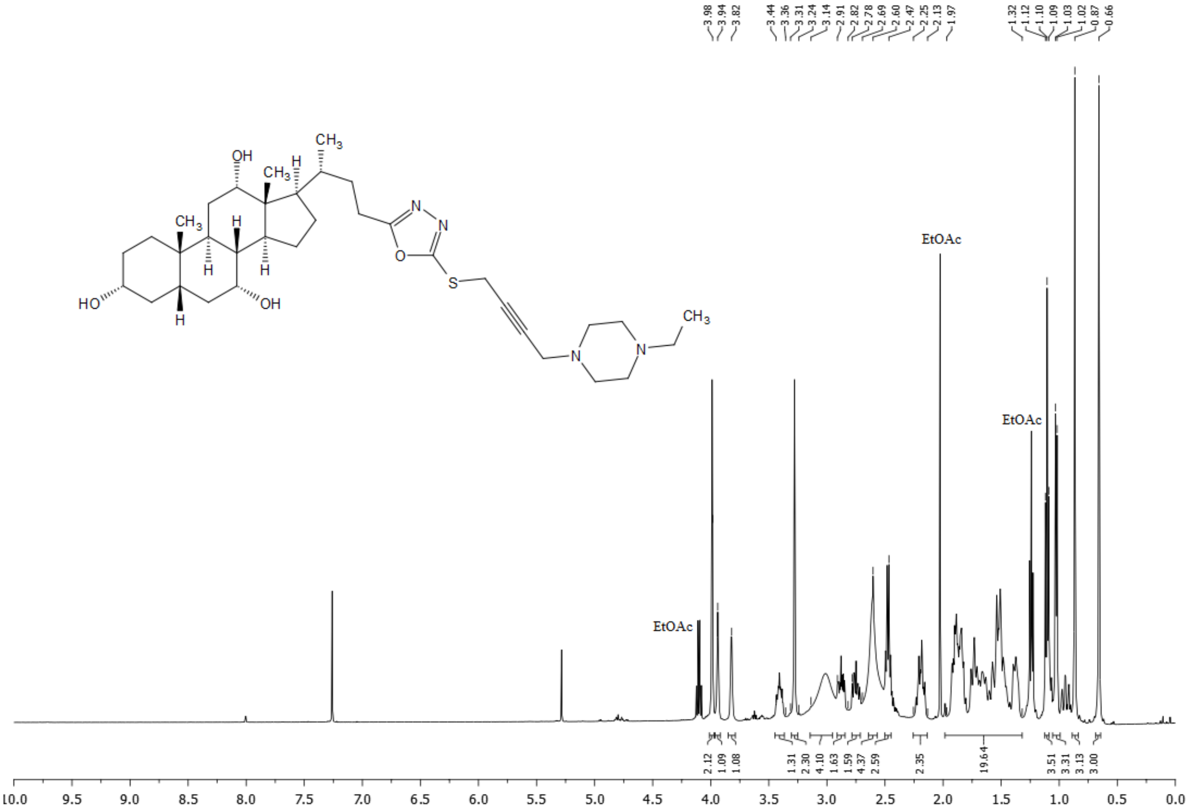
3.98
3.94
3.82
3.44
3.36
3.31
3.24
3.14
2.91
2.82
2.78
2.69
2.60
2.47
2.25
2.13
1.97
1.32
1.12
1.10
1.09
1.03
1.02
0.87
0.66
EtOAc
EtOAc
EtOAc
2.12
1.09
1.08
1.31
2.30
4.10
1.63
1.59
4.37
2.59
2.35
19.64
3.51
3.31
3.13
3.00
10.0
9.5
9.0
8.5
8.0
7.5
7.0
6.5
6.0
5.5
5.0
4.5
4.0
3.5
3.0
2.5
2.0
1.5
1.0
0.5
0.0

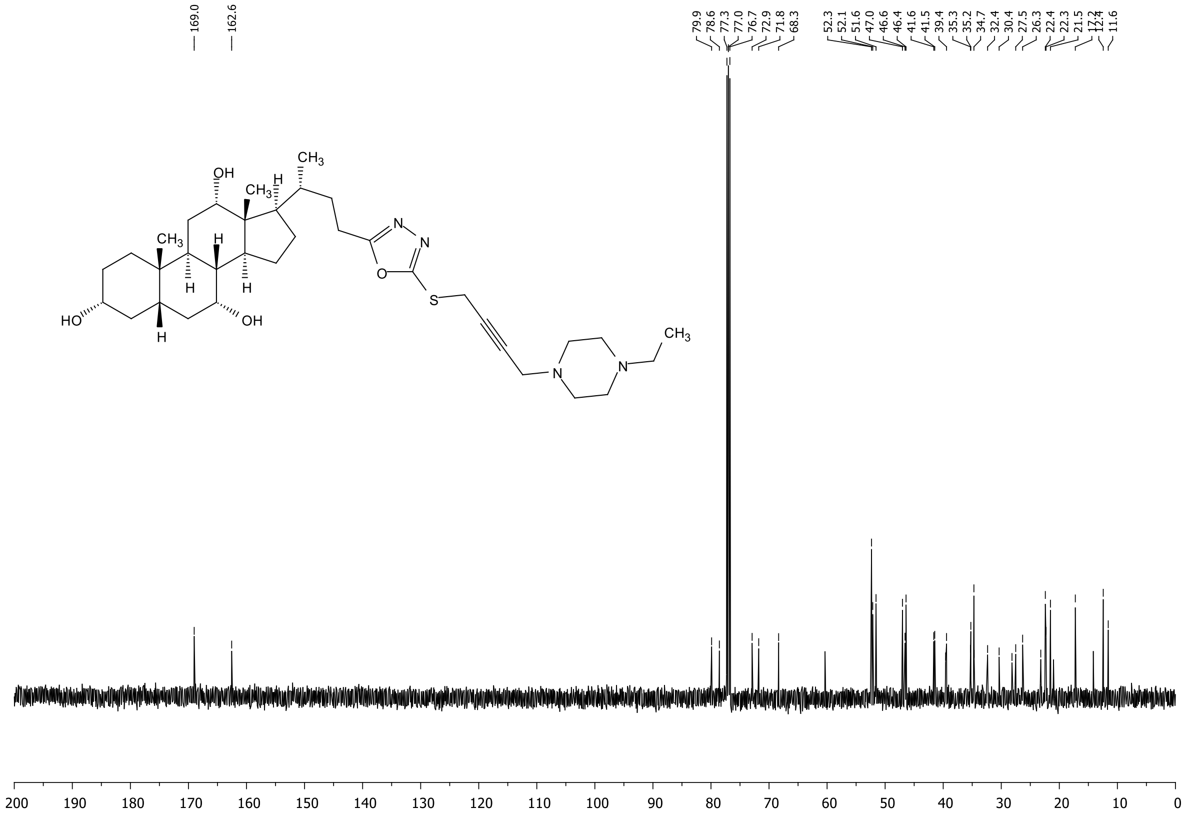
169.0
162.6
79.9
78.6
77.3
77.0
76.7
72.9
71.8
68.3
52.3
52.1
51.6
47.0
46.6
46.4
41.6
41.5
39.4
35.3
35.2
34.7
32.4
30.4
27.5
26.3
22.4
22.3
21.5
17.2
12.4
11.6
200
190
180
170
160
150
140
130
120
110
100
90
80
70
60
50
40
30
20
10
0

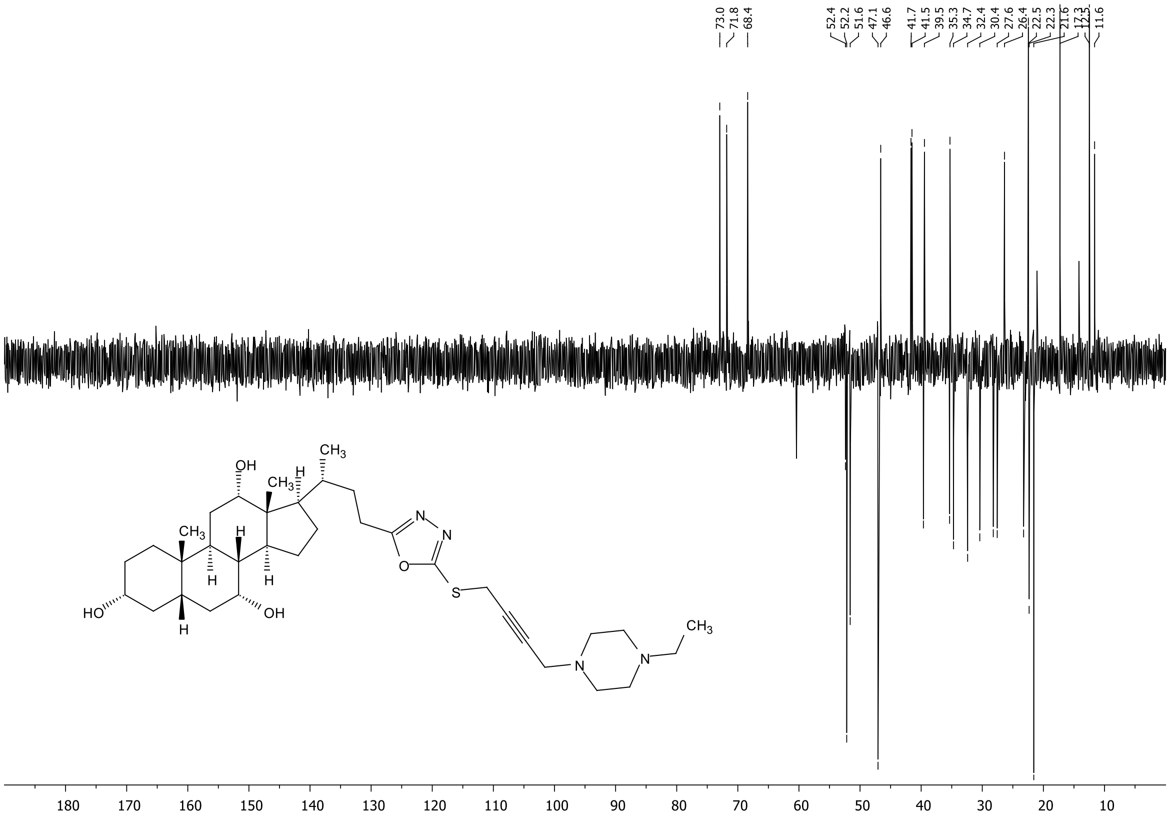
73.0
71.8
68.4
52.4
52.2
51.6
47.1
46.6
41.7
41.5
39.5
35.3
34.7
32.4
30.4
27.6
26.4
22.5
22.3
21.6
17.3
12.5
11.6
CH3
OH
CH3
H
N
N
CH3
H
H
H
H
O
S
HO
OH
H
CH3
N
N
180
170
160
150
140
130
120
110
100
90
80
70
60
50
40
30
20
10

Compound 4k

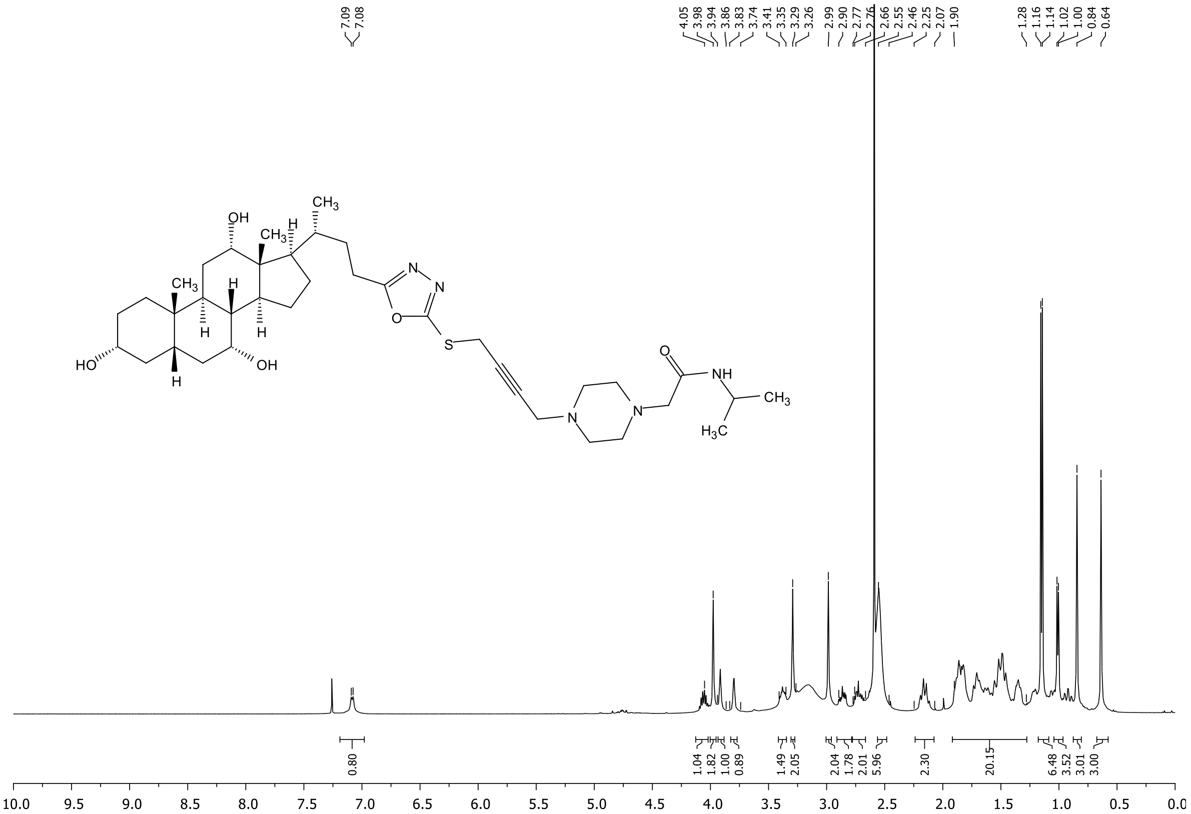

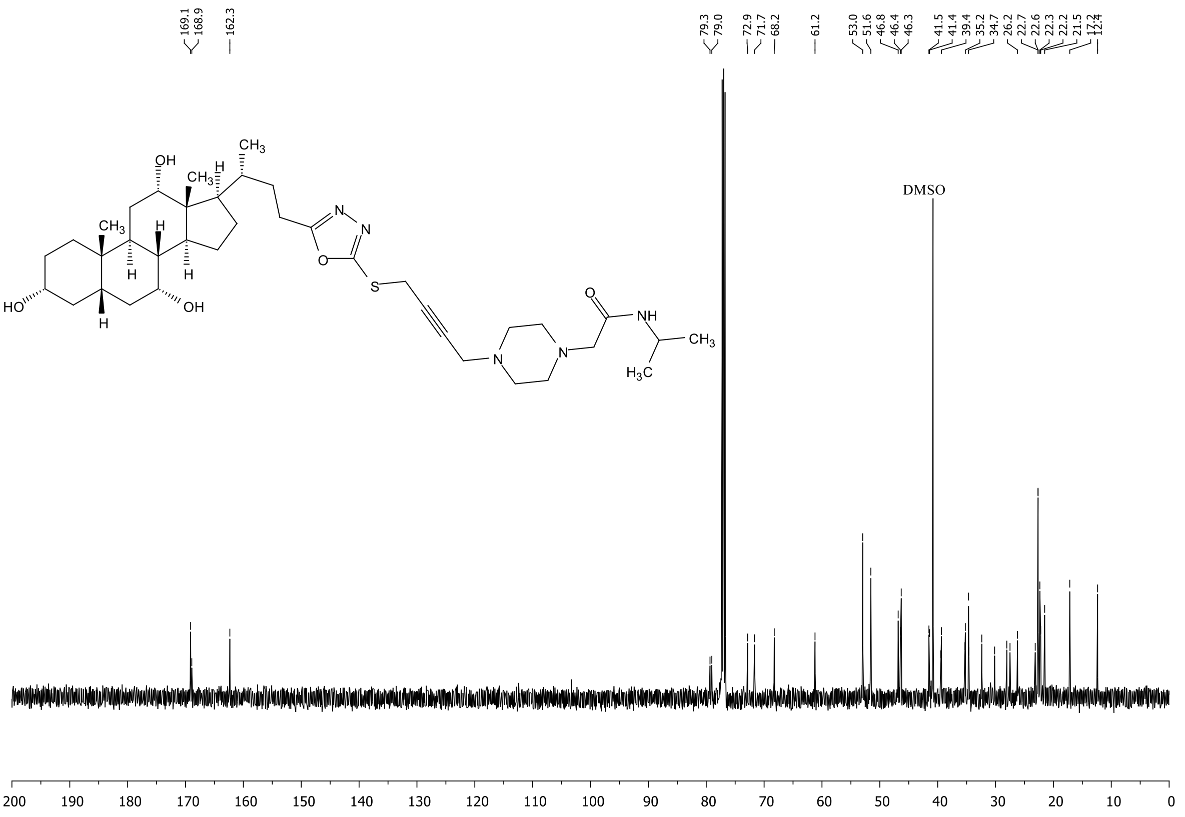

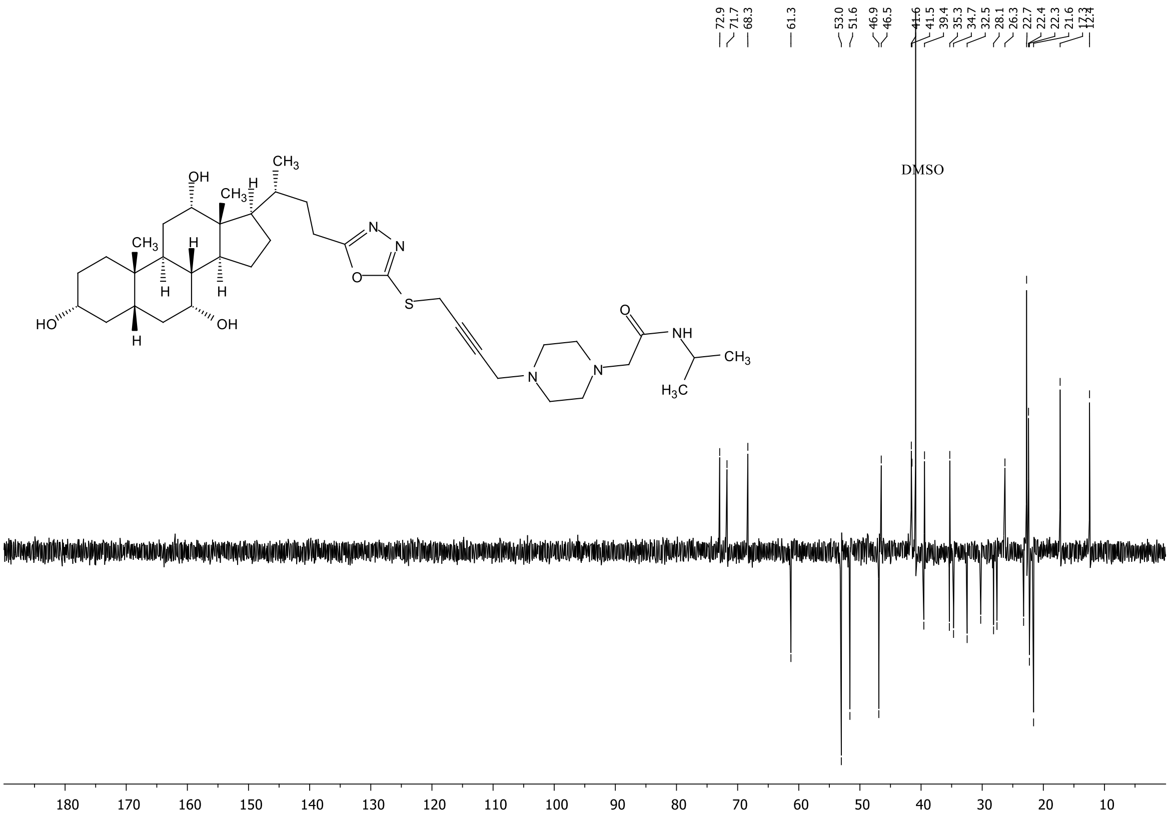
72.9
71.7
68.3
61.3
53.0
51.6
46.9
46.5
41.6
41.5
39.4
35.3
34.7
32.5
28.1
26.3
22.7
22.4
22.3
21.6
17.3
12.4
DMSO
180
170
160
150
140
130
120
110
100
90
80
70
60
50
40
30
20
10

Compound 4l

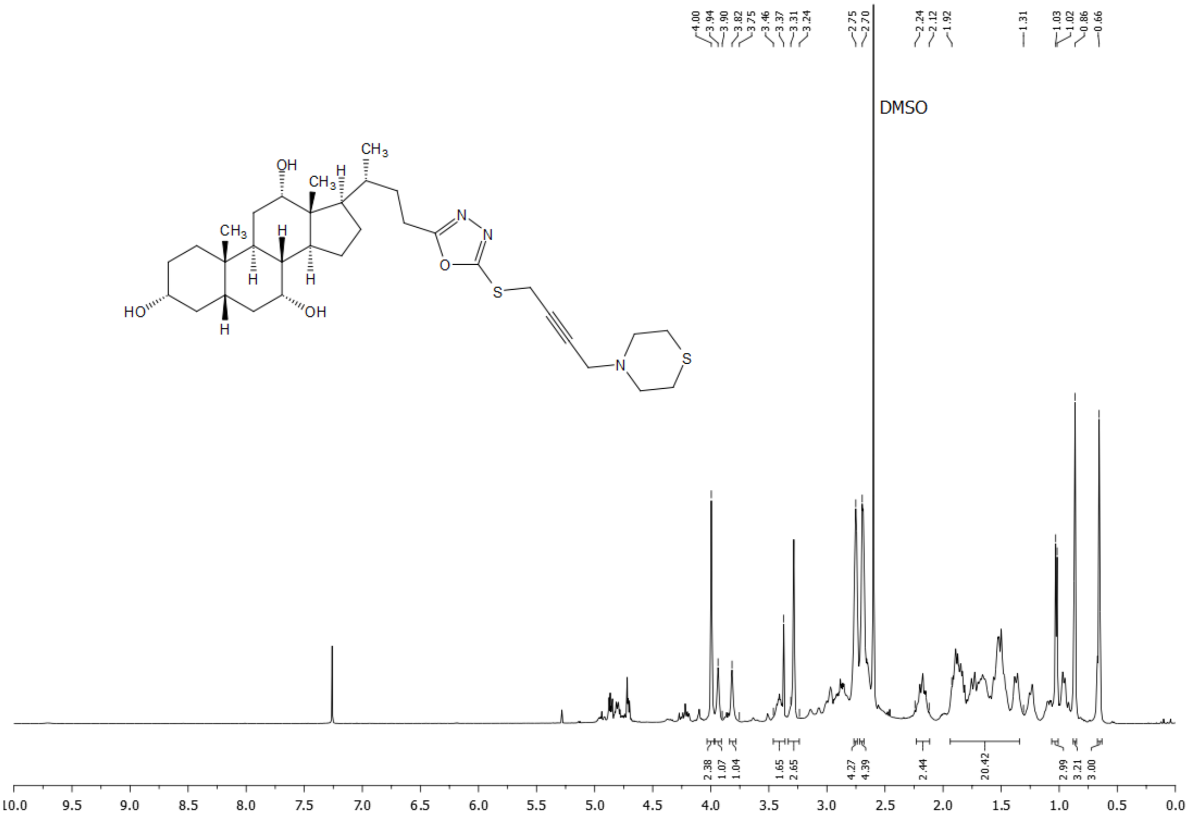
4.00
3.94
3.90
3.82
3.75
3.46
3.37
3.31
3.24
2.75
2.70
2.24
2.12
1.92
1.31
1.03
1.02
0.86
0.66
DMSO
2.38
1.07
1.04
1.65
2.65
4.27
4.39
2.44
20.42
2.99
3.21
3.00

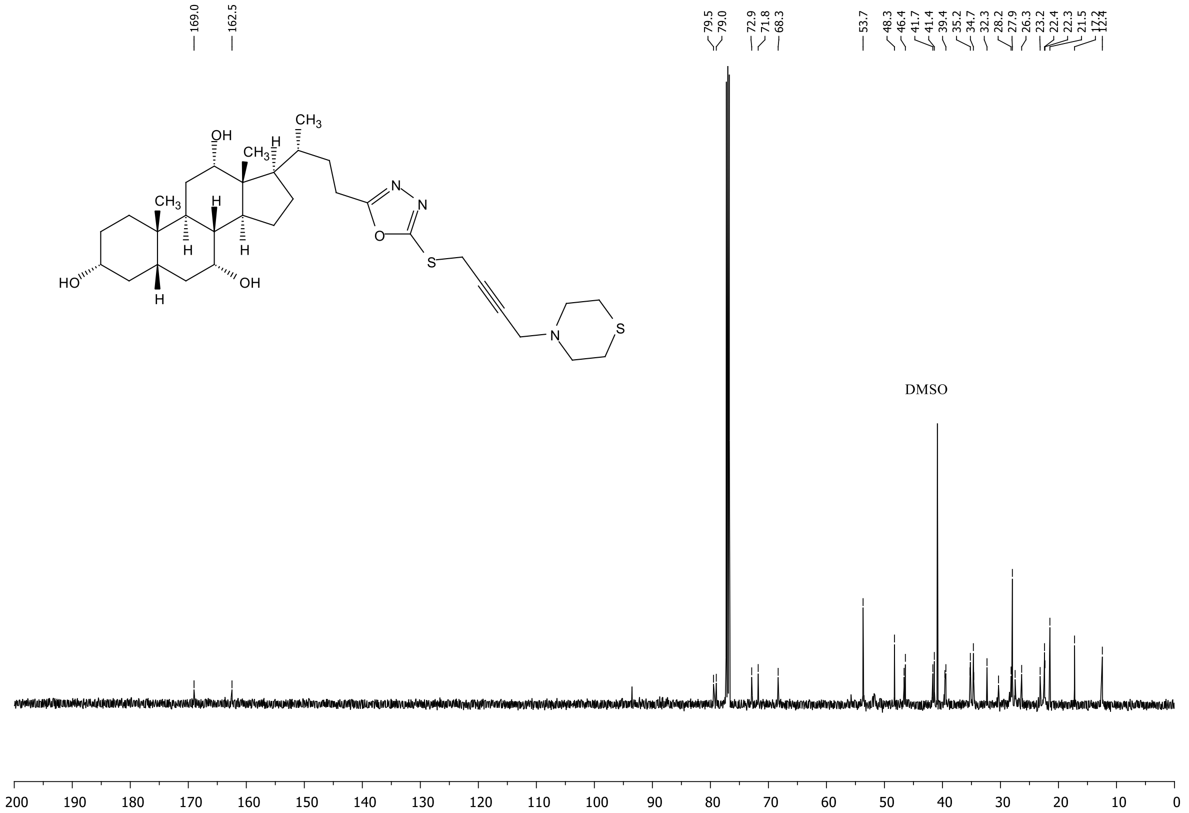
169.0
162.5
79.5
79.0
72.9
71.8
68.3
53.7
48.3
46.4
41.7
41.4
39.4
35.2
34.7
32.3
28.2
27.9
26.3
23.2
22.4
22.3
21.5
17.2
12.4
DMSO

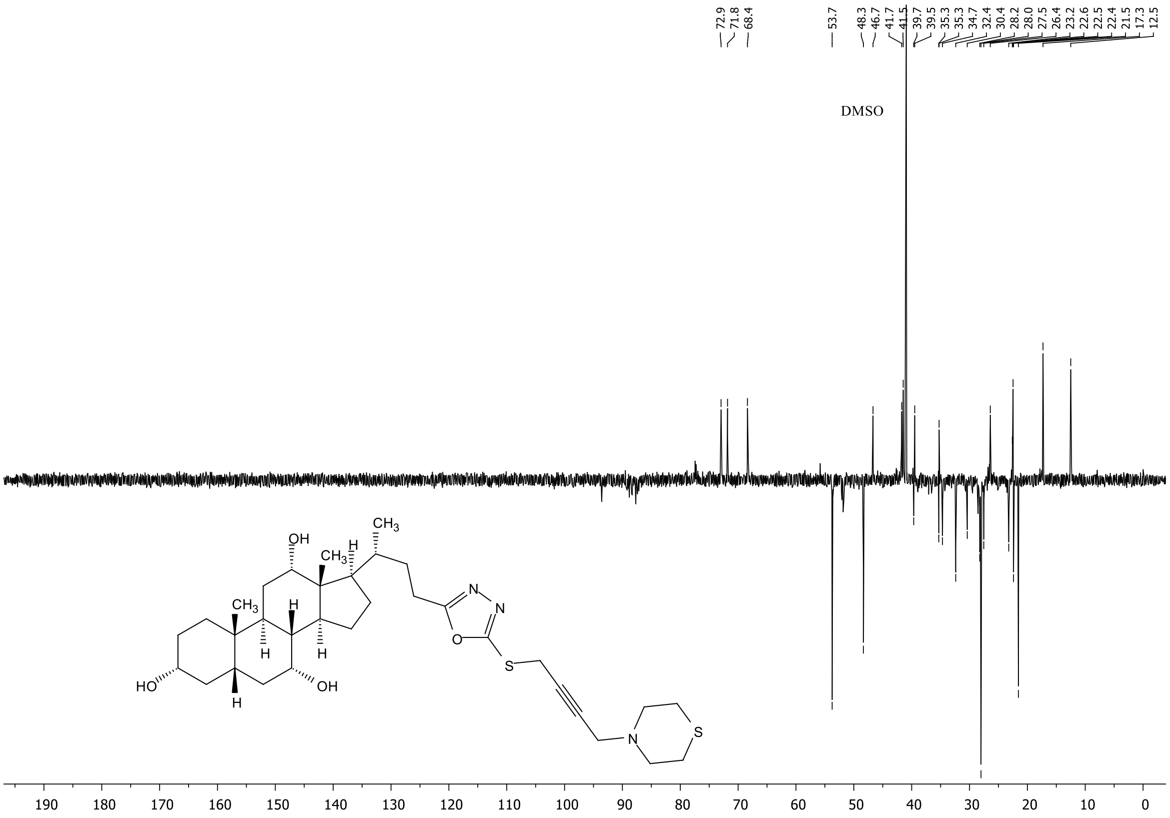

72.9
71.8
68.4
53.7
48.3
46.7
41.7
41.5
39.7
39.5
35.3
35.3
34.7
32.4
30.4
28.2
28.0
27.5
26.4
23.2
22.6
22.5
22.4
21.5
17.3
12.5
DMSO
190 180 170 160 150 140 130 120 110 100 90 80 70 60 50 40 30 20 10 0

Compound 4m

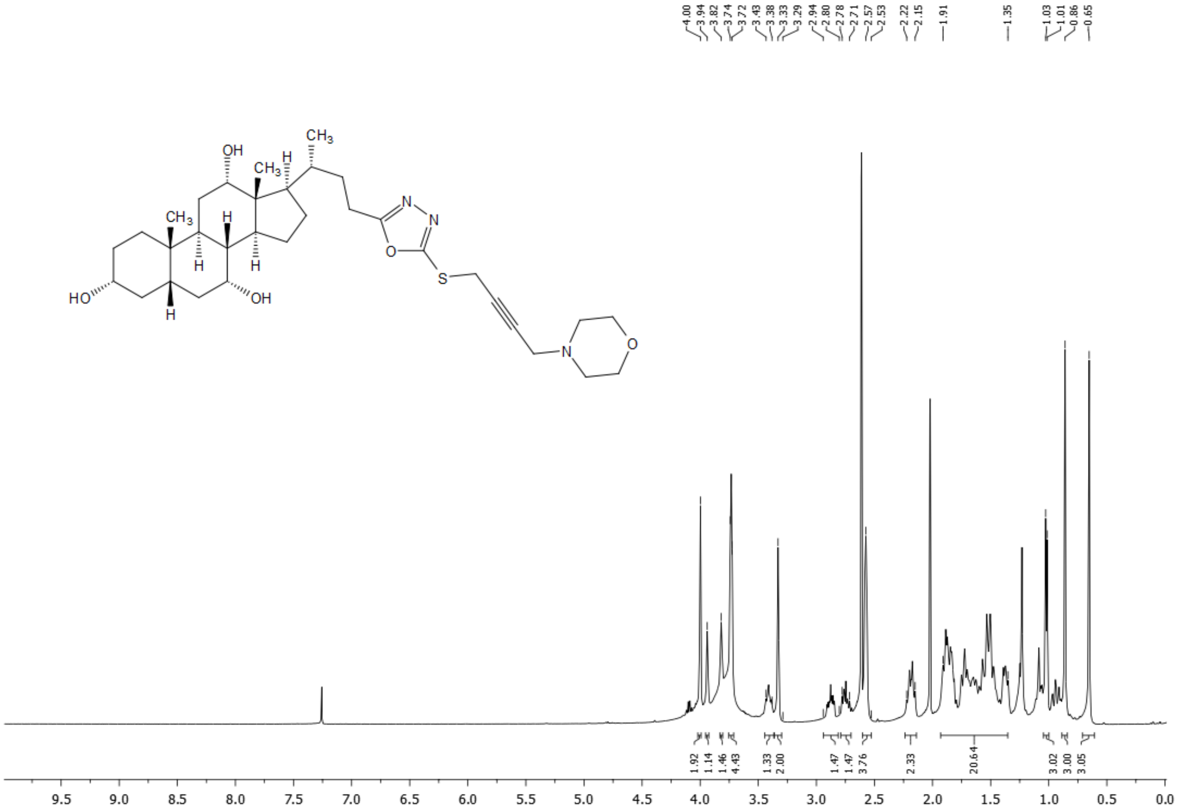

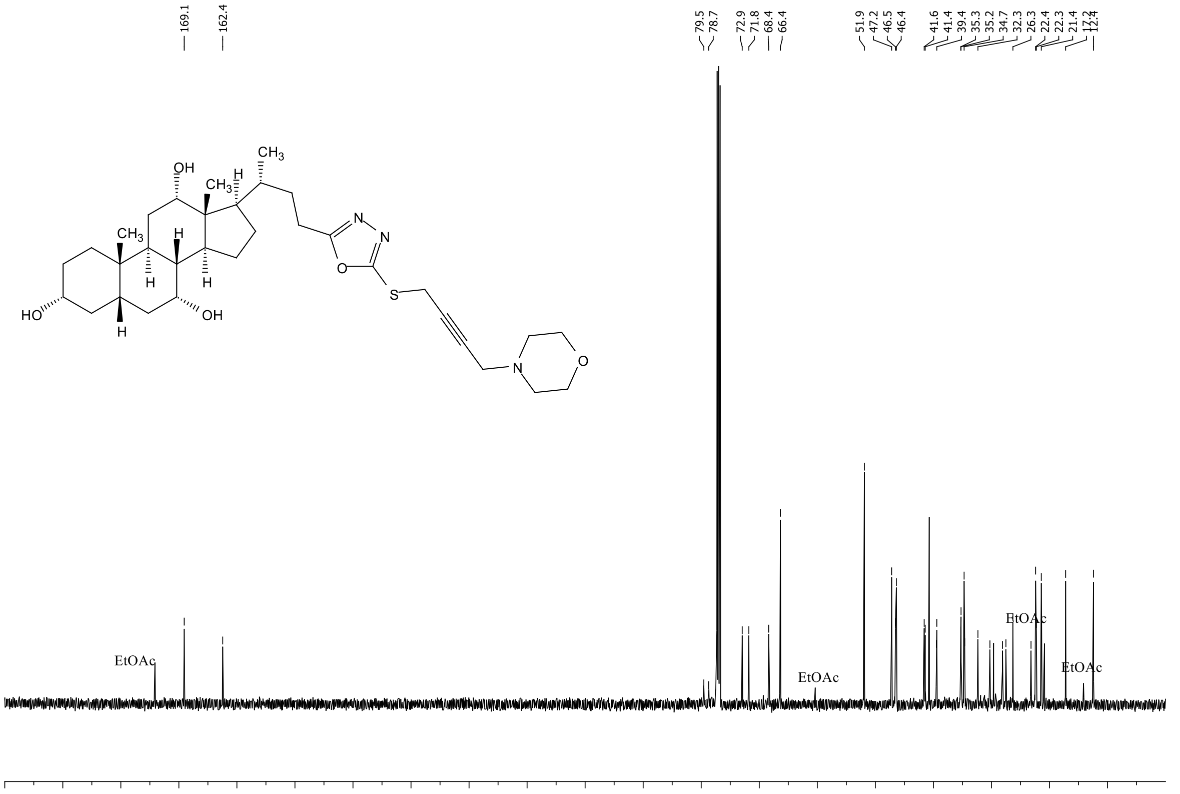

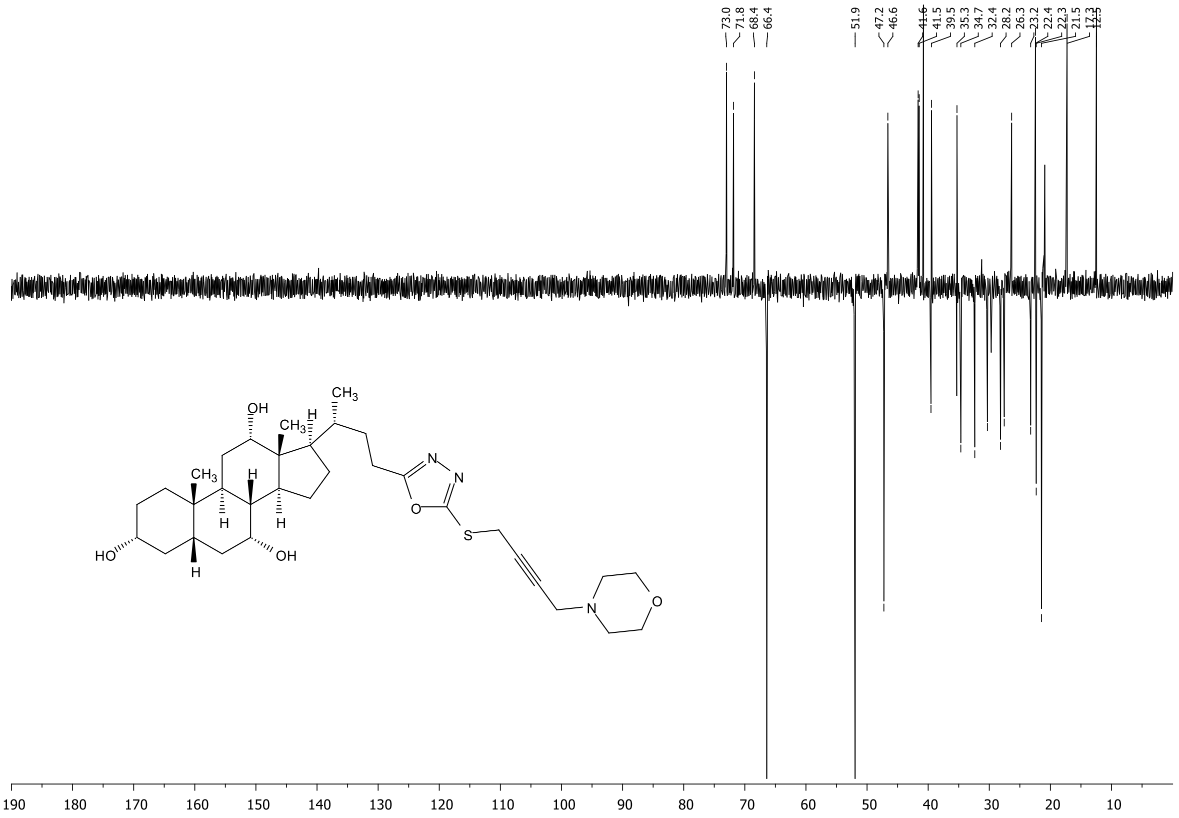
73.0
71.8
68.4
66.4
51.9
47.2
46.6
41.6
41.5
39.5
35.3
34.7
32.4
28.2
26.3
23.2
22.4
22.3
21.5
17.3
12.5
190 180 170 160 150 140 130 120 110 100 90 80 70 60 50 40 30 20 10

Compound 4n

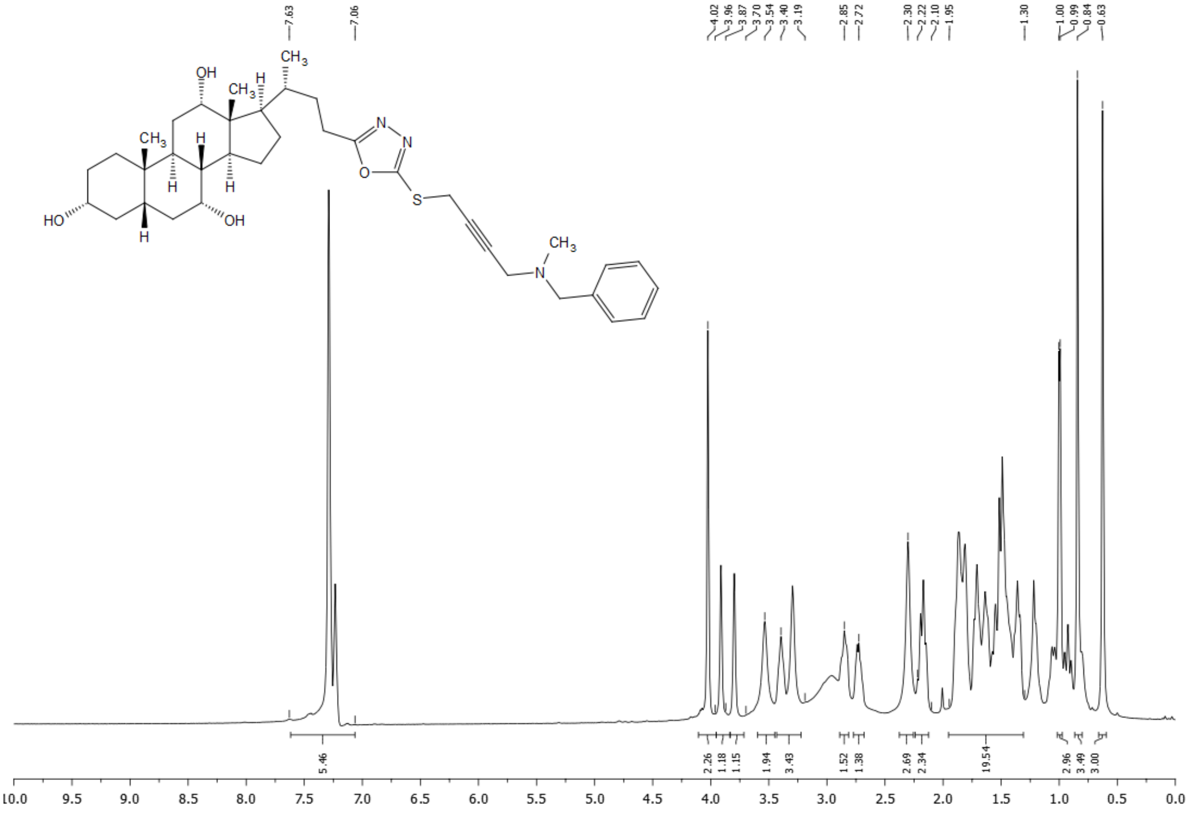

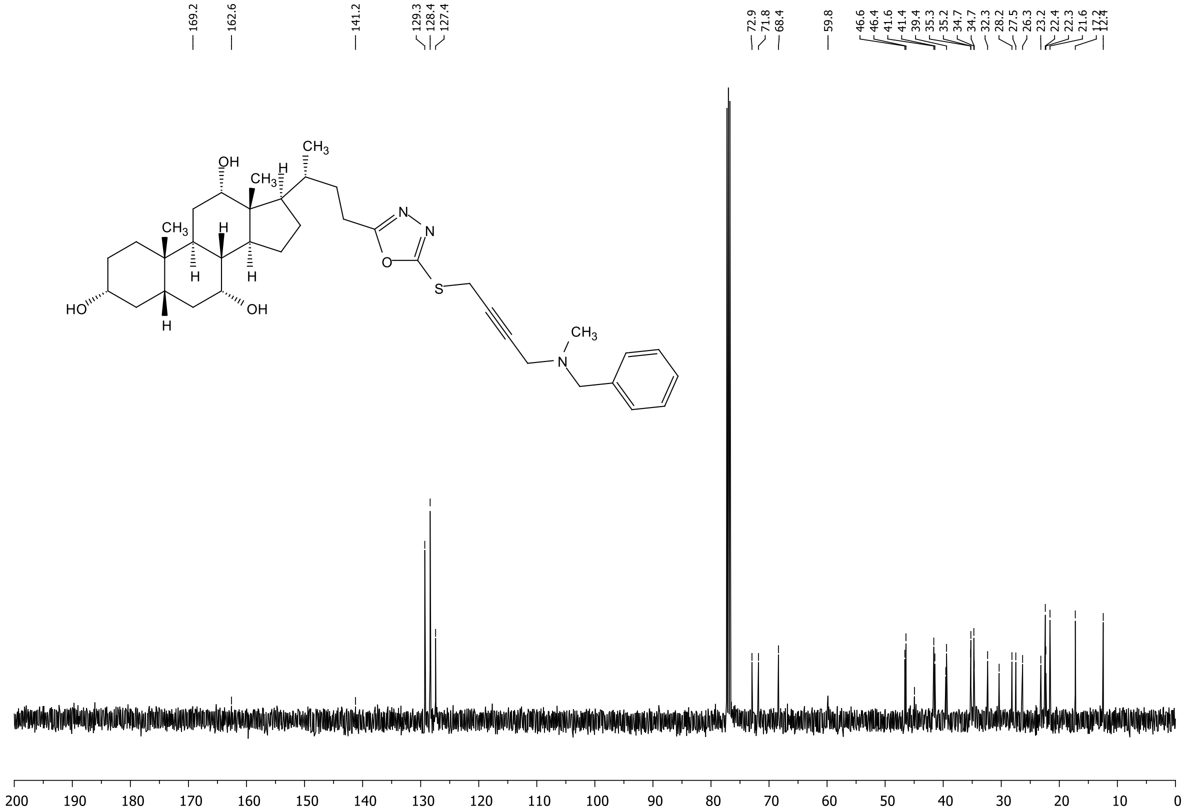

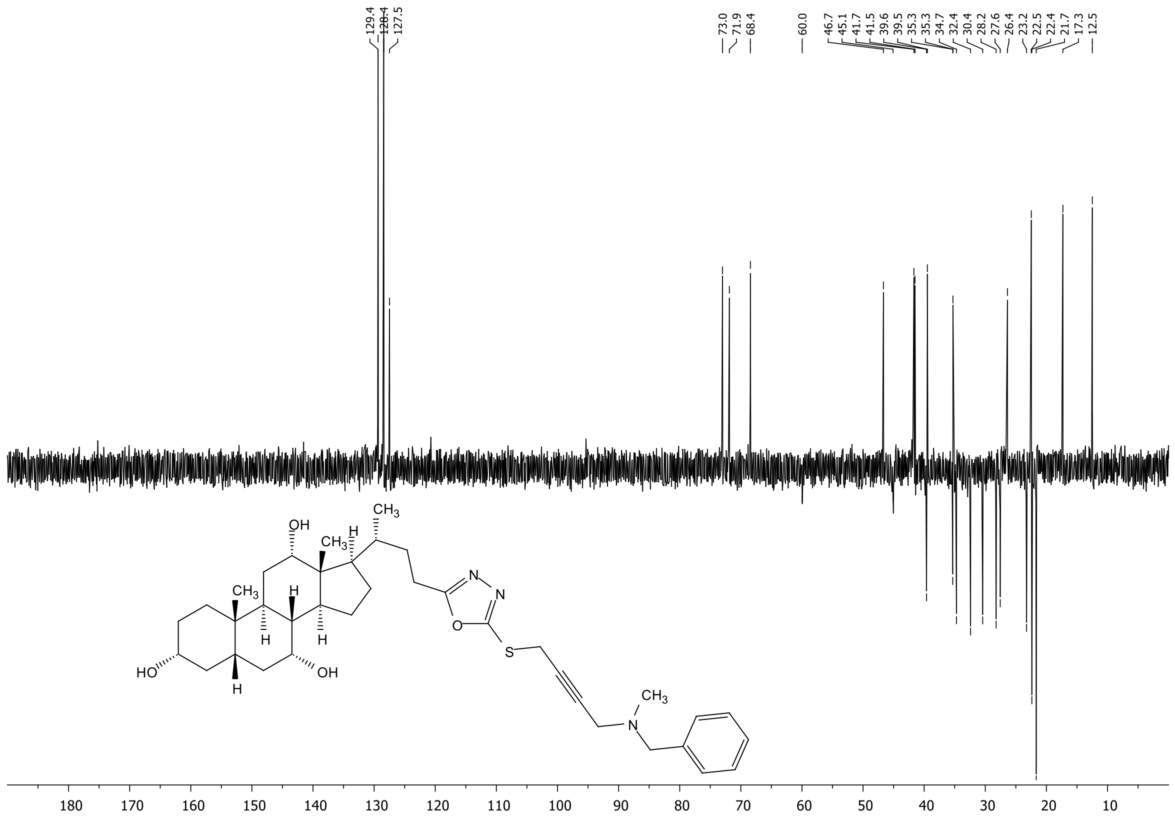
129.4
128.4
127.5
73.0
71.9
68.4
60.0
46.7
45.1
41.7
41.5
39.6
39.5
35.3
35.3
34.7
32.4
30.4
28.2
27.6
26.4
23.2
22.5
22.4
21.7
17.3
12.5
OH
CH3
H
CH3
CH3
H
H
H
HO
H
OH
N
N
O
S
CH3
N
180 170 160 150 140 130 120 110 100 90 80 70 60 50 40 30 20 10

Compound 4o

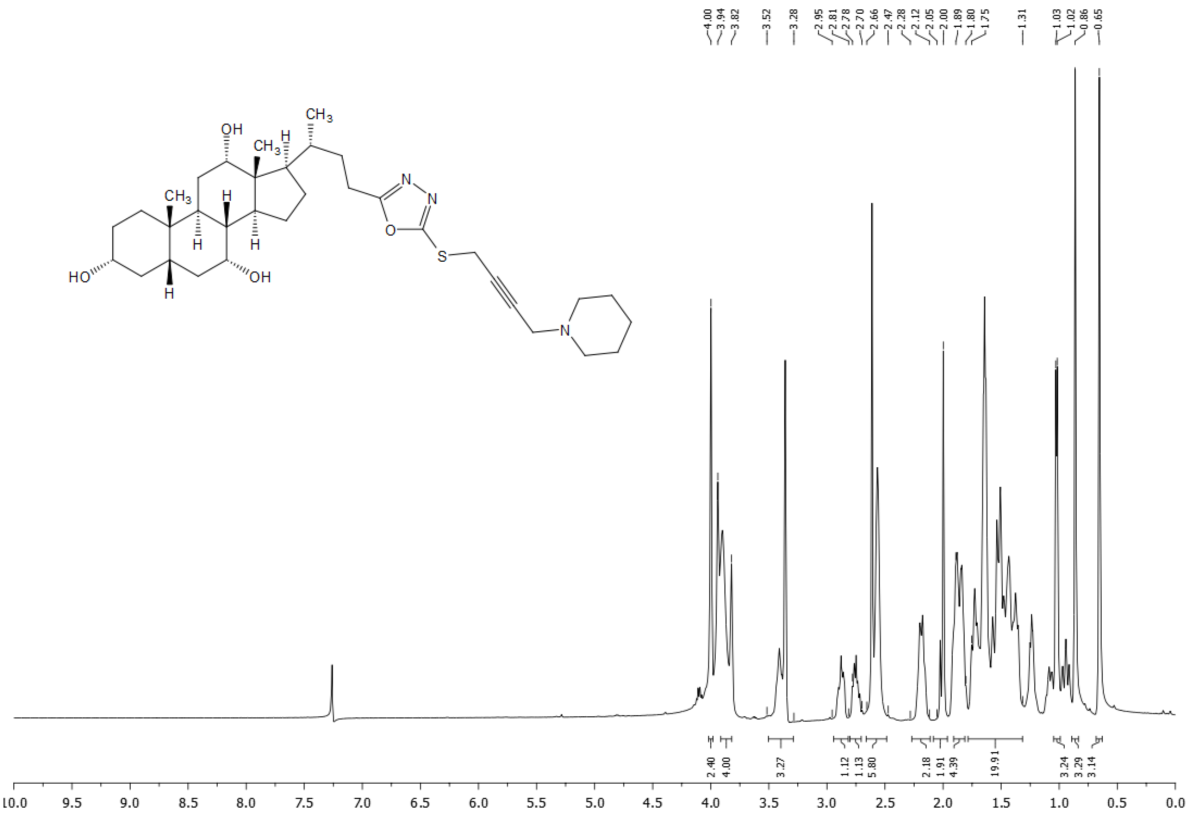

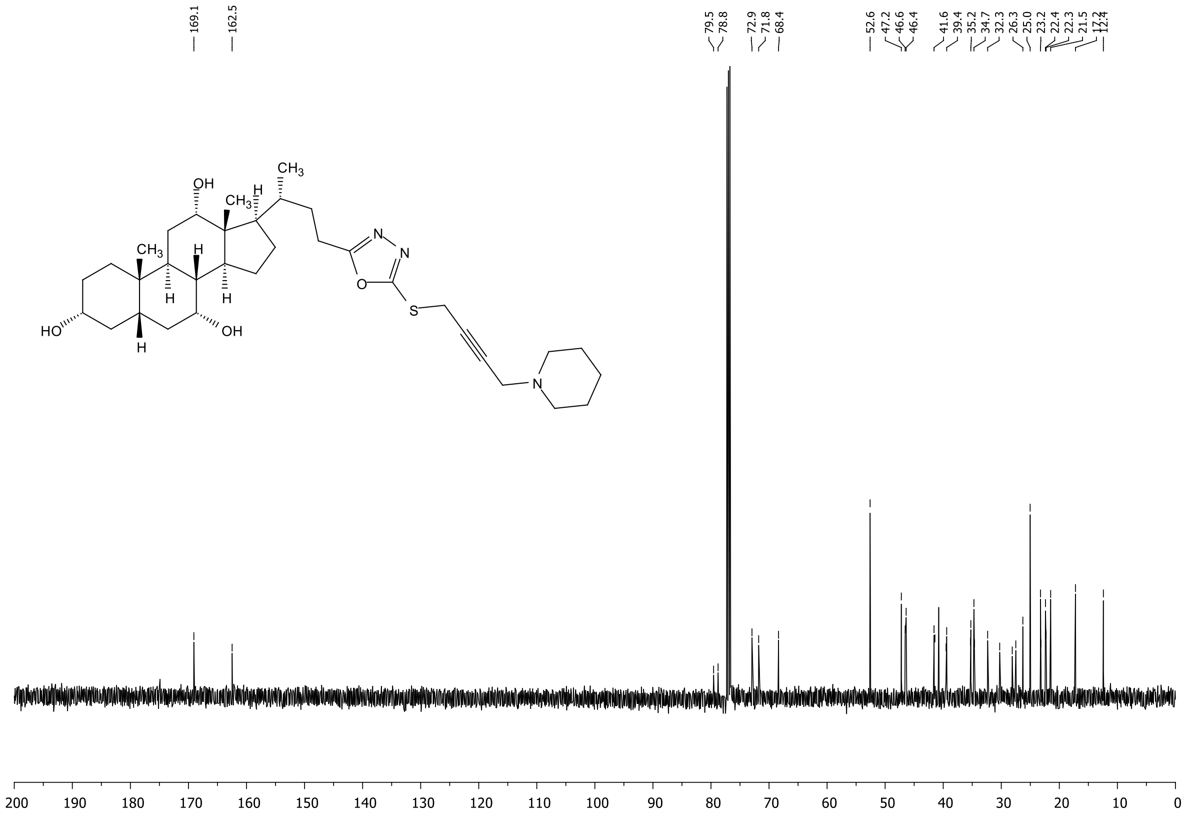

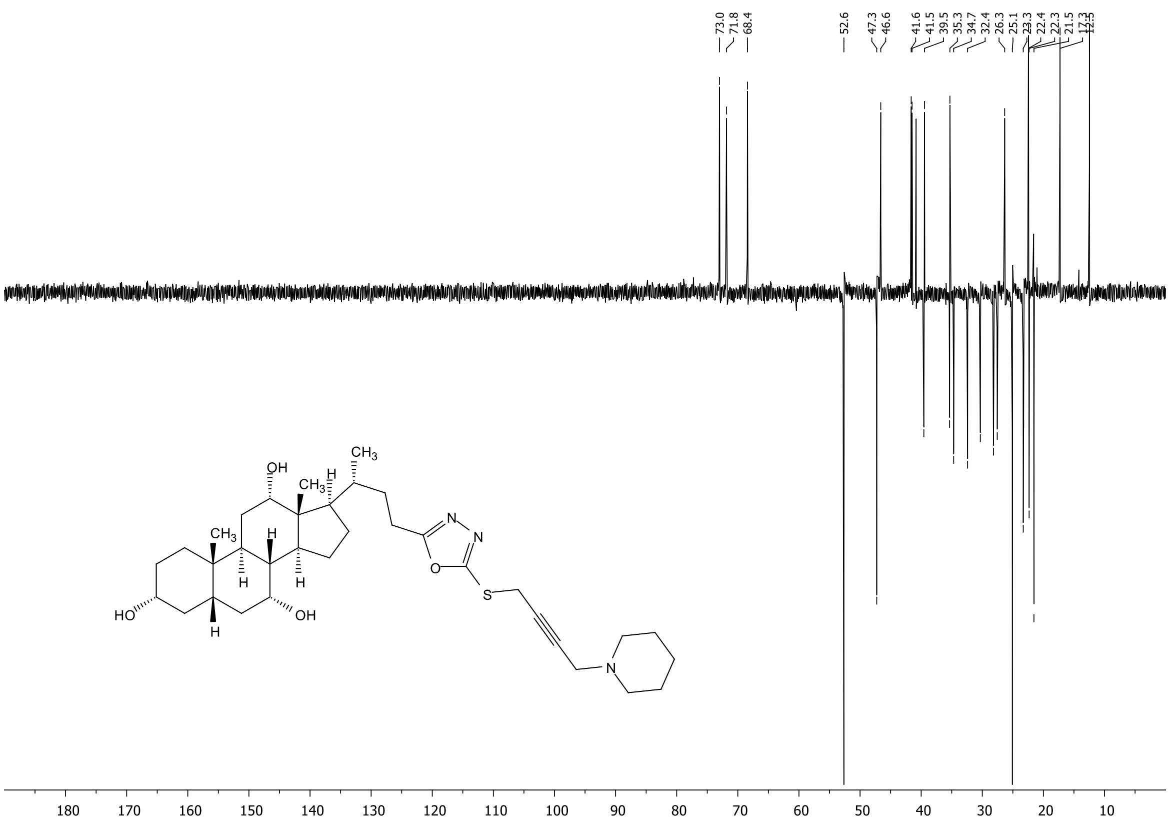

73.0
71.8
68.4
52.6
47.3
46.6
41.6
41.5
39.5
35.3
34.7
32.4
26.3
25.1
23.3
22.4
22.3
21.5
17.3
12.5
180
170
160
150
140
130
120
110
100
90
80
70
60
50
40
30
20
10
OH
CH3
H
CH3
N
N
O
S
HO
OH
N

Compound 4p

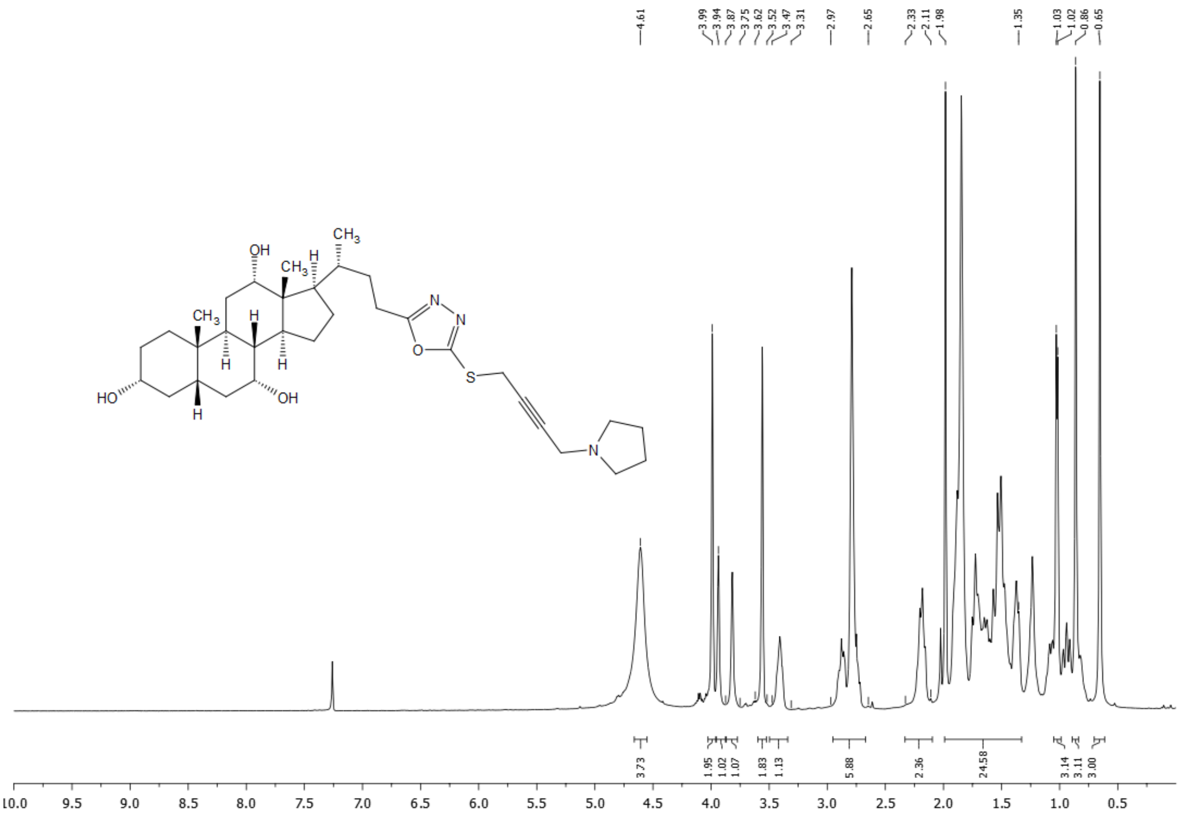

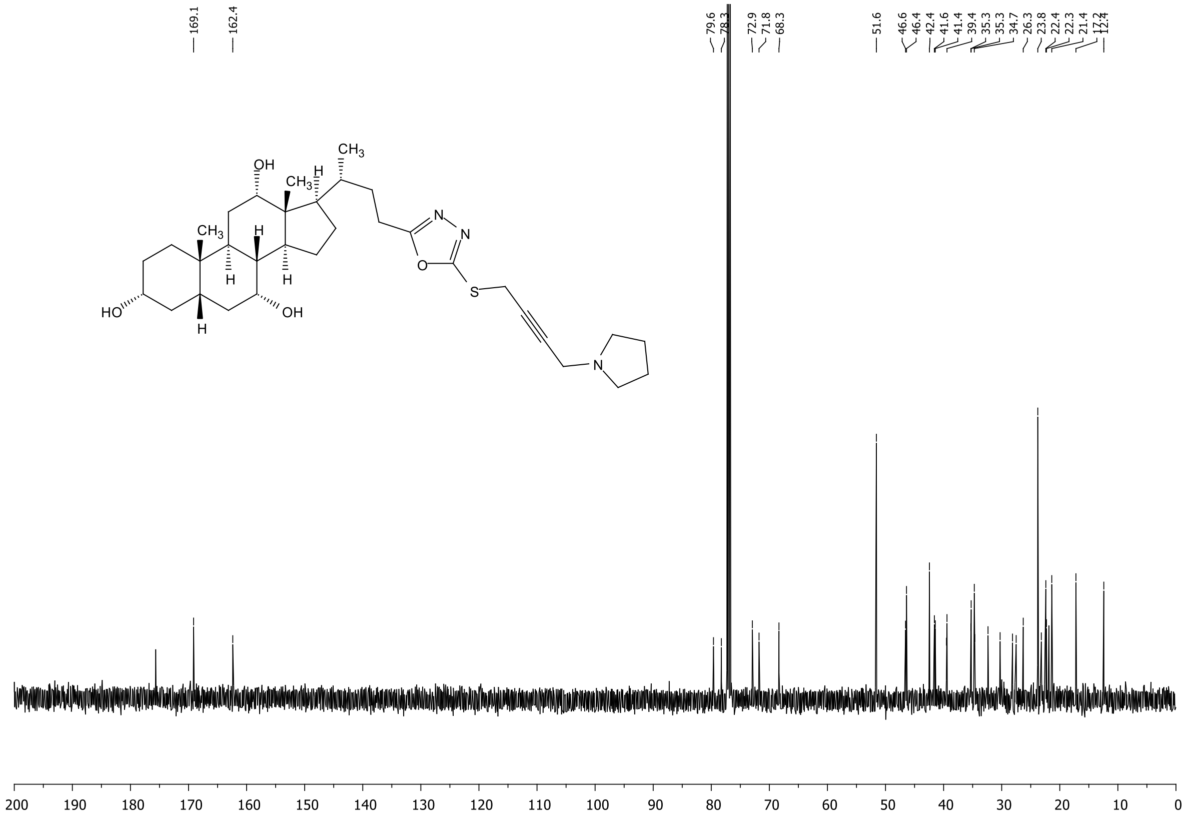

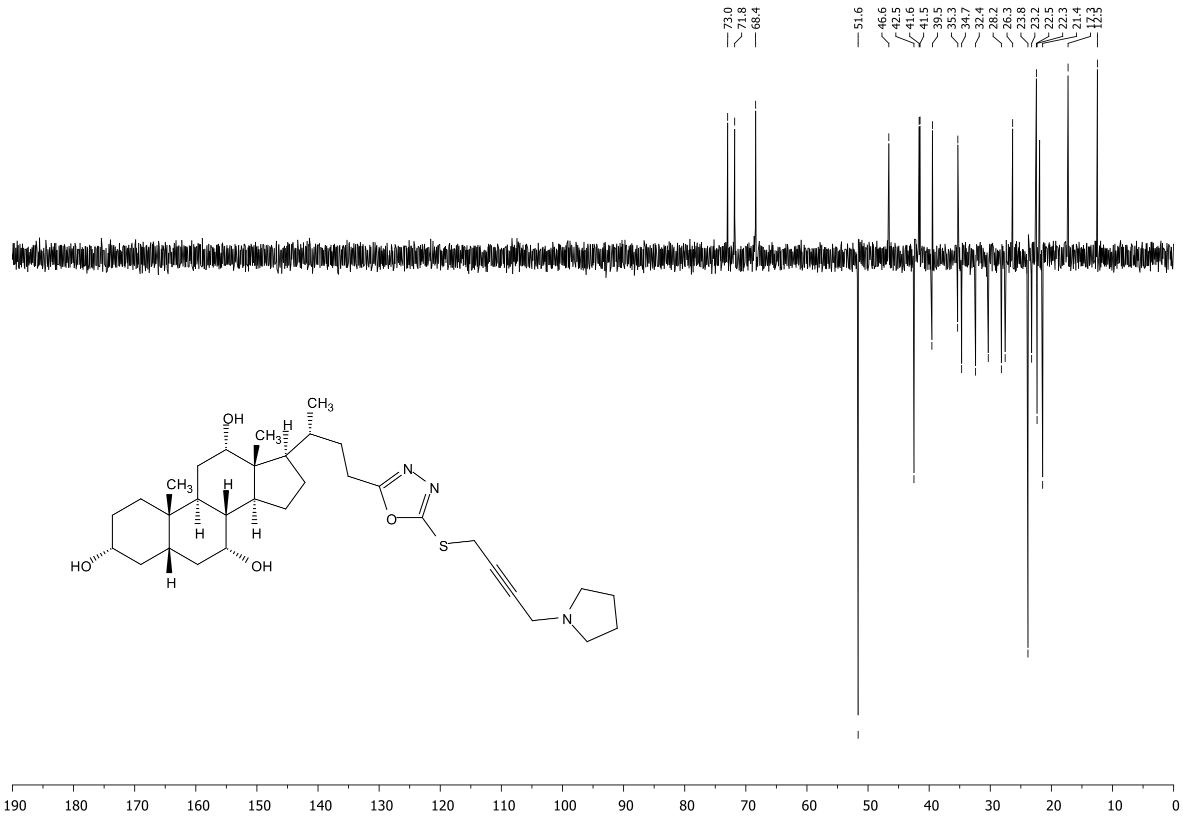
73.0
71.8
68.4
51.6
46.6
42.5
41.6
41.5
39.5
35.3
34.7
32.4
28.2
26.3
23.8
23.2
22.5
22.3
21.4
17.3
12.5
OH
CH3
H
HO
OH
N
O
S
190 180 170 160 150 140 130 120 110 100 90 80 70 60 50 40 30 20 10 0

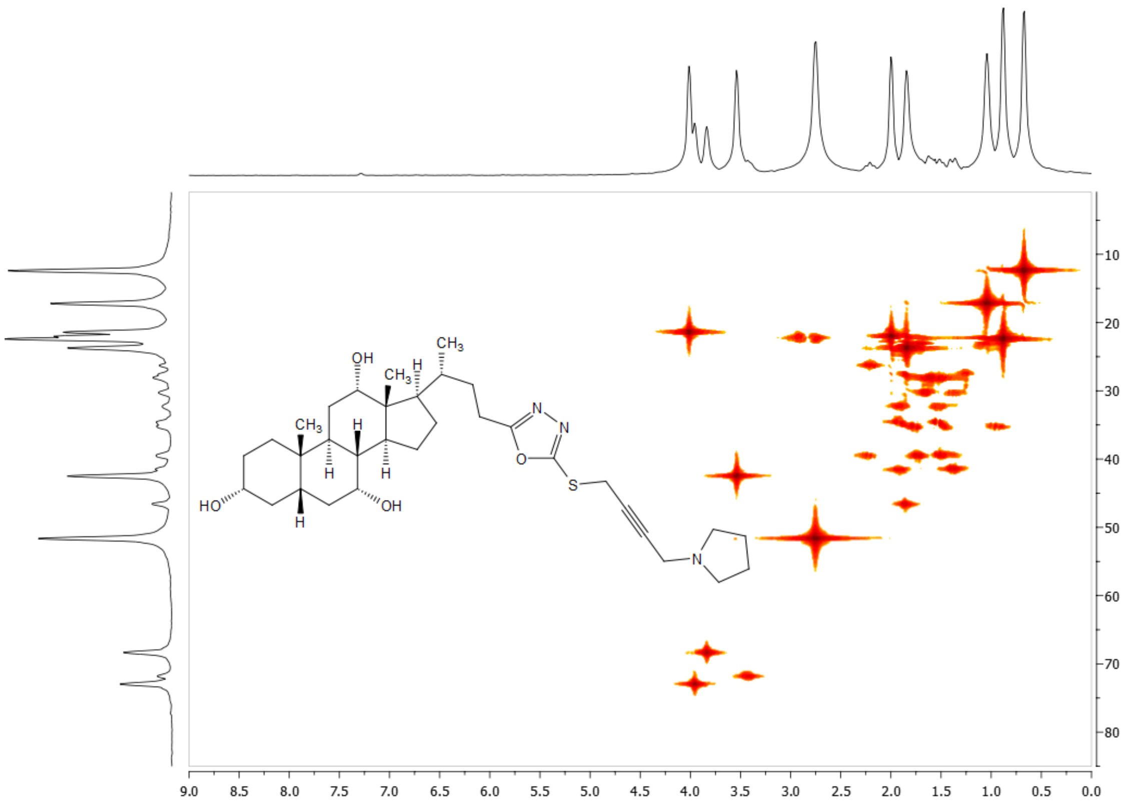
OH
CH3
H
HO
OH
N
O
S
9.0 8.5 8.0 7.5 7.0 6.5 6.0 5.5 5.0 4.5 4.0 3.5 3.0 2.5 2.0 1.5 1.0 0.5 0.0
10 20 30 40 50 60 70 80

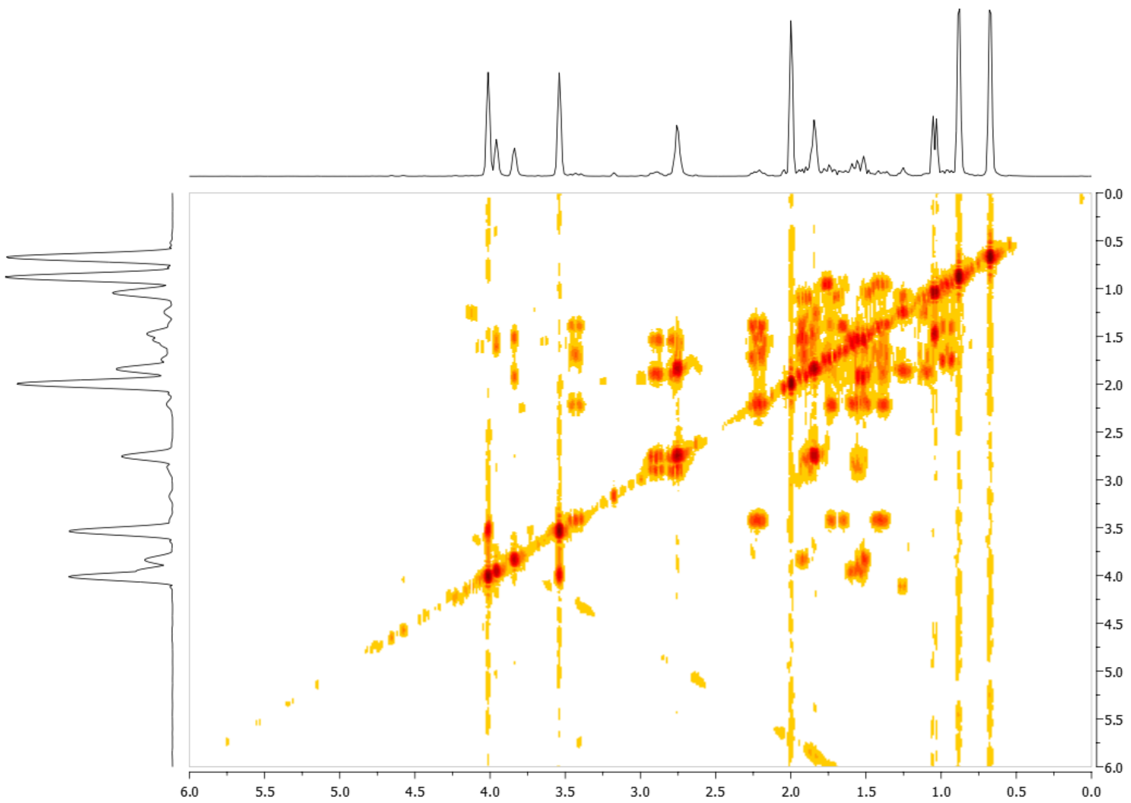
6.0
5.5
5.0
4.5
4.0
3.5
3.0
2.5
2.0
1.5
1.0
0.5
0.0

Compound 4q

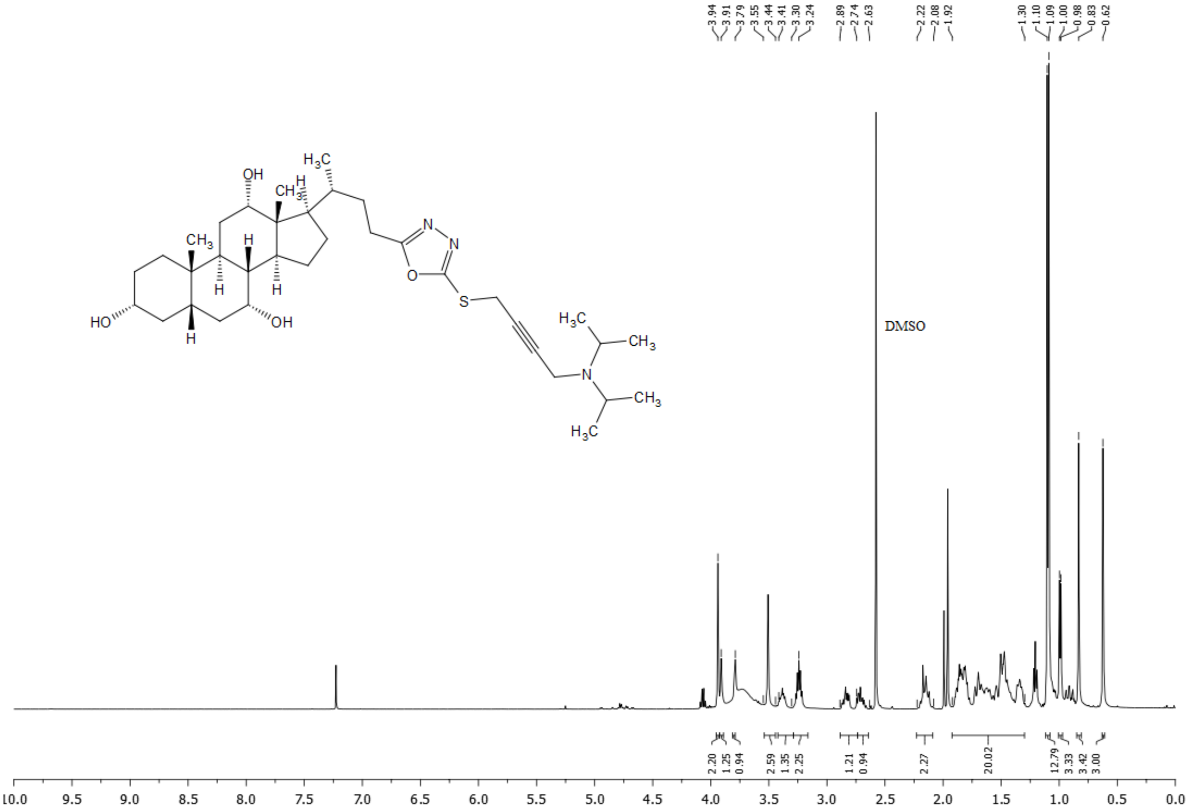

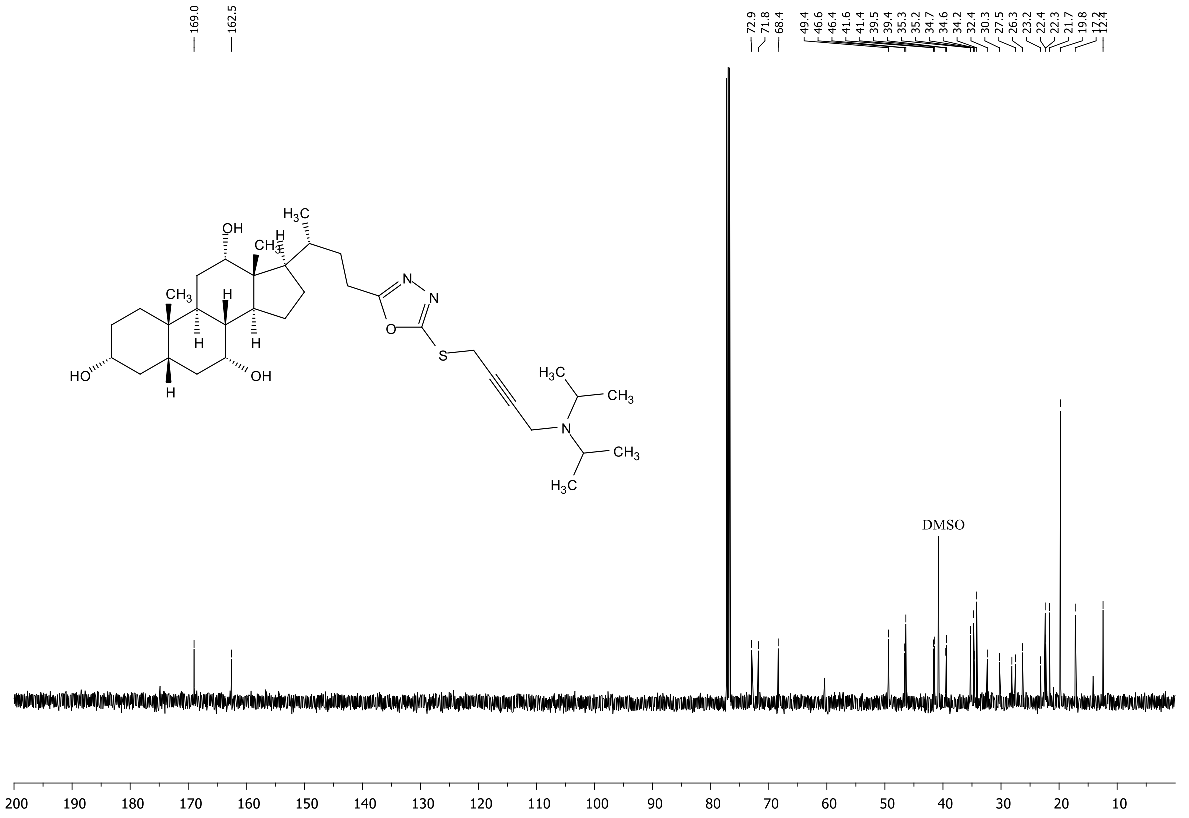

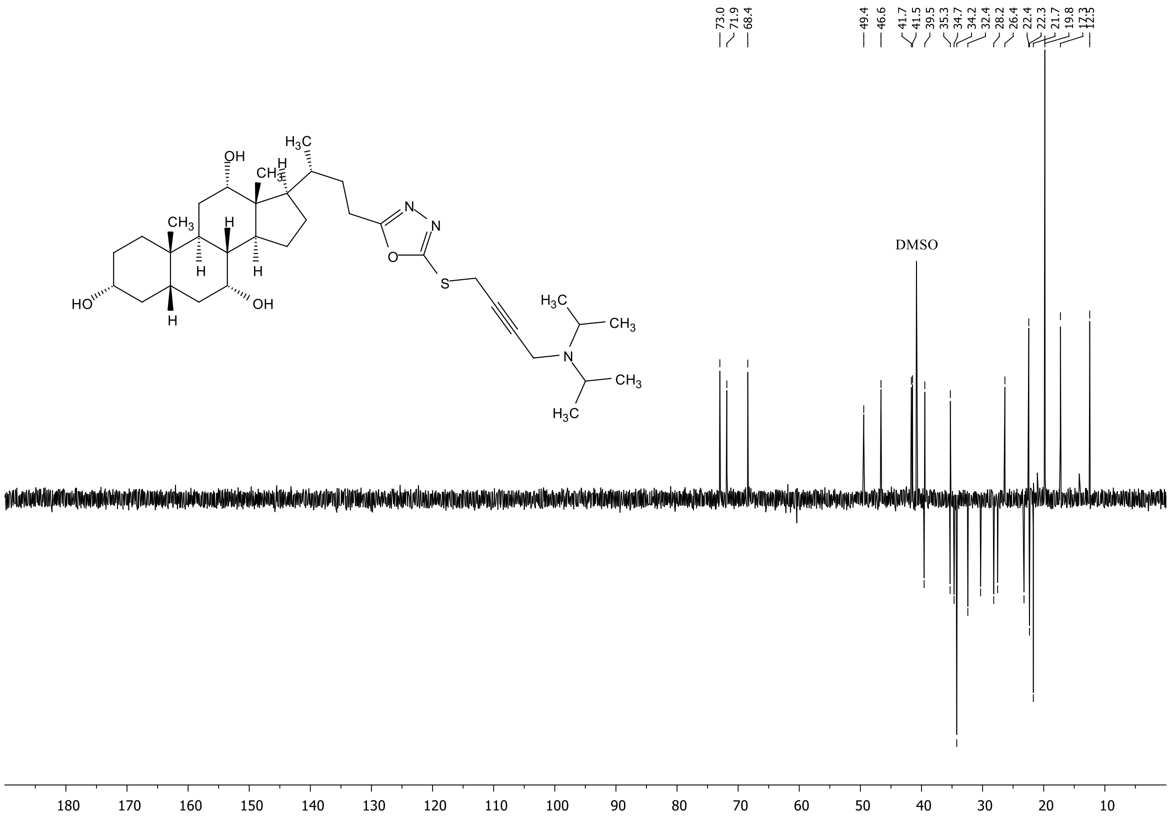

73.0
71.9
68.4
49.4
46.6
41.7
41.5
39.5
35.3
34.7
34.2
32.4
28.2
26.4
22.4
22.3
21.7
19.8
17.3
12.5
DMSO
OH
HO
H
CH3
H3C
N
O
S
180
170
160
150
140
130
120
110
100
90
80
70
60
50
40
30
20
10

Compound 4r

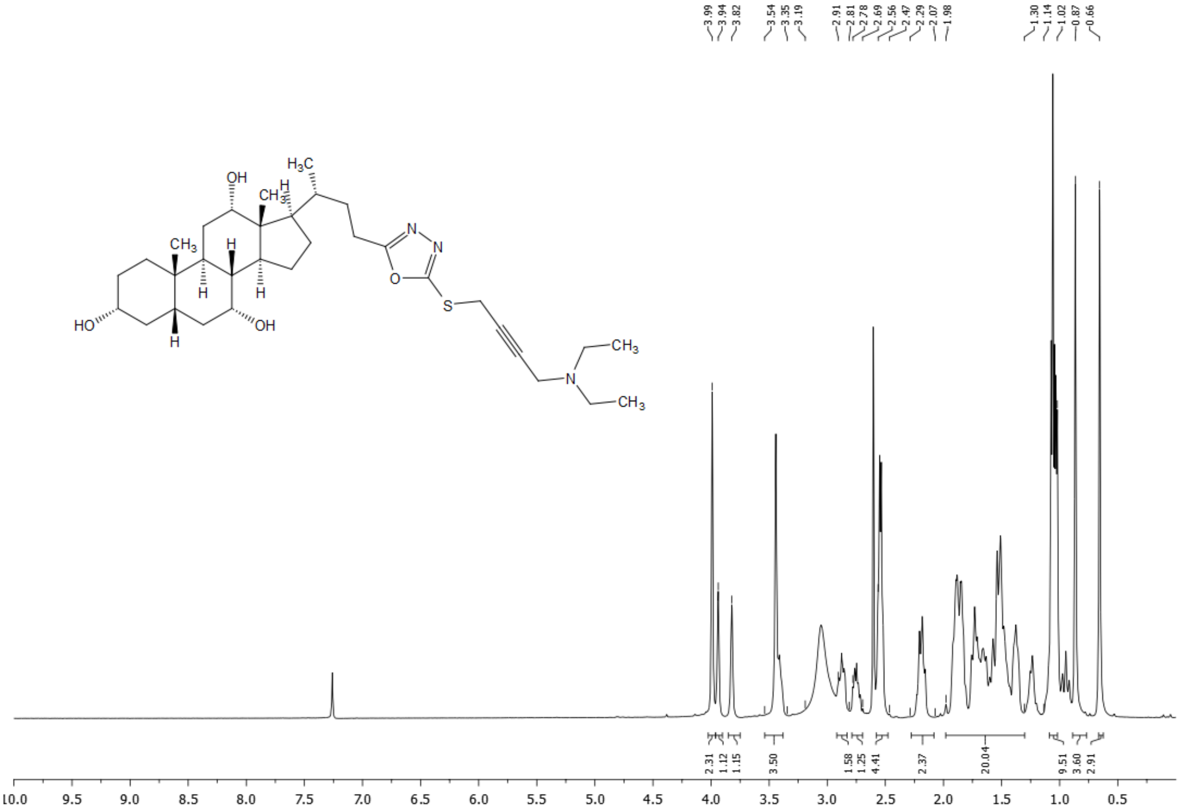

3.99
3.94
3.82
3.54
3.35
3.19
2.91
2.81
2.78
2.69
2.56
2.47
2.29
2.07
1.98
1.30
1.14
1.02
0.87
0.66
2.31
1.12
1.15
3.50
1.58
1.25
4.41
2.37
20.04
9.51
3.60
2.91

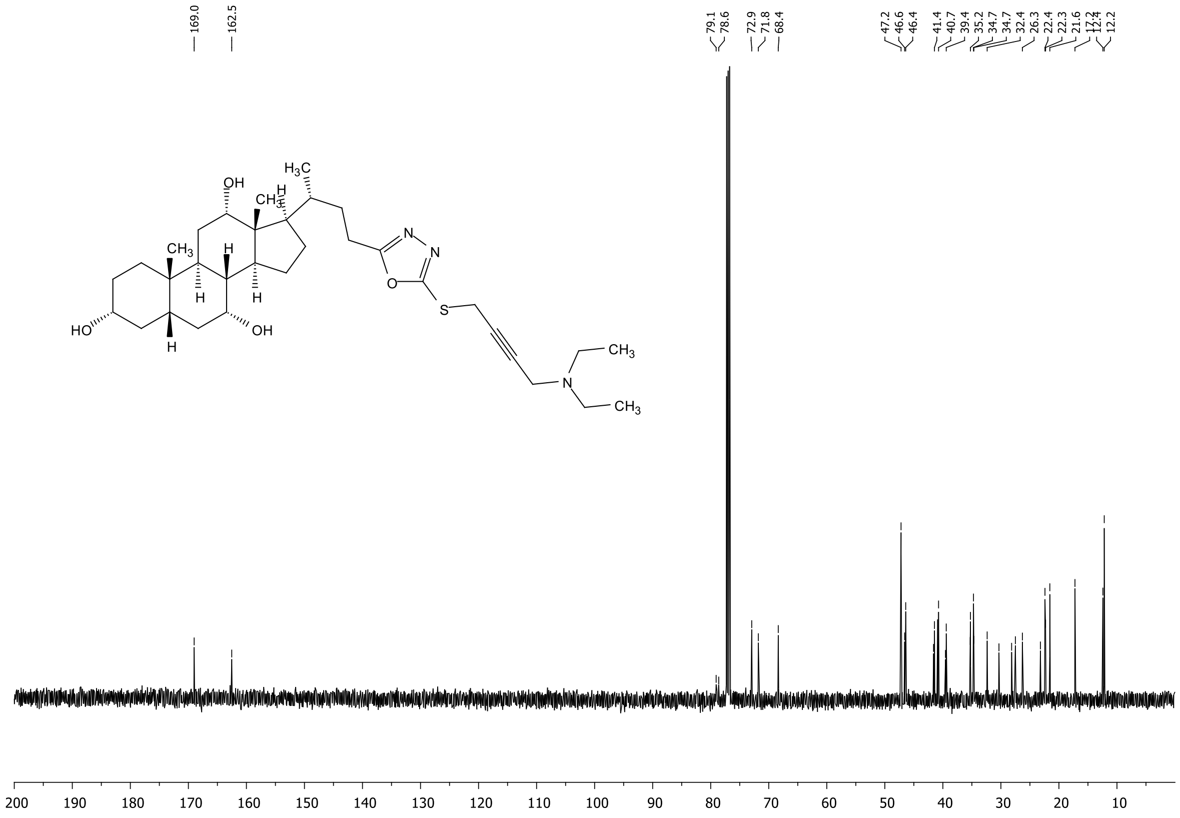

169.0
162.5
79.1
78.6
72.9
71.8
68.4
47.2
46.6
46.4
41.4
40.7
39.4
35.2
34.7
34.7
32.4
26.3
22.4
22.3
21.6
12.2

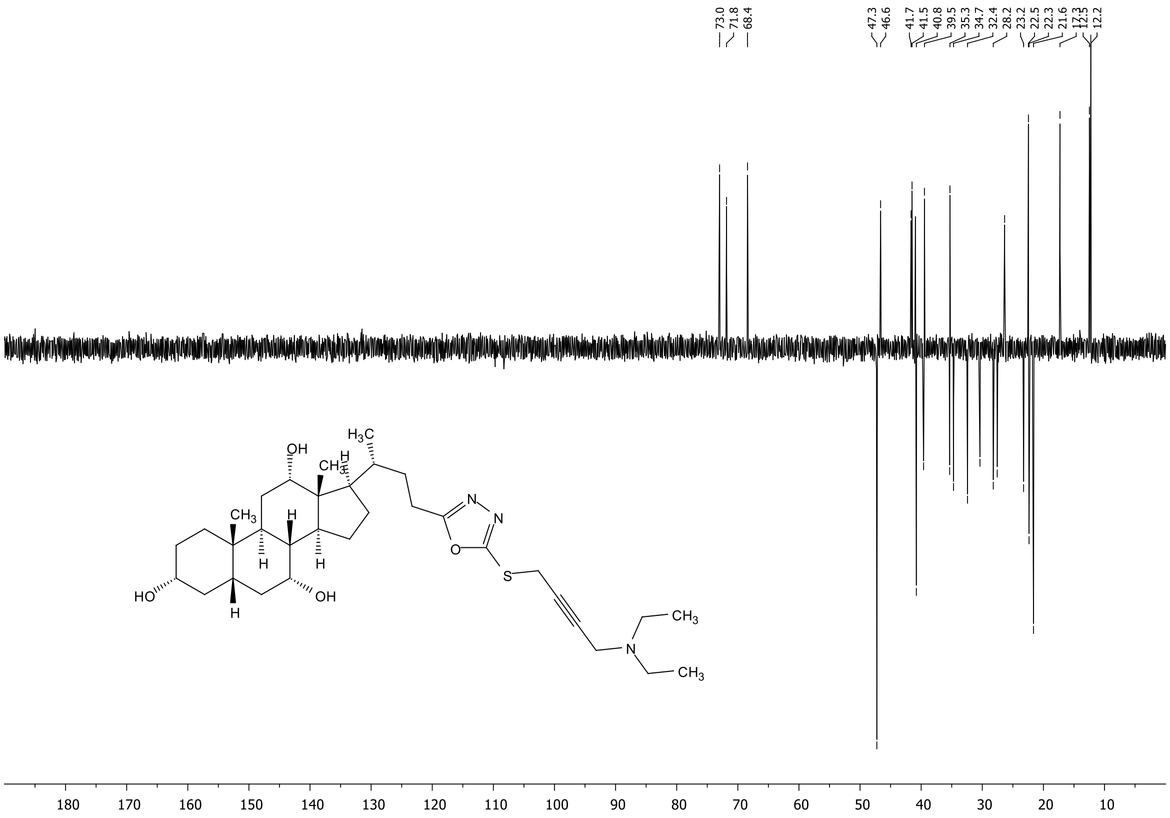
73.0
71.8
68.4
47.3
46.6
41.7
41.5
40.8
39.5
35.3
34.7
32.4
28.2
23.2
22.5
22.3
21.6
17.3
12.5
12.2
180
170
160
150
140
130
120
110
100
90
80
70
60
50
40
30
20
10

Compound 4s

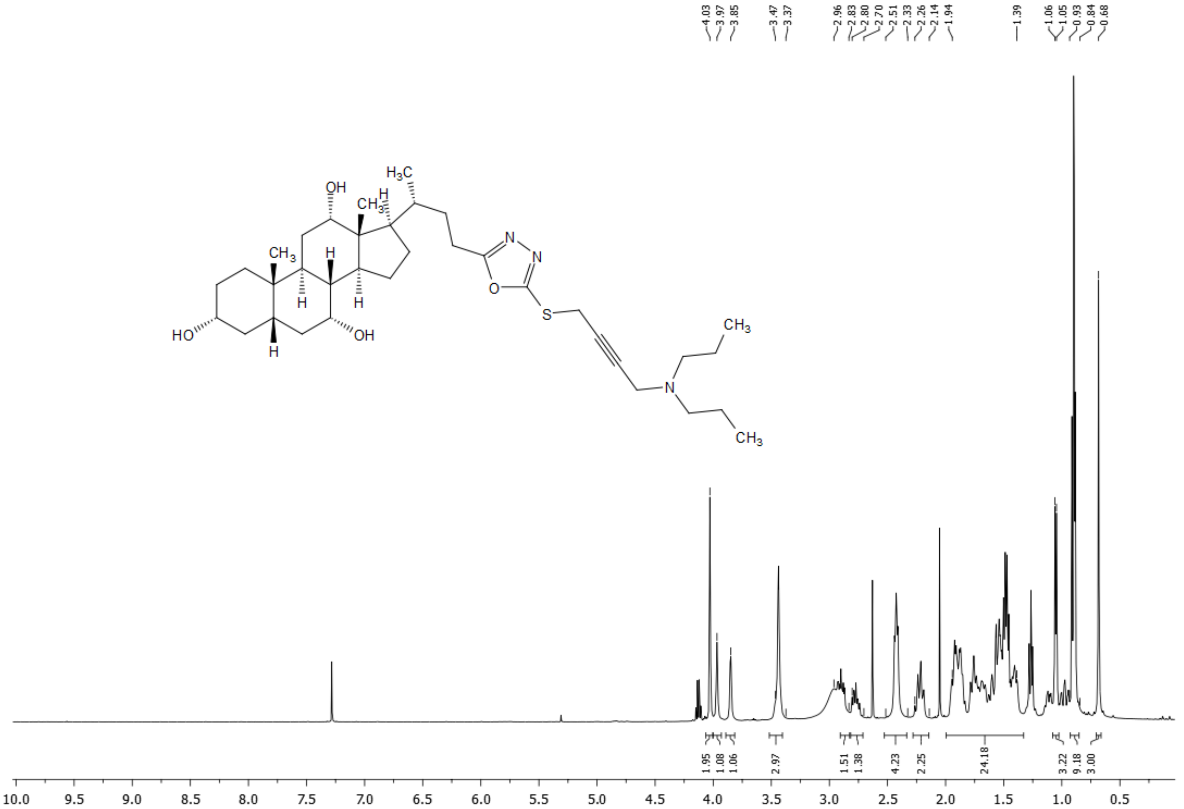

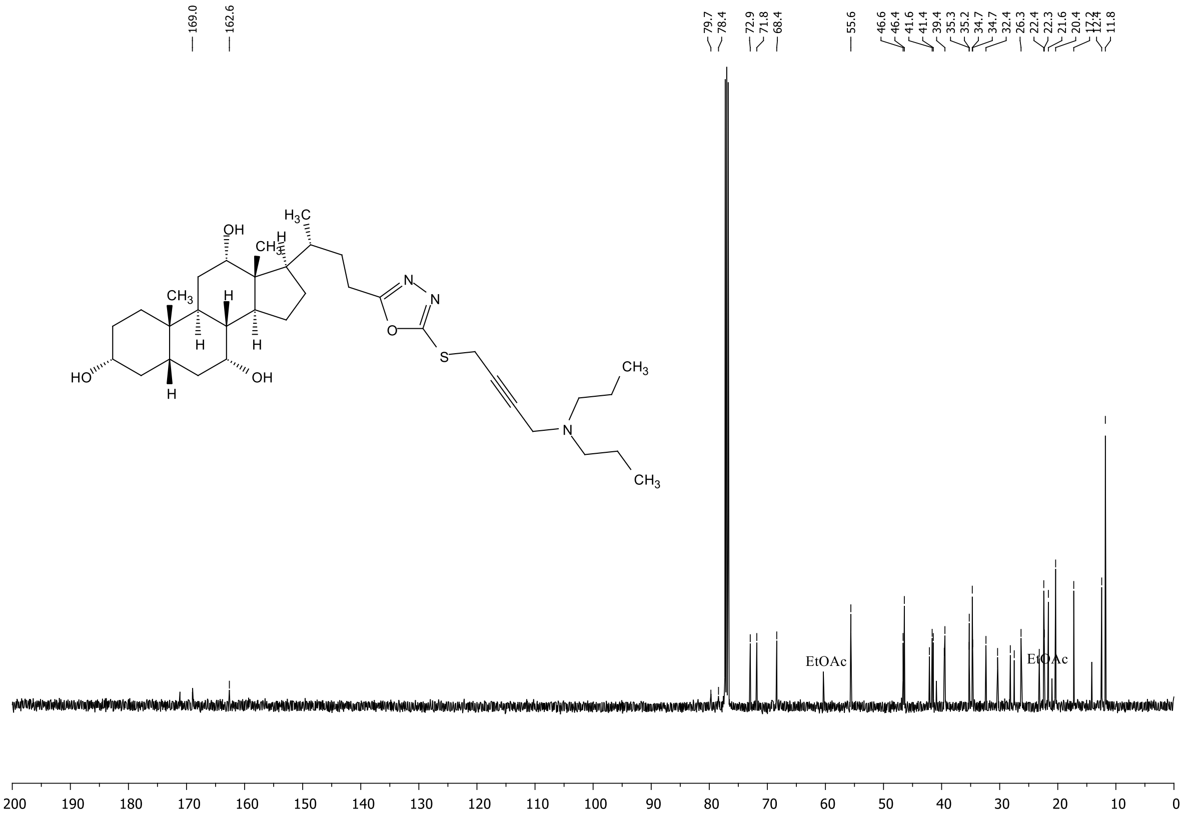

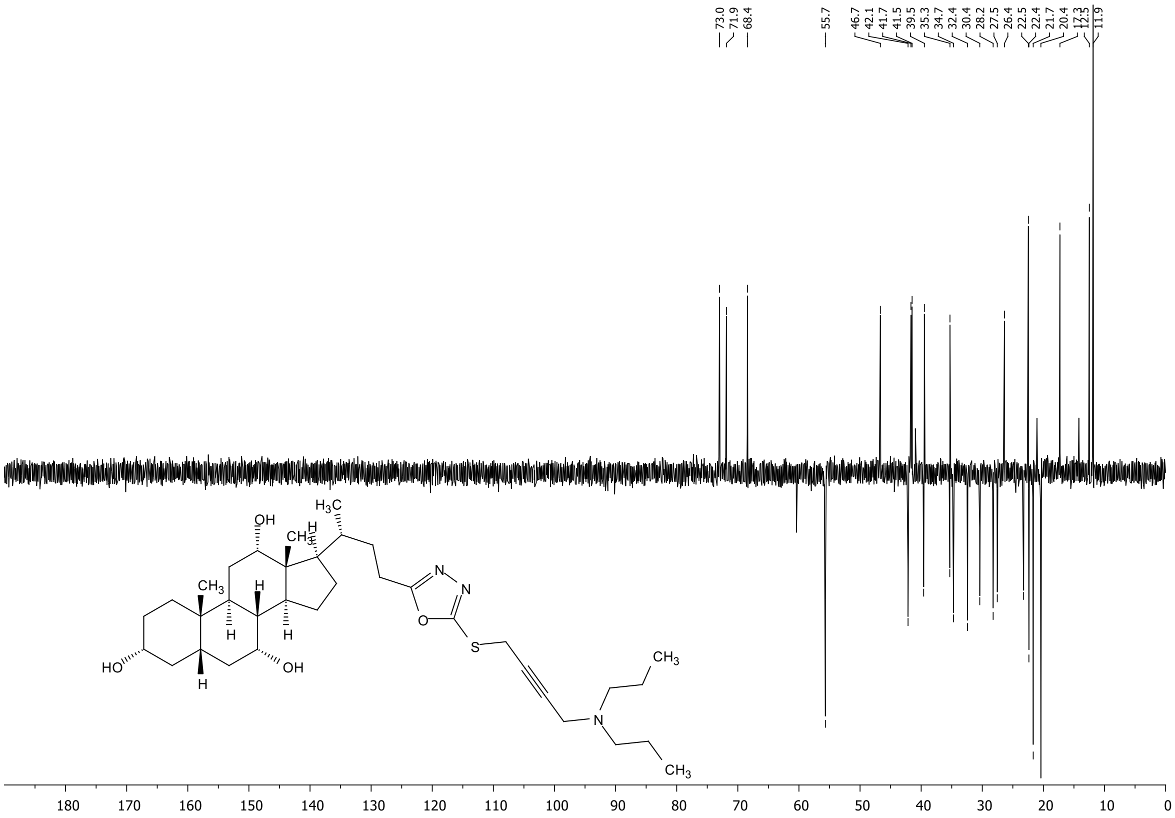

73.0
71.9
68.4
55.7
46.7
42.1
41.7
41.5
39.5
35.3
34.7
32.4
30.4
28.2
27.5
26.4
22.5
22.4
21.7
20.4
17.3
12.5
11.9
180
170
160
150
140
130
120
110
100
90
80
70
60
50
40
30
20
10
0

Compound 4t

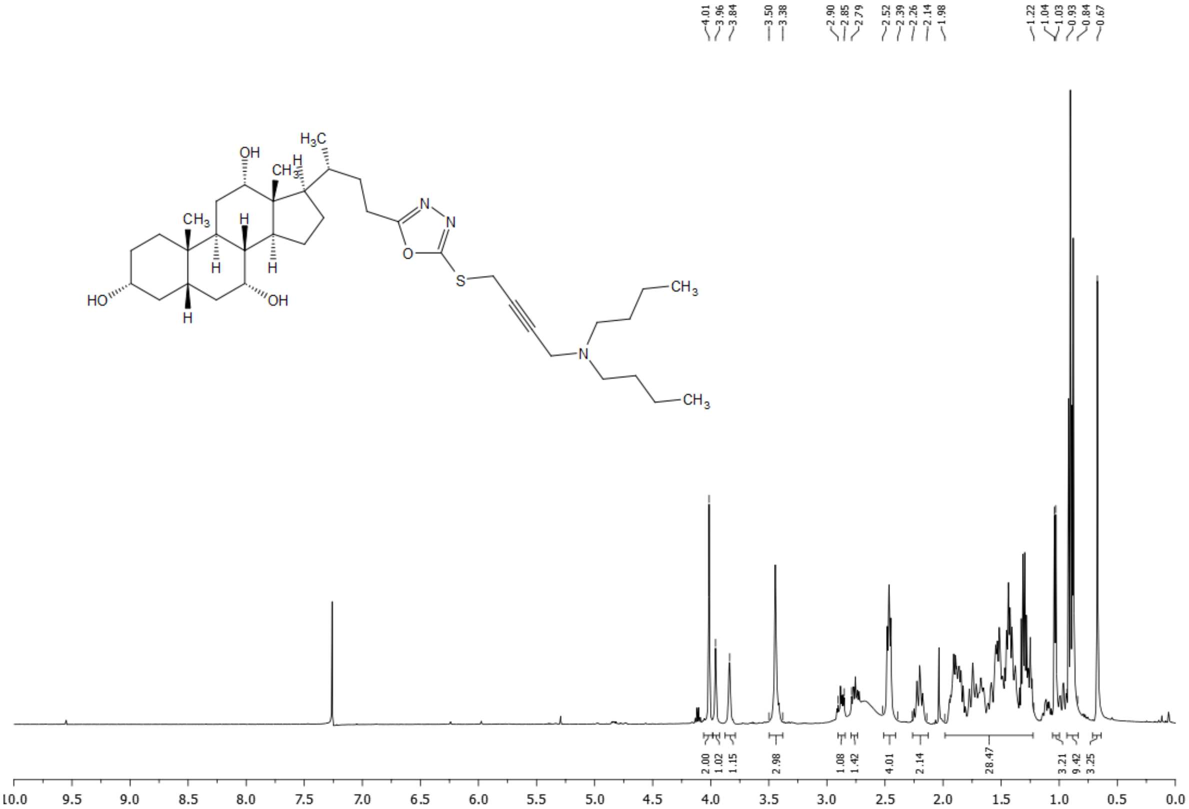
4.01
3.96
3.84
3.50
3.38
2.90
2.85
2.79
2.52
2.39
2.26
2.14
1.98
1.22
1.04
1.03
0.93
0.84
0.67
2.00
1.02
1.15
2.98
1.08
1.42
4.01
2.14
28.47
3.21
9.42
3.25
10.0 9.5 9.0 8.5 8.0 7.5 7.0 6.5 6.0 5.5 5.0 4.5 4.0 3.5 3.0 2.5 2.0 1.5 1.0 0.5 0.0

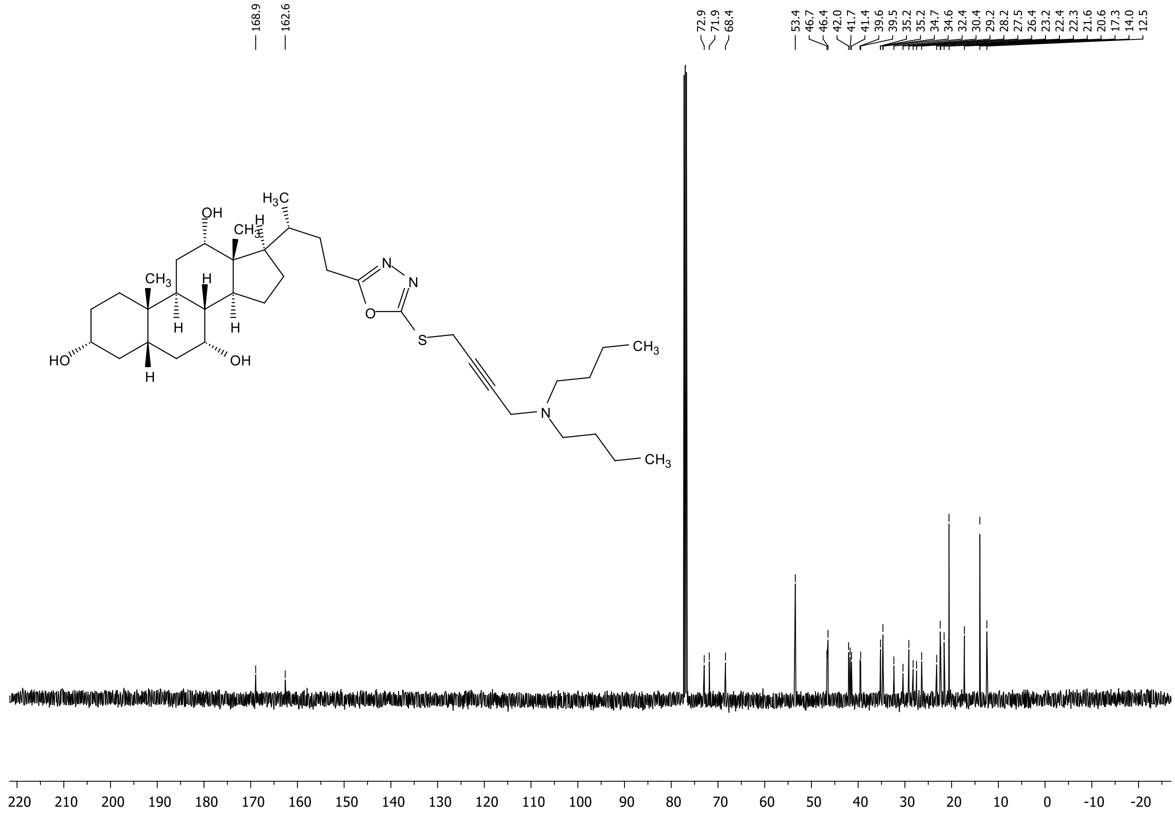
168.9
162.6
72.9
71.9
68.4
53.4
46.7
46.4
42.0
41.7
41.4
39.6
39.5
35.2
35.2
34.7
34.6
32.4
30.4
29.2
28.2
27.5
26.4
23.2
22.4
22.3
21.6
20.6
17.3
14.0
12.5
220 210 200 190 180 170 160 150 140 130 120 110 100 90 80 70 60 50 40 30 20 10 0 -10 -20

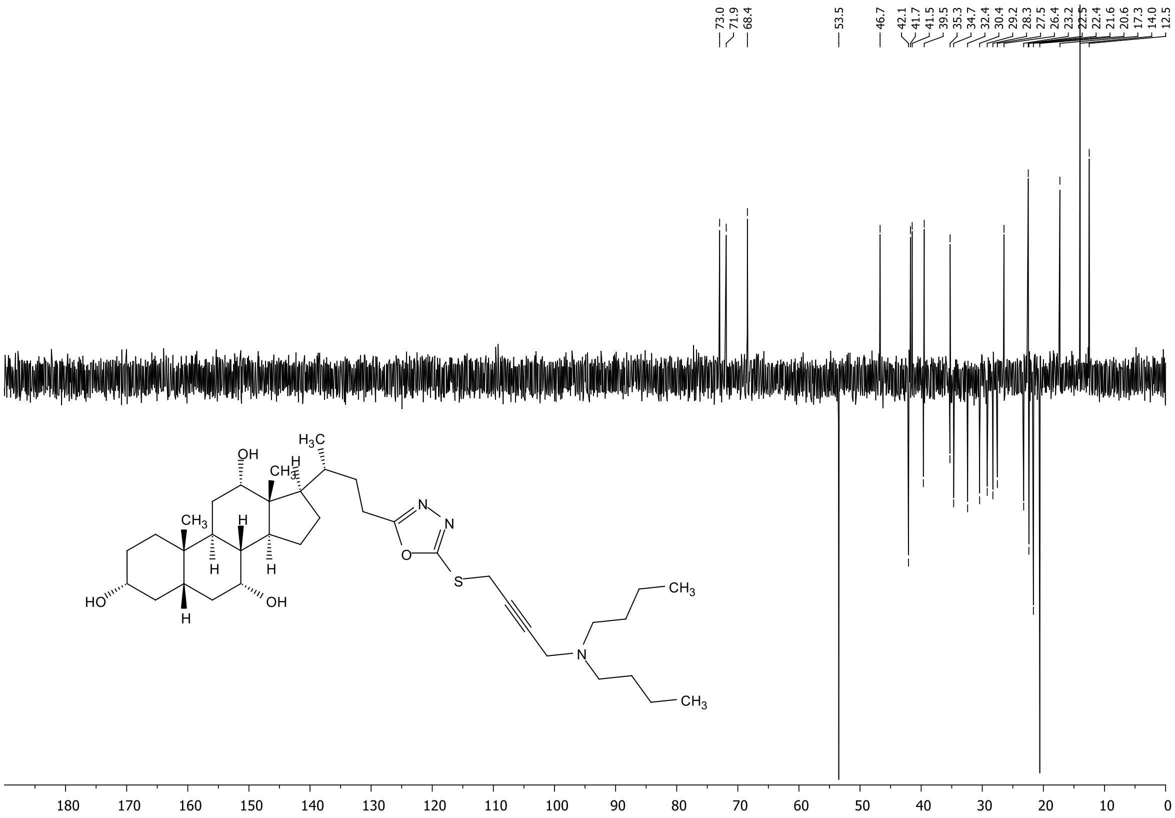
73.0
71.9
68.4
53.5
46.7
42.1
41.7
41.5
39.5
35.3
34.7
32.4
30.4
29.2
28.3
27.5
26.4
23.2
22.5
22.4
21.6
20.6
17.3
14.0
12.5
180 170 160 150 140 130 120 110 100 90 80 70 60 50 40 30 20 10 0

Compound 4u

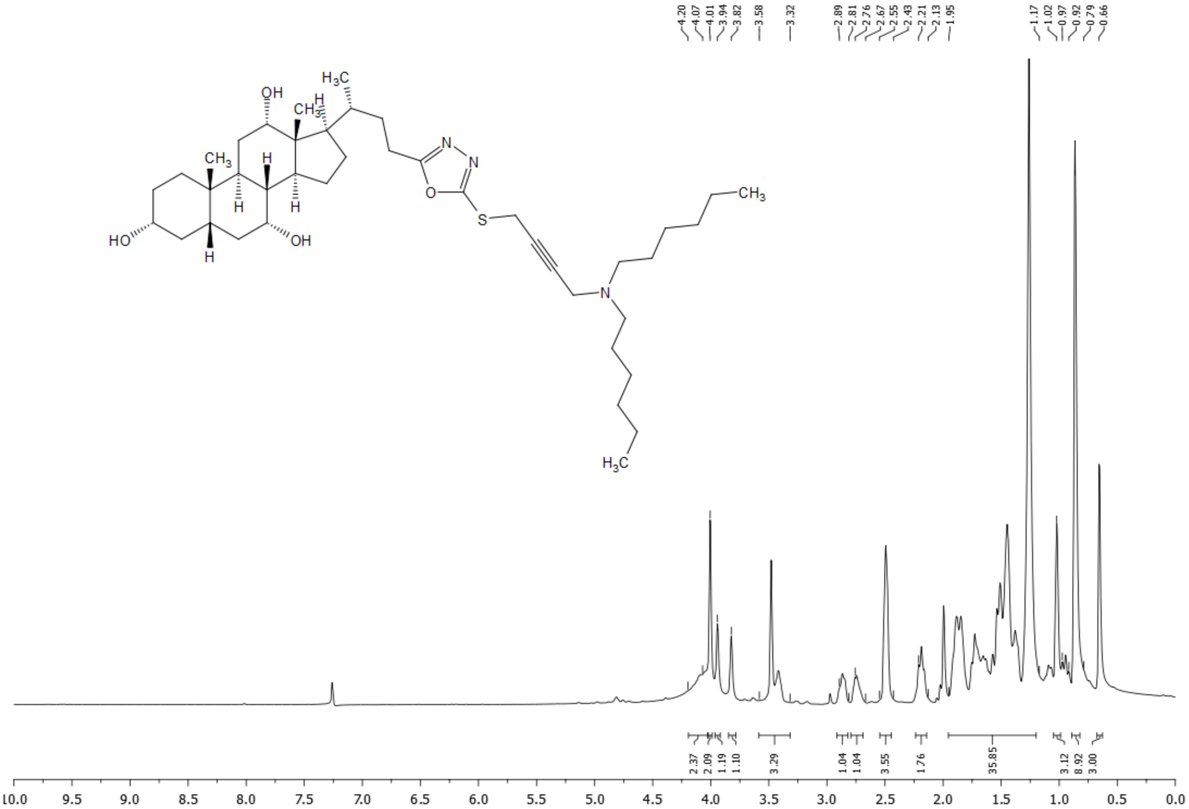
4.20
4.07
4.01
3.94
3.82
3.58
3.32
2.89
2.81
2.76
2.67
2.55
2.43
2.21
2.13
1.95
1.17
1.02
0.97
0.92
0.79
0.66
2.37
2.09
1.19
1.10
3.29
1.04
1.04
3.55
1.76
35.85
3.12
8.92
3.00
10.0 9.5 9.0 8.5 8.0 7.5 7.0 6.5 6.0 5.5 5.0 4.5 4.0 3.5 3.0 2.5 2.0 1.5 1.0 0.5 0.0

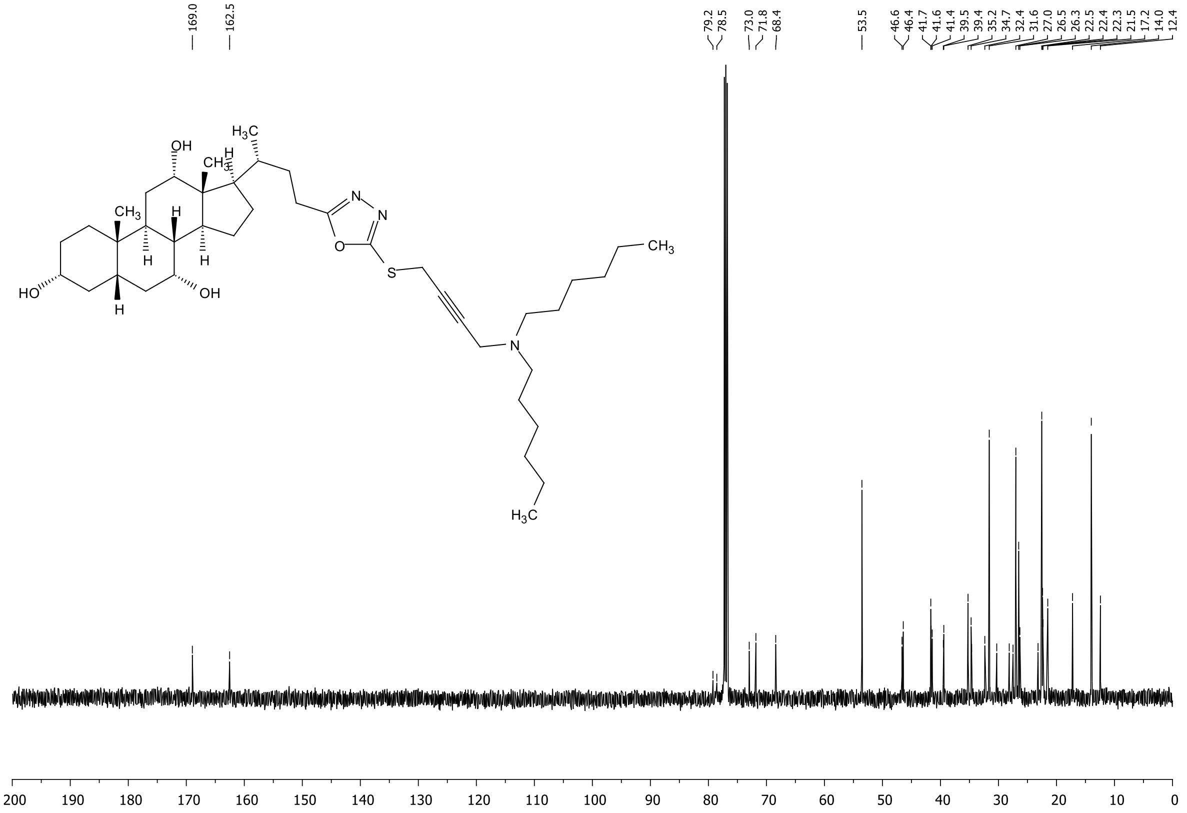
169.0
162.5
79.2
78.5
73.0
71.8
68.4
53.5
46.6
46.4
41.7
41.6
41.4
39.5
39.4
35.2
34.7
32.4
31.6
27.0
26.5
26.3
22.5
22.4
22.3
21.5
17.2
14.0
12.4
200 190 180 170 160 150 140 130 120 110 100 90 80 70 60 50 40 30 20 10 0

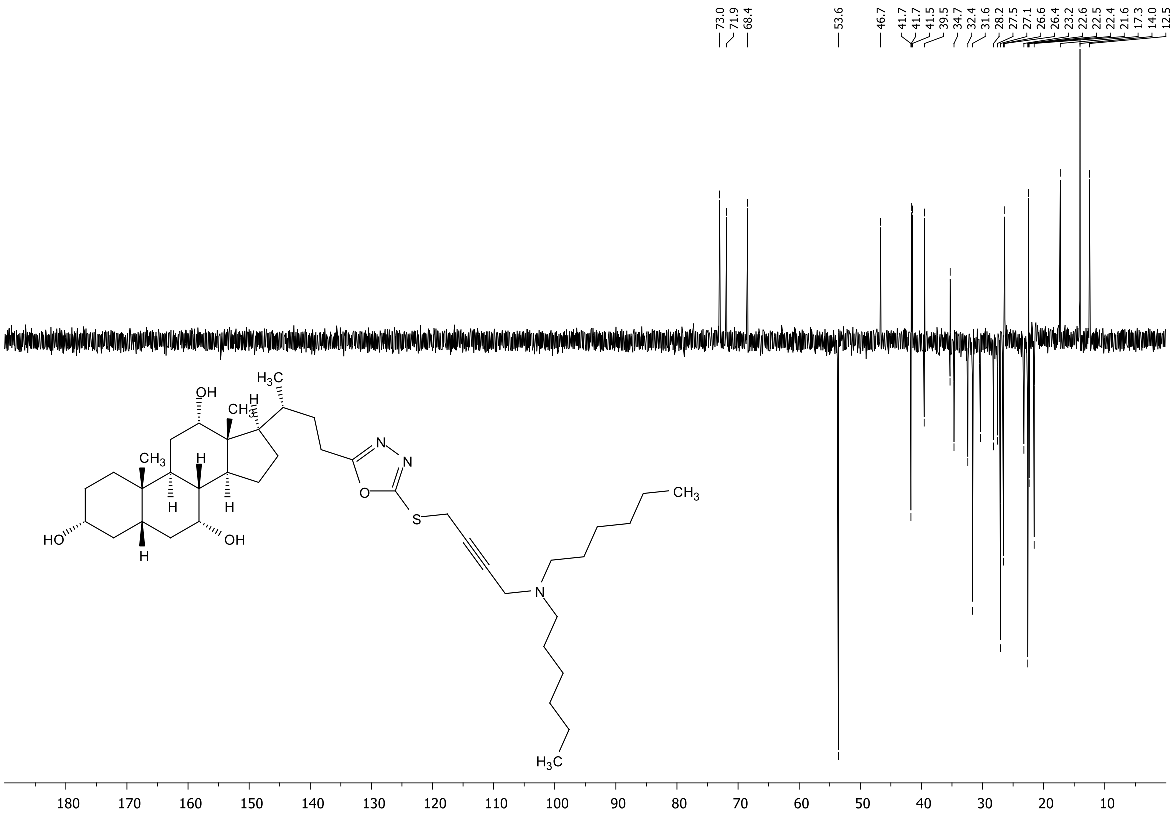
73.0
71.9
68.4
53.6
46.7
41.7
41.7
41.5
39.5
34.7
32.4
31.6
28.2
27.5
27.1
26.6
26.4
23.2
22.6
22.5
22.4
21.6
17.3
14.0
12.5
180
170
160
150
140
130
120
110
100
90
80
70
60
50
40
30
20
10

Compound 4v

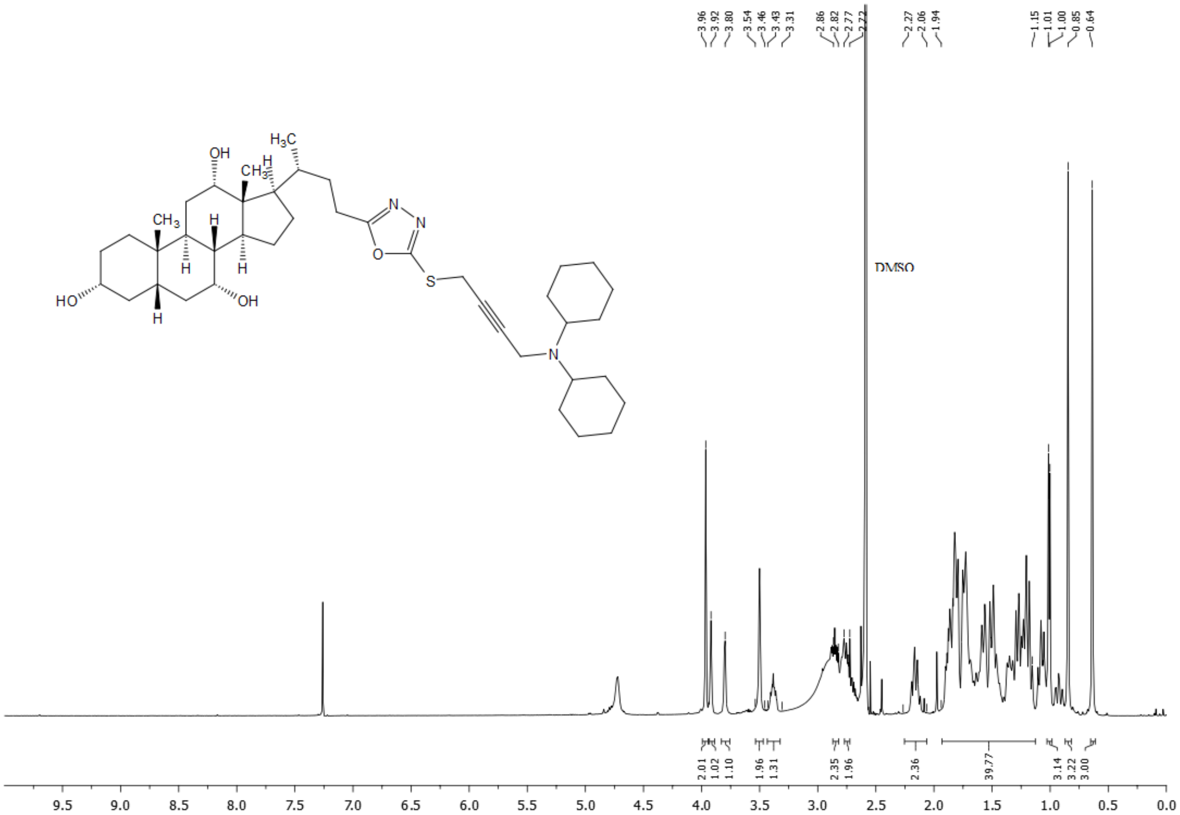
3.96
3.92
3.80
3.54
3.46
3.43
3.31
2.86
2.82
2.77
2.72
2.27
2.06
1.94
1.15
1.01
1.00
0.85
0.64
DMSO
2.01
1.02
1.10
1.96
1.31
2.35
1.96
2.36
39.77
3.14
3.22
3.00
9.5
9.0
8.5
8.0
7.5
7.0
6.5
6.0
5.5
5.0
4.5
4.0
3.5
3.0
2.5
2.0
1.5
1.0
0.5
0.0

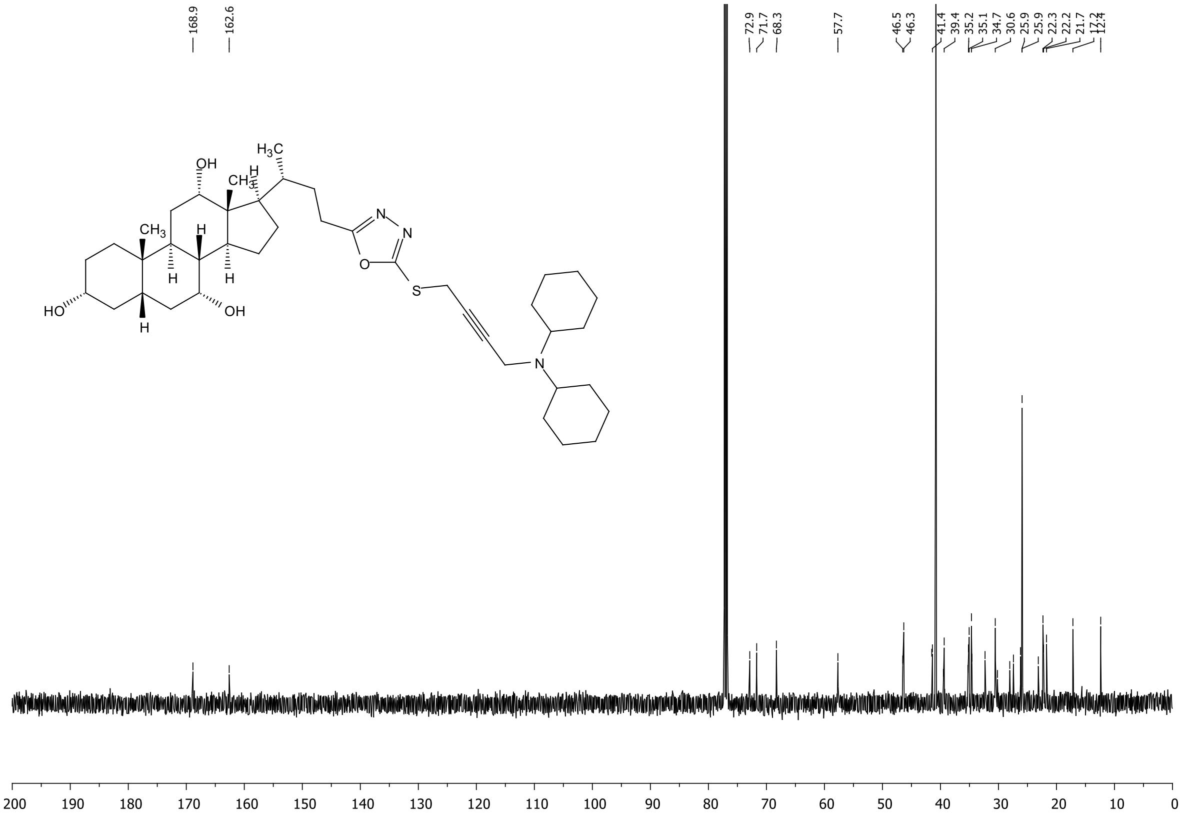
168.9
162.6
72.9
71.7
68.3
57.7
46.5
46.3
41.4
39.4
35.2
35.1
34.7
30.6
25.9
25.9
22.3
22.2
21.7
17.2
12.4
200
190
180
170
160
150
140
130
120
110
100
90
80
70
60
50
40
30
20
10
0

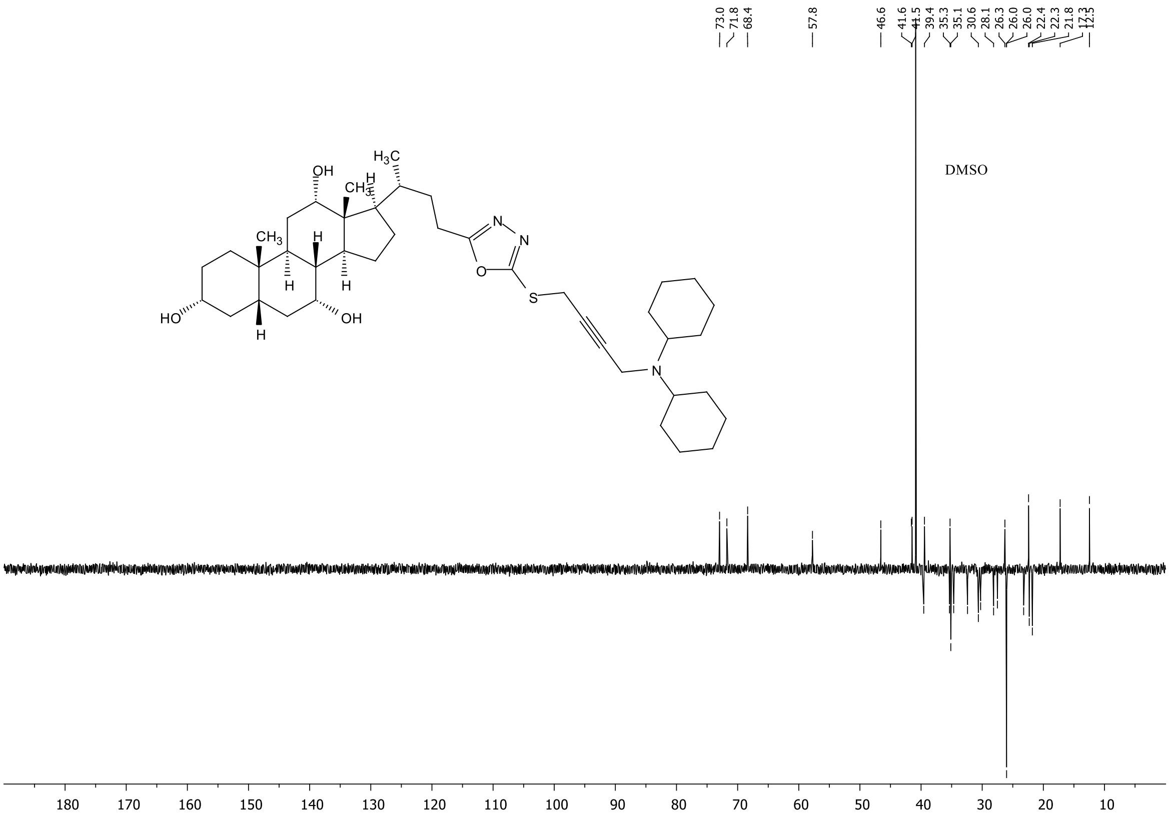
73.0
71.8
68.4
57.8
46.6
41.6
41.5
39.4
35.3
35.1
30.6
28.1
26.3
26.0
26.0
22.4
22.3
21.8
17.3
12.5
DMSO
180
170
160
150
140
130
120
110
100
90
80
70
60
50
40
30
20
10

4. References

1. Abbas, I.; Gomha, S.; Elneairy, M.; Elaasser, M.; Mabrouk, B. *Turkish Journal of Chemistry* **2015**, *39*, 510-531.
2. Abo-Ashour, M. F.; Eldehna, W. M.; George, R. F.; Abdel-Aziz, M. M.; Elaasser, M. M.; Gawad, N. M. A.; Gupta, A.; Bhakta, S.; Abou-Seri, S. M. *Eur. J. Med. Chem.* **2018**, *160*, 49-60.
